# Total Syntheses and Antibacterial Studies of Natural Isoflavones: Scandenone, Osajin, and 6,8-Diprenylgenistein

**DOI:** 10.3390/molecules29112574

**Published:** 2024-05-30

**Authors:** Hongbo Dong, Yufei Che, Xingtong Zhu, Yi Zhong, Jiafu Lin, Jian Wang, Weihong Du, Tao Song

**Affiliations:** 1 Anti-Infective Agent Creation Engineering Research Centre of Sichuan Province, School of Pharmacy, Chengdu University, Chengdu 610106, China; donghongbo@cdu.edu.cn (H.D.); cheyufei2023@163.com (Y.C.); zhongyi@stu.cdu.edu.cn (Y.Z.); linjiafu@cdu.edu.cn (J.L.); wangjian@cdu.edu.cn (J.W.); 2 School of Food Science and Biological Engineering, Zhejiang Gongshang University, Hangzhou 310018, China; zxtttt2000@163.com

**Keywords:** isoflavones, natural products, total synthesis, antibacterial activity

## Abstract

Isoflavones are a class of natural products that exhibit a wide range of interesting biological properties, including antioxidant, hepatoprotective, antimicrobial, and anti-inflammatory activities. Scandenone (**1**), osajin (**2**), and 6,8-diprenylgenistein (**3**) are natural prenylated isoflavones that share the same polyphenol framework. In this research, the key intermediate **15** was used for the synthesis of the natural isoflavones **1**–**3**, establishing a stereoselective synthetic method for both linear and angular pyran isoflavones. The antibacterial activities of **1**–**3** were also evaluated, and all of them displayed good antibacterial activity against Gram-positive bacteria. Among them, **2** was the most potent one against MRSA, with a MIC value of 2 μg/mL, and the SEM assay indicated that the bacterial cell membranes of both MRSA and *E. faecalis* could be disrupted by **2**. These findings suggest that this type of isoflavone could serve as a lead for the development of novel antibacterial agents for the treatment of Gram-positive bacterial infections.

## 1. Introduction

**Figure 1:**
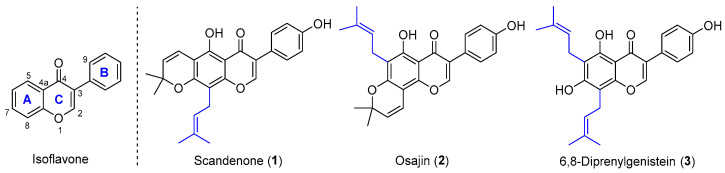
The Structures of Isoflavone and Compounds **1**–**3**.

Isoflavones are plant secondary metabolites characterized by a B-ring attached to the C-3 position of their C-ring (Figure 1) [1,2]. They exhibit a range of biological properties, including antioxidant [3], hepatoprotective [4], antimicrobial [5], and anti-inflammatory [6] activities. Prenylated isoflavones in particular have pronounced pharmacological effects due to their higher lipophilicity and affinity for biological cell membranes. Scandenone (**1**) is a natural prenylated isoflavone that has been mainly isolated from the Fabaceae and Moraceae plant families [7,8]. Till now, the biological activities of scandenone (**1**) have been extensively studied. It is a powerful protein kinase inhibitor and displays potent anti-inflammatory activity [9]. The antiviral [7], antibacterial [10], and insecticidal properties [11,12] of scandenone (**1**) have also been discussed in previously published papers. In 2006, Toker reported that scandenone (**1**) from the fruits of *Maclura pomifera* (Rafin.) *Schnider* (Moraceae) possessed good antibacterial activity against both Gram-positive (*Staphylococcus aureus* MIC = 0.5 μg/mL, *Enterococcus faecalis* MIC = 0.5 μg/mL) and Gram-negative (*Escherichia coli*, MIC = 2 μg/mL) bacterial strains [10]. In 2018, Raksat et al. found that scandenone (**1**) **1** from *Millettia extensa* displayed promising antibacterial activity against *S. aureus* TISTR 1466, *S. epidermidis* ATCC 12228, and *B. subtilis* TISTR 008 with the same MIC value of 2 μg/mL [13]. However, Ramasami et al. found that scandenone (**1**) from *Erythrina addisoniae* did not exhibit significant antibacterial activity against *S. aureus* or *E. coli* with MIC values > 64 μg/mL [14]. These inconsistencies may be due to the different levels of purity of **1** that were exacted from various natural sources, and they suggest that rigorous antibacterial evaluation is needed to determine its authentic activity.

Osajin (**2**) and 6,8-diprenylgenistein (**3**) (Figure 1) are natural prenylated isoflavones, that contain very close chemical structures to scandenone (**1**) and have been investigated over the years for their anticancer [15], anti-inflammatory [16], and antioxidant properties [17]. In particular, 6,8-diprenylgenistein (**3**) isolated from the roots of *Glycyrrhiza uralensis* has been reported to display remarkable antibacterial activity against *Streptococcus mutans* (MIC = 2 μg/mL) and MRSA (MIC = 8 μg/mL) [18]. We have recently become interested in exploring the pharmacological potential of flavonoids [19,20,21,22]. However, isoflavones **1**–**3** could only be obtained from the respective natural sources in low yields, insufficient for further detailed investigation. We therefore initiated a program to synthesize isoflavones **1**–**3** to investigate their antibacterial properties.

## 2. Results and Discussions

**Scheme 1:**
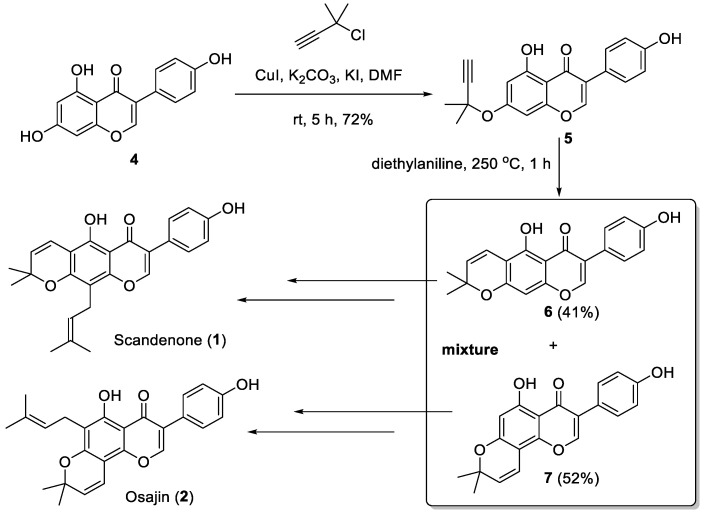
Reported Synthetic Approaches for Isoflavones **1** and **2**.

To our surprise, it was not until recently that the first total synthesis of Scandenone (**1**) and Osajin (**2**) was reported. As demonstrated in Scheme 1, Wang et al. [23] reported a chemoselective propargylation followed by a Claisen rearrangement to construct the crucial pyran isoflavone cores (compounds **6** and **7**) of isoflavones **1** and **2**. However, in our hands, the chemoselective propargylation only gave about 15% yield of pure **5**, and the separation of the highly polar compounds **6** and **7** by chromatography was very difficult. To our knowledge, no synthetic approach to 6,8-diprenylgenistein (**3**) has been reported. The above-mentioned reasons were the impetus for our current efforts to synthesize the prenylated isoflavones **1**–**3** and to compare their antibacterial activities.

**Scheme 2:**
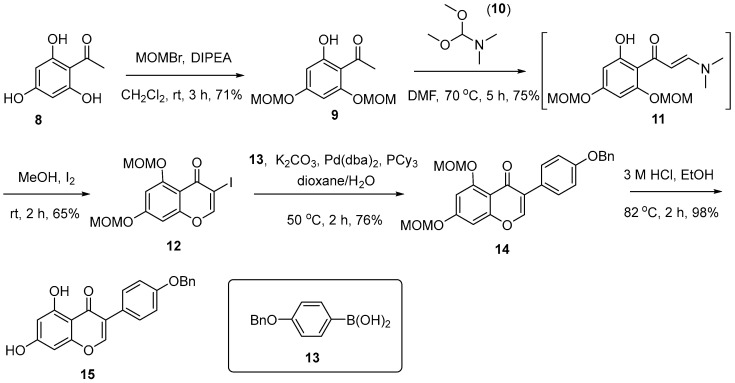
Synthesis of the Key Intermediate **15**.

As shown in Scheme 2, we started our synthesis to afford natural compounds **1**–**3** by making a known intermediate, 3-iodochromone **12** [24]. Using previously published procedures, the selective protection of two hydroxyl groups in **8** with methoxymethyl bromide (MOMBr) and *N*,*N*-Diisopropylethylamine (DIPEA) provided MOM ether **9** (71%) [25], which was treated with dimethylacetal (**10**) in DMF to furnish enamino ketone **11** (75%). Enaminone **11** was subsequently subjected to intramolecular cyclization using I_2_ in MeOH at rt to give 3-iodochromone **12** (65%). We then utilized a palladium-catalyzed Suzuki reaction with **12** and phenylboronic acid **13** to give the desired isoflavone **14** in 76% yield [26,27]. Removal of the MOM groups of **14** provided the desired 5,7-dyhydroxyl-isoflavone derivative **15** in 98% yield [25].

With the key intermediate **15** in hand, we next investigated the di-prenylation of **15** with 1-bromo-3-methylbut-2-ene (**17**) or 3-methylbut-2-en-1-ol (**18**) to get compound **16**. As shown in Table 1, we first tested previously reported conditions that used **17** as a reagent, the mixture of CH_2_Cl_2_ and H_2_O as a solvent, and KOH as a base (entry 1) [28]. However, when we applied these conditions to get **16**, an inseparable mixture of products was obtained, and no **16** could be isolated. Replacing KOH with a weaker base such as K_2_CO_3_ and DBU [29,30], only afforded the desired product **16** in low yields (entry 2, 3). The unsuccessful attempts are likely attributable to the simultaneous occurrence of *C*- and *O*-prenylations during the reaction. To avoid the *O*-prenylation, we then turned to imply 3-methylbut-2-en-1-ol (**18**) as a reagent and appropriate organic acids as catalysts in hydrophobic solvents to obtain **16**. After a few attempts, we found that the use of *p*-toluenesulfonic acid in CH_2_Cl_2_ could generate **16** in 14% yield (entry 4). Finally, **16** could be obtained in a satisfactory isolated yield (72%) using acidic alumina as a catalyst and 1,2-dichloroethane (DCE) as a solvent (entry 5) [31,32,33]. Compound **16** was treated with newly prepared sodium dodecane-1-thiolate in reflux DMF to generate natural product **3** in 78% yield [19].

As shown in Table 2, the synthesis of scandenone (**1**) and osajin (**2**) commenced with the stereocontrolled preparations of tetracyclic isoflavens **19** and **20**, which possessed a linear or angular pyran attached to the A-ring, respectively. Our first attempt was to synthesize **19** and **20** in a one-step pyran annulation of **15**. As depicted in Table 2 (entry 1 and 2), electrocyclization of **15** with *α*,*β*-unsaturated aldehyde (**21**) [34] or 1,1-diethoxy-3-methylbut-2-ene (**22**) [25] could only provide a mixture of linear and angular isomers (**19** and **20**). To overcome this problem, the Claisen rearrangement/cyclization reaction was explored for the stereoselective construction of the pyran rings of both **19** and **20**. Treatment of **15** with 3-chloro-3-methylbut-1-yne (**23**) and K_2_CO_3_ in DMF in the presence of a catalytic amount of KI afforded C-7 propargyl ether **24** in a good yield (79%). As shown in Table 2, an intensive screening of reaction conditions was carried out to identify suitable reaction conditions that would lead to the regioselective Claisen rearrangement or cyclization of **24**. It was observed that the solvent of the reaction significantly affected the regioselectivity. Aromatic Claisen rearrangement or cyclization in DMF afforded **20** as the main product (77%, entry 3), while in xylene, **19** was isolated as the main product in a yield of 55% (entry 4). We then found that the addition of KOH to xylene could effectively control the regioselectivity, and pure **19** was obtained in 93% yield (entry 5) [33,35], whereas the reported Claisen rearrangement/cyclization conditions of **15** only yielded a roughly 1:1 mixture of **19** and **20** under harsh conditions (entry 6) [23].

**Figure 2:**
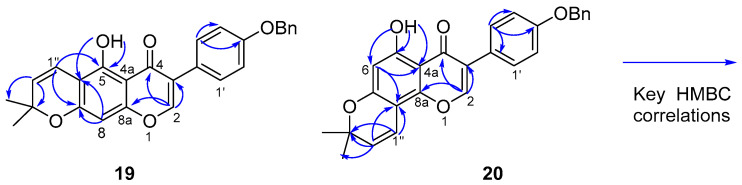
Key HMBC correlations of **19** and **20**.

Since the NMR data of 3-(4-(benzyloxy)phenyl)-5-hydroxy-8,8-dimethyl-4*H*,8*H*-pyrano[2,3-*f*]chromen-4-one (**19**) and (4-(Benzyloxy)phenyl)-5-hydroxy-8,8-dimethyl-4*H*,8*H*-pyrano[2,3-*f*]chromen-4-one (**20**) have not been reported yet; the distinction between the two compounds was challenging and may lead to misassignment due to their structural similarity. We therefore employed 2D NMR spectroscopy to unequivocally confirm the structures of **19** and **20**. The HSQC data established all ^1^*J* (^1^H-^13^C) connectivities (see the Supplementary Materials), and the key HMBC correlations are shown in Figure 2. The HMBC spectra of **19** showed a correlation of 5-OH [*δ*_H_ 13.18] with C-6 [*δ*_C_ 105.0], while there was no correlation of 5-OH with the tertiary carbon atom [C-8 (*δ*_H_ 6.34, *δ*_C_ 93.8)], suggesting that **19** is a linear isomer. The HMBC correlations between 5-OH [*δ*_H_ 12.95] with C-4a [*δ*_C_ 105.0] and tertiary carbon C-6 [*δ*_H_ 6.30, *δ*_C_ 99.3] of **20** indicated that it was an angular isomer.

**Scheme 3:**
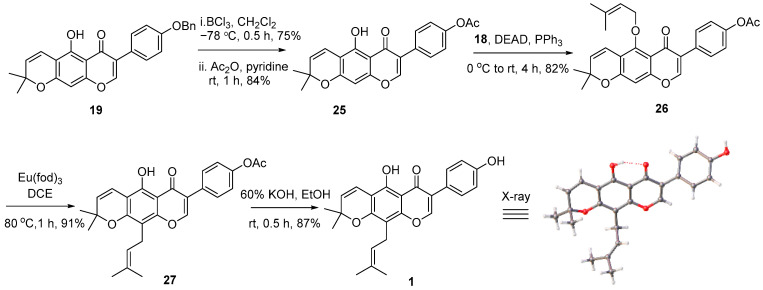
Synthesis of Scandenone (**1**).

Since selective debenzylation is difficult in the presence of both pyran and isopentene groups, the benzyl group in **19** was removed and replaced by the acetyl protecting group to give compound **25**, which was then subjected to condensation with **18** under typical Mitsunobu conditions using triphenylphosphine (PPh_3_) and diethyl azodicarboxylate (DEAD) to obtain **26** (Scheme 3). Compound **26** was subjected to a europium(III)-tris(1,1,1,2,2,3,3-heptafluoro-7,7-dimethyl-4,6-octanedionate) [Eu(fod)_3_]-catalyzed aromatic *para*-Claisen-Cope rearrangement conditions to afford **27** (91%) [34]. Deprotection of the -Ac group with 60% KOH in EtOH completed the synthesis of scandenone (**1**) (87%), the structure of which was confirmed by single-crystal X-ray analysis (see the Supplementary Materials). Our X-ray analysis data was in line with previously published results [14].

**Scheme 4:**
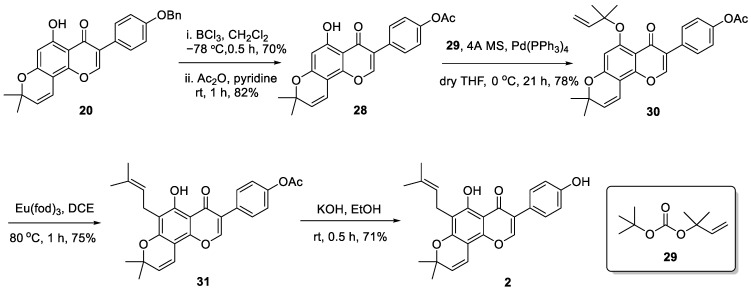
Synthesis of Osajin (**2**).

In the same manner, compound **20** was smoothly translated into Ac-protected compound **28**, and compound **30** was obtained by Pd-catalyzed allylation of **28** with *tert*-butyl (2-methylbut-3-en-2-yl) carbonate (**29**), in the presence of Pd(PPh_3_)_4_ and 4Å molecular sieves in nitrogen atmosphere in 78% yield (Scheme 4) [34]. Subsequently, an [Eu(fod)_3_]-catalyzed aromatic *ortho*-Claisen rearrangement was taken to afford **31** in a yield of 84%. Finally, treatment of **31** with a KOH aqueous solution provided natural isoflavone **2** in 71% yield.

The target compounds (**1**–**3**) were evaluated for their in vitro antibacterial activities against three Gram-positive (G+) bacterial strains (*S. aureus* ATCC29213, MRSA ATCC33591, and *Enterococcus faecalis* ATCC29212,) and one Gram-negative (G-) bacterial strain (*E. coli* ATCC25922) using minimum inhibitory concentration (MIC) values. Ampicillin and vancomycin were used as the positive controls [33].

As shown in Table 3, the anti-bacterial activity data showed that **1**–**3** were active against three G+ bacteria, including a multidrug-resistant strain (MRSA ATCC33591), but not for G-strain *E. coli* ATCC25922, which is consistent with the results reported by Raksat et al. [13] and Nkengfack et al. [18]. Among these compounds, natural isoflavone **2** exhibited the most potent antibacterial activity against G+ bacteria, with MIC values ranging from 2 to 8 μg/mL. The MIC of natural compound **2** against *S. aureus* ATCC29213 was 2 μg/mL, which was 16-fold lower compared with the positive control, ampicillin. Remarkably, these natural flavones (**1**–**3**) gave low MIC values (2–4 μg/mL) against MRSA, which were much better than ampicillin. Previously published work indicated that the antibacterial activity of natural flavonoids against G+ bacteria depends on balanced lipophilicity [36]. Compared to the parent isoflavone, prenylated isoflavones **1**–**3** have a higher lipophilicity, which may endow them with a higher antibacterial activity against G+ bacteria. Therefore, compound **2** displayed the most potent antibacterial activity due to its well-balanced lipophilicity [37].

**Figure 3:**
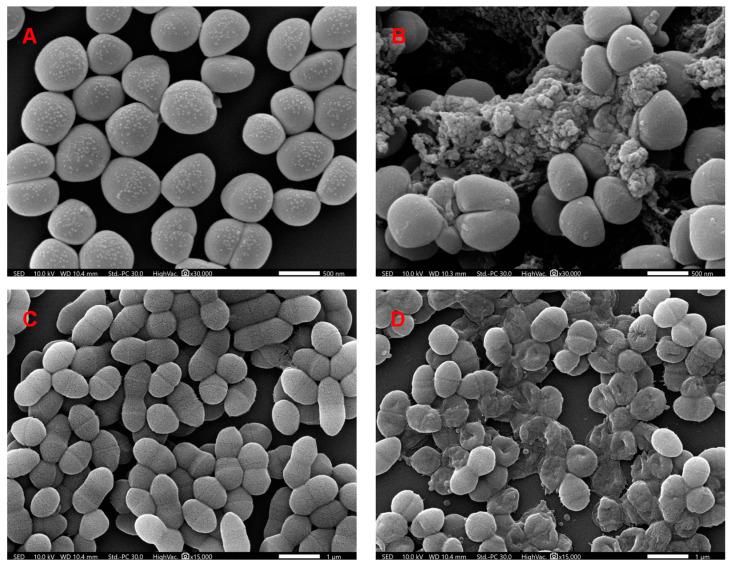
SEM images of MRSA ATCC33591 and *E. faecalis* ATCC29212 bacterial cells. (**A**) MRSA ATCC33591 cells without treatment; scale bar: 500 nm. (**B**) MRSA ATCC33591 cells treated with 8 × MIC of compound **2**, scale bar: 500 nm. (**C**) *E. faecalis* ATCC29212 cells without treatment; scale bar: 1 μm. (**D**) *E. faecalis* ATCC29212 cells treated with 8 × MIC of compound **2**, scale bar: 1 μm.

To further confirm the antibacterial activity of Osajin (**2**), we subsequently performed a scanning electron microscopy (SEM) assay [32,33], to image both MRSA ATCC33591 and *E. faecalis* ATCC29212 after incubation with **2** for 2 h. As shown in Figure 3, the untreated control groups (Figure 3A,C) had an intact cell structure and surface morphology, while treatment of MRSA ATCC33591 and *E. faecalis* ATCC29212 with **2** at a concentration of 8 × its MIC resulted in a rough and wrinkled cell membrane surface (Figure 3B,D), indicating that the bacterial cell membrane was disrupted by **2**. These findings suggested that Osajin (**2**) could inhibit bacterial growth by disrupting the integrity of the cell membrane.

## 3. Materials and Methods

### 3.1. General Experimental Procedures

Melting points were recorded on a Büchi B-545 melting point apparatus (Sigma-Aldrich, St. Louis, MO, USA). Infrared (IR) spectra were recorded on a Thermo Scientific Nicolet iS5 FT-IR spectrometer (Waltham, MA, USA). ^1^H NMR, ^13^C NMR, HMBC, and HSQC spectra were recorded on a Bruker Avance 400 spectrometer (Billerica, MA, USA) or a JEOL Eclips-600 pectrometer (Akishima, Japan), and tetramethylsilane (TMS) was used as the internal reference. The HR-MS spectra were recorded by Thermo QExactive (Thermo Scientific) and Agilent 6545 LC/QTOF mass spectrometers (Santa Clara, CA, USA). The crystal structure was analyzed with Oxford X-calibur E four-circle X-ray diffractometry (Oxford Instruments, Oxford, UK). Column chromatography was performed on silica gel (100–200 mesh). Reagents were purchased from commercial sources and used as received, unless mentioned otherwise. The solvents were of analytical grade.

### 3.2. Synthesis and Characterization of the Compounds

#### 3.2.1. 1-(2-Hydroxy-4,6-bis(methoxymethoxy)phenyl)ethan-1-one (**9**)

To a stirred solution of 2,4,6-trihydroxyacetophenone (5.00 g, 29.76 mmol) in CH_2_Cl_2_ (50 mL), DIPEA (10.4 mL, 59.52 mmol) was added at room temperature, and then MOMBr (5.0 mL, 59.52 mmol) was added to the reaction mixture at 0 °C. The resulting mixture was stirred at room temperature for 3 h. The reaction mixture was acidified with 10% HCl (aq.) (15 mL). The layers were separated, and the aqueous layer was extracted with EtOAc (3 × 10 mL). The organic layer and extracts were combined, dried, and evaporated to give a red oil, which was chromatographed on silica gel (petroleum ether/EtOAc = 30/1) to give **9** (5.47 g, 21.36 mmol, 71%) as a colorless oil. ^1^H NMR (600 MHz, CDCl_3_) *δ* 6.26–6.18 (m, 2H, –Ph), 5.23 (s, 2H, –OCH_2_OCH_3_), 5.14 (s, 2H, –OCH_2_OCH_3_–), 3.49 (s, 3H, –OCH_3_), 3.44 (s, 3H, –OCH_3_), 2.62 (s, 3H, –COCH_3_); ^13^C NMR (150 MHz, CDCl_3_) *δ* 203.3 (C=O), 166.9, 163.5, 160.4, 106.9, 97.2, 94.5, 94.0 (2 × C), 56.7 (–OCH_3_), 56.5 (–OCH_3_), 33.0 (–CH_3_). The spectroscopic data corresponds to reported values [24].

#### 3.2.2. (*E*)-3-(Dimethylamino)-1-(2-hydroxy-4,6-bis(methoxymethoxy)phenyl)prop-2-en-1-one (**11**)

Compound **9** (5.47 g, 21.36 mmol) was dissolved in dry DMF (25 mL) and heated to 74 °C. After that, compound **10** (14.2 mL, 106.8 mmol) was added dropwise, and the reaction mixture was stirred for 3 h and then cooled to rt. H_2_O (150 mL) was added to the reaction solution with stirring, and solids precipitated out. The precipitate was filtered off to afford crude product **11**. The crude material was purified by flash chromatography (PE/EtOAc = 10/1) to give **11** (4.99 g, 16.06 mmol, 75%) as a bright yellow solid: ^1^H NMR (400 MHz, CDCl_3_) *δ* 15.10 (s, 1H, –OH), 7.85 (d, *J* = 12.4 Hz, 1H, =CHN(CH_3_)_2_), 6.22 (d, *J* = 12.4 Hz, 1H, –CH=CHN(CH_3_)_2_), 6.19 (d, *J* = 2.4 Hz, 1H, –Ph), 6.10 (d, *J* = 2.4 Hz, 1H, –Ph), 5.14 (s, 2H, –OCH_2_OCH_3_), 5.08 (s, 2H, –OCH_2_OCH_3_), 3.45 (s, 3H, –OCH_3_), 3.39 (s, 3H, –OCH_3_), 3.09 (s, 3H, –N(CH_3_)_2_), 2.85 (s, 3H, –N(CH_3_)_2_); ^13^C NMR (100 MHz, CDCl_3_) *δ* 190.0 (C=O), 166.9, 161.3, 158.9, 154.5, 107.0, 97.9, 96.9, 95.2, 94.4, 94.1, 56.7 (–OCH_3_), 56.3 (–OCH_3_), 45.3 (–CH_3_), 37.3 (–CH_3_). The spectroscopic data corresponds to reported values [24].

#### 3.2.3. 3-Iodo-5,7-bis(methoxymethoxy)-4*H*-chromen-4-one (**12**)

Iodine (6.12 g, 24.09 mmol) was added to propenone **11** (4.99 g, 16.06 mmol) in MeOH (50 mL), and the solution was stirred at room temperature for 2 h. The mixture was washed with saturated Na_2_S_2_O_3_ (50 mL), and the aqueous layer was extracted with EtOAc (3 × 50 mL) and dried with Na_2_SO_4_. The crude material was purified by flash chromatography (PE/EtOAc = 30/1) to give **12** (4.10 g, 10.46 mmol, 65%), light-yellowish solids: ^1^H NMR (400 MHz, CDCl_3_) *δ* 8.03 (s, 1H, –OCH=C–), 6.68 (d, *J* = 2.3 Hz, 1H, –Ph), 6.65 (d, *J* = 2.3 Hz, 1H, –Ph), 5.23 (s, 2H, –OCH_2_OCH_3_), 5.16 (s, 2H, –OCH_2_OCH_3_), 3.48 (s, 3H, –OCH_3_), 3.43 (s, 3H, –OCH_3_); ^13^C NMR (100 MHz, CDCl_3_) *δ* 171.3 (C=O), 161.6, 159.3, 158.2, 155.7, 108.8, 102.1, 96.8, 95.5, 94.4, 89.5, 56.8 (–CH_3_), 56.6 (–CH_3_). The spectroscopic data corresponds to reported values [24].

#### 3.2.4. 3-(4-Hydroxyphenyl)-5,7-bis(methoxymethoxy)-4*H*-chromen-4-one (**14**)

To a solution of the appropriate **12** (4.10 g, 10.46 mmol) in a mixture of 1,4-dioxane (35 mL) and water (15 mL), K_2_CO_3_ (4.34 g, 31.38 mmol) and **13** (2.89 g, 20.92 mmol) were added. The mixture was purged with nitrogen for 10 min. To the mixture were then added PCy_3_ (235 mg, 0.84 mmol) and Pd(dba)_2_ (241 mg, 0.43 mmol). The mixture was warmed to 50 ℃ and then stirred at this temperature for 2 h. It was then cooled to ambient temperature. An aq. saturated solution of NH_4_Cl (50 mL) was added to the mixture, and the mixture was filtered. The filter cake was dissolved in ethyl acetate (EtOAC, 30 mL), poured into water (50 mL), and extracted with EtOAc. The organic phase was combined, washed with brine, and dried with anhydrous Na_2_SO_4_. The solvent was evaporated under reduced pressure, and the residue was purified by column chromatography on silica using a PE/EtOAC mixture (3:2 (*v*/*v*)) as the eluent to afford compound **14** (3.61 g, 10.08 mmol, 76%) as a colorless oil: 1H NMR (400 MHz, DMSO-*d*_6_) *δ* 8.22 (s, 1H, –OCH=C–), 7.50–7.29 (m, 7H, –OBn and –Ph), 7.04 (d, *J* = 8.8 Hz, 2H, –Ph), 6.81 (d, *J* = 2.2 Hz, 1H, –Ph), 6.69 (d, *J* = 2.2 Hz, 1H, –Ph), 5.32 (s, 2H, –OCH_2_OCH_3_), 5.26 (s, 2H, –OCH_2_OCH_3_), 5.14 (s, 2H, –OCH_2_Ph), 3.43 (s, 3H, –OCH_3_), 3.42 (s, 3H, –OCH_3_); ^13^C NMR (150 MHz, DMSO-*d*_6_) *δ* 174.3, 161.1, 159.2, 158.5, 158.4, 151.9, 137.6, 130.8 (2 × C), 128.9 (2 × C), 128.3, 128.1 (2 × C), 125.0, 124.9, 114.9 (2 × C), 110.9, 101.9, 97.2, 95.7, 94.6, 69.6, 56.7, 56.6; HRMS (ESI) calculated for C_26_H_25_O_7_^+^ [M + H]^+^ 449.1595, found 449.1598.

#### 3.2.5. 3-(4-(Benzyloxy)phenyl)-5,7-dihydroxy-4*H*-chromen-4-one (**15**)

A solution of **14** (3.06 g, 6.84 mmol) and 3 M HCl (70 mL) in EtOH (150 mL) was stirred at 82 °C for 2 h. After the completion of the reaction, the mixture was poured into ice water and extracted with EtOAc. The organic layer was washed with a saturated NaCl solution and dried with anhydrous Na_2_SO_4_, and the solvents were removed in vacuum. The residue obtained was purified by flash column chromatography with PE/EtOAc (10:1, *v*/*v*) as the eluent to give **15** (2.43 g, 6.75 mmol, 98%) as a yellow solid: ^1^H NMR (400 MHz, DMSO-*d*_6_) *δ* 12.92 (s, 1H, –OH), 11.05 (s, 1H, –OH), 8.37 (s, 1H, –OCH=C–), 7.52–7.29 (m, 7H, –OBn and –Ph), 7.07 (d, *J* = 8.5 Hz, 2H, –Ph), 6.43 (d, *J* = 1.8 Hz, 1H, –Ph), 6.26 (d, *J* = 1.8 Hz, 1H, –Ph), 5.15 (s, 2H, –OCH_2_Ph); ^13^C NMR (100 MHz, DMSO-*d*_6_) *δ* 180.5 (C=O), 164.9, 162.4, 158.6, 158.0, 154.8, 137.5, 130.6 (2 × C), 128.9 (2 × C), 128.3, 128.1 (2 × C), 123.6, 122.3, 115.1 (2 × C), 104.9, 99.5, 94.2, 69.6. HRMS (ESI) calculated for C_22_H_17_O_5_^+^ [M + H]^+^ 361.1071, found 361.1070.

#### 3.2.6. 3-(4-(Benzyloxy)phenyl)-5,7-dihydroxy-6,8-bis(3-methylbut-2-en-1-yl)-4*H*-chromen-4-one (**16**)

Under a N_2_ atmosphere, to a suspension of compound **15** (3.20 g, 8.88 mmol), acidic Al_2_O_3_ (17.80 g), and 4 Å molecular sieves (1.00 g) in dry DCE (160 mL) was added **18** (10 eq, 9.6 mL, 88.80 mmol). The mixture was stirred at 80 ℃ for 24 h. After the reaction was completed (detected by TLC), it was cooled to ambient temperature and filtered through a Celite. The filter cake was washed with EtOAc, and the filtrate was concentrated in vacuum to obtain a residue, which was further purified by silica gel column chromatography (PE/EtOAc (25:1, v/v) to give the pure compound **16** (3.17 g, 72%) as a yellow solid: ^1^H NMR (400 MHz, CDCl_3_) *δ* 13.20 (s, 1H, –OH), 7.90 (s, 1H, –OCH=C–), 7.51–7.30 (m, 7H, –Ph and –OBn), 7.05 (d, *J* = 8.7 Hz, 2H, –Ph), 6.38 (s, 1H, –OH), 5.33–5.18 (m, 2H, –CH=C(CH_3_)_2_), 5.11 (s, 2H, –OCH_2_Ph), 3.48 (t, *J* = 6.9 Hz, 4H, –CH_2_–), 1.85 (d, *J* = 4.5 Hz, 6H, –CH_3_), 1.76 (d, *J* = 10.9 Hz, 6H, –CH_3_); ^13^C NMR (100 MHz, CDCl_3_) *δ* 180.1 (C=O), 158.5, 157.8, 156.5, 152.2, 151.4, 135.8, 134.5, 133.05, 129.1 (2 × C), 127.5 (2 × C), 126.9, 126.4 (2 × C), 122.5, 121.9, 120.5, 120.3, 113.9 (2 × C), 109.1, 104.7, 104.3, 68.99, 24.8, 24.7, 20.6 (2 × C), 16.9, 16.8. HRMS (ESI) calculated for C_32_H_33_O_5_^+^ [M + H]^+^ 497.2323, found 497.2322.

#### 3.2.7. 5,7-Dihydroxy-3-(4-hydroxyphenyl)-6,8-bis(3-methylbut-2-en-1-yl)-4*H*-chromen-4-one (**3**)

Under a N_2_ atmosphere, a solution of 30% CH_3_ONa/CH_3_OH aqueous solution (1.8 mL, 9.68 mmol) and dodecyl mercaptan (3 eq, 2.1 mL) in DMF (35 mL) was stirred at rt for 15 min. Then **16** (1.20 g, 2.42 mmol) was added to the reaction solution with stirring and refluxing at 120 °C for 24 h.After the reaction was completed (detected by TLC), 3 M HCl was added to adjust the pH to 7, and the mixture was extracted with EtOAc. The organic layers were combined, dried over anhydrous Na_2_SO_4_, and evaporated.The residue obtained was purified over flash column chromatography with PE/EtOAc (25:1, *v*/*v*) as the eluent to give the compound **3** (767 mg, 78%) as a yellow solid. ^1^H NMR (400 MHz, CDCl_3_) *δ* 13.11 (s, 1H, –OH), 7.89 (s, 1H, –OCH=C–), 7.33 (d, *J* = 8.1 Hz, 2H, –Ph), 6.82 (d, *J* = 8.1 Hz, 2H, –Ph), 6.40 (s, 1H, –OH), 5.32–5.18 (m, 2H, –CH=C(CH_3_)_2_), 3.47 (t, *J* = 6.3 Hz, 4H, –CH_2_–), 1.84 (d, *J* = 4.9 Hz, 6H, –CH_3_), 1.75 (d, *J* = 10.9 Hz, 6H, –CH_3_); ^13^C NMR (100 MHz, CDCl_3_) *δ* 180.4 (C=O), 158.6, 156.4, 155.1, 152.3, 151.7, 134.5, 133.1, 129.2 (2 × C), 122.2, 121.7, 120.4, 120.2, 114.7 (2 × C), 109.2, 104.7, 104.4, 24.8, 24.7, 20.6 (2 × C), 16.9, 16.8. HRMS (ESI) calculated for C_25_H_27_O_5_^+^ [M + H]^+^ 407.1853, found 407.1855.

#### 3.2.8. 3-(4-(Benzyloxy)phenyl)-5-hydroxy-7-((2-methylbut-3-yn-2-yl)oxy)-4*H*-chromen-4-one (**24**)

Compound **15** (2.20 g, 6.11 mmol), CuI (700 mg, 1.94 mmol), K_2_CO_3_ (1.1 eq, 300 mg, 2.13 mmol), and KI (1.2 eq, 390 mg, 7.33 mmol) were suspended in DMF (20 mL). Then **23** (1.2 eq, 240 mg, 2.33 mmol) was added dropwise, and the resulting reaction mixture was stirred for 24 h at rt. The reaction mixture was quenched by the addition of 1 mol/L aqueous HCl. The organic layer was separated and then washed with a saturated aqueous NaCl solution and dried with anhydrous Na_2_SO_4_, and the solvents were removed in vacuum. The residue obtained was purified over flash column chromatography with PE/EtOAc (40:1, *v*/*v*) as the eluent to give compound **24** (550 mg, 79%): ^1^H NMR (400 MHz, CDCl_3_) *δ* 12.69 (s, 1H, –OH), 7.78 (s, 1H, –OCH=C–), 7.40–7.22 (m, 7H –OBn and –Ph), 6.97 (d, *J* = 8.8 Hz, 2H, –Ph), 6.72 (d, *J* = 2.2 Hz, 1H, –Ph), 6.62 (d, *J* = 2.2 Hz, 1H, –Ph), 5.02 (s, 2H, –OCH_2_Ph), 2.61 (s, 1H, –C≡CH), 1.66 (s, 6H, –CH_3_); ^13^C NMR (150 MHz, CDCl_3_) *δ* 181.0 (C=O), *δ* 162.1, 162.2, 159.1, 157.4, 152.9, 136.9, 130.2 (2 × C), 128.7 (2 × C), 128.1, 127.5 (2 × C), 123.7, 123.3, 115.1 (2 × C), 106.9, 102.8, 97.3, 84.8, 75.3, 72.8, 70.1, 29.6 (2 × C). HRMS (ESI) calculated for C_27_H_23_O_5_^+^ [M + H]^+^ 427.1540, found 427.1540.

#### 3.2.9. 3-(4-(Benzyloxy)phenyl)-5-hydroxy-8,8-dimethyl-4H,8*H*-pyrano[2,3-f]chromen-4-one (**19**)

Under a N_2_ atmosphere, compound **24** (500 mg, 1.17 mmol) and KOH (391.61 mg, 6.98 mmol) were dissolved in dry *p*-xylene (25 mL) at rt, and the resulting mixture was stirred for 1 h at 130 ^o^C. After the reaction was completed, the mixture was cooled, quenched with 3 mol/L HCl, and diluted with EtOAc. The layers were separated, and the aqueous layer was extracted with EtOAc. The combined organic layers were washed with brine, dried over anhydrous Na_2_SO_4_, and concentrated in a vacuum. The residue was purified by column chromatography (silica gel, hexane/EtOAc, 80:1) to afford compound **19** (463 mg, 93%) as a yellow solid. ^1^H NMR (400 MHz, CDCl_3_) *δ* 13.18 (s, 1H, 5-OH), 7.82 (s, 1H, H-2), 7.45 (d, *J* = 8.6 Hz, 4H, H-1′, H-5′ and –Bn), 7.40 (t, *J* = 7.3 Hz, 2H, –Bn), 7.35 (d, *J* = 7.1 Hz, 1H, –Bn), 7.05 (d, *J* = 8.7 Hz, 2H, H-2′ and H-4′), 6.73 (d, *J* = 10.0 Hz, 1H, H-1″), 6.34 (s, 1H, H-8), 5.63 (d, *J* = 10.0 Hz, 1H, H-2″), 5.11 (s, 2H, –OBn), 1.48 (s, 6H, –CH_3_); ^13^C NMR (100 MHz, CDCl_3_) *δ* 179.8 (C-4), 158.5 (C-7), 157.9 (C-3′), 156.2 (C-8a), 155.9 (C-5), 151.5 (C-2), 135.8 (–Bn), 129.1 (2 × C, C-1′ and C-5′), 127.6 (2 × C, –Bn), 127.1 (C-2″), 127.0 (–Bn), 126.4 (2 × C, –Bn), 122.4 (C-6′), 122.2 (C-3), 114.4 (C-1″), 114.0 (2 × C, C-2′ and C-4′), 105.0 (C-6), 104.5 (C-4a), 93.8 (C-8), 77.0 (C-3″), 69.0 (–OCH_2_Ph), 27.3 (2 × C). HRMS (ESI) calculated for C_27_H_23_O_5_^+^ [M + H]^+^ 427.1540, found 427.1541.

#### 3.2.10. (4-(Benzyloxy)phenyl)-5-hydroxy-8,8-dimethyl-4*H*,8*H*-pyrano[2,3-*f*]chromen-4-one (**20**)

A solution of **24** (500 mg, 1.17 mmol) in DMF (25 mL) was heated to 130 °C for 1 h. After completion of the reaction (detected by TLC), the mixture was poured into ice-cold water and extracted with EtOAc. The organic layer was washed with a saturated aqueous NaCl solution and dried with anhydrous Na_2_SO_4_, and the solvents were removed in vacuum. The residue obtained was purified over flash column chromatography with PE as the eluent to give **20** (416 mg, 77%) and **19** (49 mg, 9%) that could also be obtained. Compound **20** was a yellow solid: ^1^H NMR (400 MHz, CDCl_3_) *δ* 12.95 (s, 1H, 5-OH), 7.88 (s, 1H, H-2), 7.45 (d, *J* = 8.7 Hz, 4H, H-1′, H-5′ and –Bn), 7.40 (t, *J* = 7.4 Hz, 2H, –Bn), 7.35 (dd, 1H, –Bn), 7.05 (d, *J* = 8.3 Hz, 2H, H-2′ and H-4′), 6.68 (d, *J* = 10.0 Hz, 1H, H-1″), 6.30 (s, 1H, H-6), 5.59 (d, *J* = 10.0 Hz, 1H, H-2″), 5.11 (s, 2H, –OCH_2_Bn), 1.48 (s, 6H, –CH_3_); ^13^C NMR (100 MHz, CDCl_3_) δ 179.9 (C-4), 161.2 (C-7), 158.5 (C-5), 157.9 (C-3′), 151.4 (C-2), 151.1 (C-8a), 135.8 (–Bn), 129.1 (2 × C, C-1′ and C-5′), 127.6 (2 × C, –Bn), 127.0 (–Bn), 126.4 (C-2″), 126.4 (2 × C, –Bn), 122.5 (C-6′), 122.09 (C-3), 113.9 (2 × C, C-2′ and C-4′), 113.5 (C-1″), 105.0 (C-4a), 100.1 (C-8a), 99.3 (C-6), 77.0 (C-3′’), 69.0 (–OCH_2_Bn), 27.2 (2 × C, –CH_3_). The spectroscopic data corresponds to reported values [35].

#### 3.2.11. 4-Hydroxy-7-(4-hydroxyphenyl)-2,2-dimethyl-2*H*,6*H*-pyrano[3,2-g]chromen-6-one (**6**)

Under a N_2_ atmosphere, compound **19** (900 mg, 2.11 mmol) was dissolved in CH_2_Cl_2_ (30 mL), and the mixture was cooled to −78 °C. To this solution was added BCl_3_ (2.6 mL, 1.0 M solution in toluene, 2.53 mmol), and the reaction mixture was stirred for 0.5 h at the same temperature. The reaction mixture was quenched with a mixture of saturated aqueous NaHCO_3_ solution and MeOH (*v*/*v* = 1:1) and diluted with EtOAc (100 mL). The layers were separated, and the aqueous layer was extracted with EtOAc. The combined organic layers were washed with brine, dried over anhydrous Na_2_SO_4_, and concentrated in a vacuum. The obtained residue was purified on a flash silica gel (PE/EtOAc, 25:1) to afford **6** (530 mg, 75%) as a light yellow solid. ^1^H NMR (400 MHz, DMSO-*d*_6_) *δ* 13.33 (s, 1H, –OH), 9.59 (s, 1H, –OH), 8.31 (s, 1H, –OCH=C–), 7.35 (d, *J* = 8.6 Hz, 2H, –Ph), 6.79 (d, *J* = 8.6 Hz, 2H, –Ph), 6.57 (d, *J* = 10.1 Hz, 1H, –CH=CHC(CH_3_)_2_O–), 6.43 (s, 1H, –Ph), 5.75 (d, *J* = 10.1 Hz, 1H, –CH=CHC(CH_3_)_2_O–), 1.39 (s, 6H,–CH_3_); ^13^C NMR (100 MHz, DMSO-*d*_6_) *δ* 180.4 (C=O), 158.7, 157.4, 156.6, 155.9, 154.1, 130.1 (2 × C), 128.9, 122.3, 120.9, 115.0 (2 × C), 114.4, 105.3, 104.6, 94.5, 78.0, 27.8 (2 × C). The spectroscopic data correspond to reported values [38].

#### 3.2.12. 4-(5-Hydroxy-2,2-dimethyl-6-oxo-2*H*,6*H*-pyrano[3,2-g]chromen-7-yl)phenyl acetate (**25**)

Under a N_2_ atmosphere, to a solution of compound **6** (800 mg, 2.38 mmol) in pyridine (10 mL), Ac_2_O (0.31 mL, 3.57 mmol, 1.5 eq) was added.The mixture was stirred at rt for 1 h. The progress of the reaction was monitored by TLC. After completion of the reaction, the reaction mixture was quenched by dilution with 50 mL of H_2_O and extracted with CH_2_Cl_2._ The organic layer dried over anhydrous Na_2_SO_4_, and the solvent was evaporated in vaccum. The obtained residue was purified on a flash chromatography silica gel (PE/EtOAc, 25:1) to obtain the product **25** (756 mg, 84%) as a yellow solid. ^1^H NMR (400 MHz, CDCl_3_) *δ* 13.07 (s, 1H, –OH), 7.84 (s, 1H, –OCH=C–), 7.54 (d, *J* = 8.5 Hz, 2H, –Ph), 7.17 (d, *J* = 8.5 Hz, 2H, –Ph), 6.72 (d, *J* = 10.1 Hz, 1H, –CH=CHC(CH_3_)_2_O–), 6.33 (s, 1H, –Ph), 5.72 (d, *J* = 10.1 Hz, 1H, –CH=CHC(CH_3_)_2_O–), 2.32 (s, 3H, –CH_3_), 1.47 (s, 6H, –CH_3_); ^13^C NMR (150 MHz, CDCl_3_) *δ* 180.6 (C=O), 169.5, 159.7, 157.33, 156.9, 153.1, 150.8, 130.1 (2 × C), 128.5, 128., 123.1, 121.9, 115.5 (2 × C), 106.1, 105.8, 95.0, 78.2, 28.4 (2 × C), 21.2. The spectroscopic data correspond to reported values [39].

#### 3.2.13. 4-(8,8-Dimethyl-5-((3-methylbut-2-en-1-yl)oxy)-4-oxo-4*H*,8*H*-pyrano[2,3-f]chromen-3-yl)phenyl acetate (**26**)

Under a N_2_ atmosphere, to a solution of **25** (720 mg, 1.92 mmol), DEAD (0.6 mL, 3.84 mmol, 2 eq) and PPh_3_ (1.00 g, 3.84 mmol, 2 eq) in dry THF (25 mL), was added a solution of compound **18** (332 mg, 3.84 mmol, 2 eq) in 4 mL dry THF. The resulting mixture was stirred for 4 h at rt. After the reaction was completed (TLC), the solvent was removed under reduced pressure, and the yellow viscous oil was dissolved in EtOAc and subjected to flash chromatography on silica gel (EtOAc/PE 1:50 to 1:20) to give **26** (703 mg, 82%) as a yellow solid. Compound **26** was used immediately, without further purification.

#### 3.2.14. 4-(5-Hydroxy-2,2-dimethyl-10-(3-methylbut-2-en-1-yl)-6-oxo-2*H*,6*H*-pyrano[3,2-g]chromen-7-yl)phenyl acetate (**27**)

A suspension of **26** (800 mg, 1.79 mmol) and Eu(fod)_3_ (160 mg, 0.18 mmol, 0.1 eq) in dry DCE (25 mL) was stirred at reflux for 1 h under a N_2_ atmosphere. The resulting orange oil was concentrated in vacuum and subjected to flash chromatography (EtOAc/PE 1:50 to 1:20) to furnish **27** (730 mg, 91%) as a light yellow solid. ^1^H NMR (400 MHz, CDCl_3_) *δ* 13.02 (s, 1H, –OH), 7.93 (s, 1H, –OCH=C–), 7.56 (d, *J* = 8.5 Hz, 2H, –Ph), 7.18 (d, *J* = 8.5 Hz, 2H, –Ph), 6.74 (d, *J* = 10.0 Hz, 1H, –CH=CHC(CH_3_)_2_O–), 5.63 (d, *J* = 10.0 Hz, 1H, –CH=CHC(CH_3_)_2_O–), 5.18 (t, *J* = 7.1 Hz, 1H, –CH=C(CH_3_)_2_), 3.40 (d, *J* = 7.1 Hz, 2H, –CH_2_–), 2.33 (s, 3H, –Ac), 1.82 (s, 3H, –CH_3_), 1.69 (s, 3H, –CH_3_), 1.47 (s, 6H, –CH_3_); ^13^C NMR (100 MHz, CDCl_3_) *δ* 180.9 (C=O), 169.5, 157.1, 154.9, 154.6, 153.1, 150.7, 131.8, 130.1 (2 × C), 128.7, 128.1, 122.7, 121.9, 121.8 (2 × C), 115.8, 107.6, 105.9, 105.6, 77.9, 28.2 (2 × C), 25.8, 21.3, 21.2, 17.9. The spectroscopic data correspond to reported values [40].

#### 3.2.15. 5-Hydroxy-7-(4-hydroxyphenyl)-2,2-dimethyl-10-(3-methylbut-2-en-1-yl)-2*H*,6*H*-pyrano[3,2-g]chromen-6-one (**1**)

Compound **27** (400 mg, 0.89 mmol) was dissolved in 10 mL of EtOH and 0.17 mL of a 60% KOH aqueous solution (1.78 mmol). After stirring for 30 min, the mixture was acidified with 3 mol/L HCl to pH 6 and extracted with EtOAc. The organic phase was washed with brine, dried over Na_2_SO_4_, and evaporated. The residue was submitted to flash column chromatography (silica gel, EtOAc/PE 1:50 to 1:20) to afford target **1** (316 mg, 87%) as a yellow solid. ^1^H NMR (400 MHz, CDCl_3_) *δ* 13.03 (s, 1H, –OH), 7.89 (s, 1H, –OCH=C–), 7.33 (d, *J* = 7.2 Hz, 2H, –Ph), 6.81 (d, *J* = 7.2 Hz, 2H, –Ph), 6.74 (d, *J* = 10.0 Hz, 1H, –CH=CHC(CH_3_)_2_O–), 5.59 (d, *J* = 10.0 Hz, 1H, –CH=CHC(CH_3_)_2_O–), 5.24 (t, *J* = 7.2 Hz, 1H, –CH=C(CH_3_)_2_), 3.35 (d, *J* = 7.2 Hz, 2H, –CH_2_–), 1.81 (s, 3H, –CH_3_), 1.68 (s, 3H, –CH_3_), 1.48 (s, 6H, –CH_3_); ^13^C NMR (100 MHz, CDCl_3_) *δ* 180.4 (C=O), 155.9, 155.0, 153.7 (2 × C), 151.7, 130.7, 129.2 (2 × C), 127.0, 122.3, 121.7, 120.8, 114.7, 114.7 (2 × C), 106.5, 104.8, 104.4, 76.8, 27.1 (2 × C), 24.7, 20.2, 16.8. HRMS (ESI) calculated for C_25_H_25_O_5_^+^ [M + H]^+^ 405.1697, found 405.1697.

#### 3.2.16. 5-Hydroxy-3-((4-hydroxyphenyl)-8,8dimethyl-4*H*,8*H*-pyrano[2,3-f]chromen-4-one (**7**)

The method was identical to that described for the preparation of **6**. Compound **7** (1.75 g, 70%) was a light yellow solid. ^1^H NMR (400 MHz, DMSO-*d*_6_) *δ* 13.07 (s, 1H, –OH), 9.64 (s, 1H, –OH), 8.38 (s, 1H, –OCH=C–), 7.37 (d, *J* = 8.6 Hz, 2H, –Ph), 6.82 (d, *J* = 8.6 Hz, 2H, –Ph), 6.64 (d, *J* = 10.0 Hz, 1H, –CH=CHC(CH_3_)_2_O–), 6.23 (s, 1H, –Ph), 5.75 (d, *J* = 10.0 Hz, 1H, –CH=CHC(CH_3_)_2_O–), 1.42 (s, 6H, –CH_3_); ^13^C NMR (150 MHz, DMSO-*d*_6_) *δ* 181.1 (C=O), 162.0, 159.3, 158.1, 154.6, 152.1, 130.8 (2 × C), 128.7, 123.1, 121.5, 115.6 (2 × C), 114.4, 105.9, 101.2, 99.9, 78.7, 28.3 (2 × C). The spectroscopic data correspond to reported values [41].

#### 3.2.17. 3-(4-(Benzyloxy)phenyl)-5-hydroxy-7-((2-methylbut-3-yn-2-yl)oxy)-4*H*-chromen-4-one (**28**)

The method was identical to that described for the preparation of **25**. Compound **28** (744 mg, 82%) was a white solid. ^1^H NMR (400 MHz, CDCl_3_) *δ* 12.84 (s, 1H, –OH), 7.91 (s, 1H, –OCH=C–), 7.56–7.55 (m, 2H, –Ph), 7.17 (d, *J* = 8.6 Hz, 2H, –Ph), 6.68 (d, *J* = 10.0 Hz, 1H, –CH=CHC(CH_3_)_2_O–), 6.29 (s, 1H, –Ph), 5.59 (d, *J* = 10.0 Hz, 1H, –CH=CHC(CH_3_)_2_O–), 2.32 (s, 3H,–Ac), 1.47 (s, 6H, –CH_3_); ^13^C NMR (150 MHz, CDCl_3_) *δ* 180.7 (C=O), 169.6, 162.3, 159.8, 153.0, 152.2, 150.9, 130.1 (2 × C), 128.5, 127.7, 123.3, 121.9, 114.6 (2 × C), 106.1, 101.3, 100.6, 78.3, 28.3 (2 × C), 21.3. HRMS (ESI) calculated for C_22_H_19_O_6_^+^ [M + H]^+^ 379.1176, found 379.1176.

#### 3.2.18. 4-(8,8-Dimethyl-5-((2-methylbut-3-en-2-yl)oxy)-4-oxo-4*H*,8*H*-pyrano[2,3-f]chromen-3-yl)phenyl acetate (**30**)

To a solution of 2-methylbut-3-en-2-ol (3.5 mL, 33.48 mmol, 1.0 eq) in THF (60 mL), *n*-BuLi was added in hexanes (2.5 M, 13.2 mL, 33.48 mmol, 1.0 eq) at 0 °C over the course of 10 min. The clear solution was stirred at 0 °C for 20 min and then added *di*-*tert*-butyl dicarbonate (7.32 g, 33.48 mmol, 1.0 eq). The clear solution was allowed to heat to 23 °C and further stirred for 4 h. The solvent was poured into ice-cold water, quenched with a saturated aqueous NaHCO_3_ solution, and extracted with EtOAc. The organic phase was separated, washed with sat. aq. NaCl (150 mL), dried over Na_2_SO_4,_ and concentrated under reduced pressure to give **29** as a clear, pale, light yellow liquid (6.03 g, 96%), which was used without further purification.

To a stirred suspension of **28** (200 mg, 0.53 mmol) and pulverized 4Å molecular sieves (100 mg) in degassed THF (5 mL), **29** (500 mg, 2.65 mmol) was added at room temperature. After cooling to −20 °C, Pd(PPh_3_)_4_ (61 mg, 0.05 mmol) was added portionwise to the mixture. After stirring for 21 h, the reaction mixture was filtered through a pad of Celite and the filtrate was concentrated in a vacuum. The residue was purified by silica gel column chromatography (PE/EtOAc = 30:1) to give **30** (178 mg, 78%) as a colorless oil, which was used without further purification.

#### 3.2.19. 4-(5-Hydroxy-8,8-dimethyl-6-(3-methylbut-2-en-1-yl)-4-oxo-4*H*,8*H*-pyrano[2,3-f]chromen-3-yl)phenyl acetate (**31**)

The method was identical to that described for the preparation of **27**. Compound **31** was a yellow solid (420 mg, 75%). ^1^H NMR (400 MHz, CDCl_3_) *δ* 13.07 (s, 1H, –OH), 7.89 (s, 1H, –OCH=C–), 7.54 (d, *J* = 8.3 Hz, 2H, –Ph), 7.17 (d, *J* = 8.3 Hz, 2H, –Ph), 6.69 (d, *J* = 10.0 Hz, 1H, –CH=CHC(CH_3_)_2_O–), 5.59 (d, *J* = 10.0 Hz, 1H, –CH=CHC(CH_3_)_2_O–), 5.23 (t, *J* = 6.6 Hz, 1H, –CH=C(CH_3_)_2_), 3.35 (d, *J* = 6.6 Hz, 2H, –CH_2_–), 2.33 (s, 3H, –Ac), 1.81 (s, 3H, –CH_3_), 1.68 (s, 3H, –CH_3_), 1.48 (s, 6H, –CH_3_); ^13^C NMR (100 MHz, CDCl_3_) *δ* 180.6 (C=O), 169.5, 159.4, 157.3, 152.7, 150.7, 150.5, 131.7, 130.1 (2 × C), 128.7, 127.3, 122.9, 121.9,121.8 (2 × C), 114.9, 113.0, 105.6, 100.8, 77.9, 28.2 (2 × C), 25.8, 21.3, 21.2, 17.9. HRMS (ESI) calculated for C_27_H_27_O_6_^+^ [M + H]^+^ 447.1802, found 447.1803.

#### 3.2.20. 5-Hydroxy-3-(4-hydroxyphenyl)-8,8-dimethyl-6-(3-methylbut-2-en-1-yl)-4*H*,8*H*-pyrano[2,3-*f*]chromen-4-one (**2**)

The method was identical to that described for the preparation of **1**. Compound **2** was a yellow solid (320 mg, 71%). ^1^H NMR (400 MHz, CDCl_3_) *δ* 13.07 (s, 1H, –OH), 7.85 (s, 1H, –OCH=C–), 7.31 (d, *J* = 7.3 Hz, 2H, –Ph), 6.81 (d, *J* = 7.3 Hz, 2H, –Ph), 6.70 (d, *J* = 9.9 Hz, 1H, –CH=CHC(CH_3_)_2_O–), 5.59 (d, *J* = 9.9 Hz, 1H, –CH=CHC(CH_3_)_2_O–), 5.24 (t, *J* = 7.3 Hz, 1H, –CH=C(CH_3_)_2_), 3.35 (d, *J* = 7.3 Hz, 2H, –CH_2_–), 1.81 (s, 3H, –CH_3_), 1.68 (s, 3H, –CH_3_), 1.48 (s, 6H, –CH_3_); ^13^C NMR (100 MHz, CDCl_3_) *δ* 180.1 (C=O), 158.2, 156.2, 155.0, 151.3, 149.5, 130.6, 129.3 (2 × C), 126.1, 122.5, 121.8, 120.8, 114.7 (2 × C), 113.9, 111.8, 104.5, 99.7, 76.8, 27.1 (2 × C), 24.7, 20.2, 16.9. HRMS (ESI) calculated for C_25_H_25_O_5_^+^ [M + H]^+^ 405.1697, found 405.1696.

#### 3.2.21. MICs Tests

*S. aureus* ATCC29213, *E. faecalis* ATCC29212, MRSA ATCC33591, and *E. coli* ATCC 25922 were selected to evaluate the target compounds **1**–**3**. The bacterial strains were purchased from the American Type Culture Collection (ATCC, Manassas, VA, USA) and kept in our laboratory. The MICs were evaluated according to the guidelines of the Clinical and Laboratory Standards Institute (CLSI). The experiment was performed as reported in the literature [32,33].

#### 3.2.22. Scanning Electron Microscopy (SEM) Characterization

A single colony of *S. aureus* ATCC29213 or *E. faecalis* ATCC29212 was added to LB broth (1.0 mL) and incubated in a shaker (200 rpm, 37 °C) for 12 h. Then the solution was diluted to a concentration of 1 × 10^9^ CFU/mL in PBS. After that, compound **2** (8 × MIC) was added, and the resulting mixture was incubated at 37 °C for 2 h. Subsequently, the bacterial cells were washed twice with PBS and added to 2.5% glutaraldehyde overnight at 4 °C. The bacterial cells were osmicated in 1% Osmium tetroxide for 1 h at 4 °C to further fix and stain them, then gradually eluted by an ascending graded series of EtOH, dried, and plated with gold at the critical point. Finally, the cells were visualized under SEM (Hitachi S-3400 N, Tokyo, Japan). The negative control was a bacterial suspension without compound **2** treatment [32,33].

## 4. Conclusions

In conclusion, the efficient syntheses of three natural isoflavones, scandenone (**1**), osajin (**2**), and 6,8-diprenylgenistein (**3**), were accomplished with overall yields of 10%, 6%, and 14%, respectively. Notably, natural isoflavone **3** was synthesized for the first time. The presently described synthesis of compounds **1** and **2** features a stereoselective construction of the pyran ring, and di-prenylation of key intermediate **15** is the key step to obtain isoflavone **3**. The antibacterial activities of the synthetic isoflavones **1**–**3** against *S. aureus* ATCC29213, *E. faecalis* ATCC29212, MRSA ATCC33591, and *E. coli* ATCC 25922 were assessed, and it was shown that all of the natural isoflavones displayed good antibacterial activity against Gram-positive bacteria. Furthermore, the SEM assay indicated that the bacterial cell membranes of Gram-positive bacteria could be disrupted by this kind of natural isoflavone. Taken together, these results demonstrate that natural prenylated isoflavones have tremendous potential to be developed as lead compounds for further optimization to address the problem of bacterial resistance.

## Figures and Tables

**Table 1:** Synthesis of Isoflavone **3**.

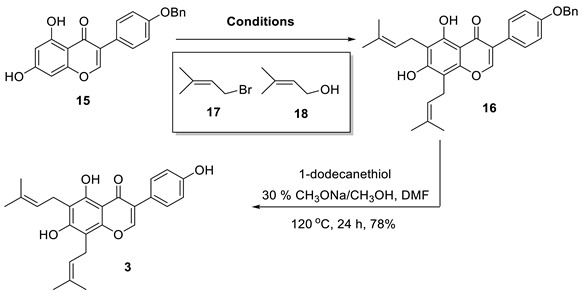		
Entry	Conditions	Yields of Isolated 16 (%)
1	compound **17**, KOH, H_2_O, 0 °C to rt	-
2	compound **17**, K_2_CO_3_, acetone, rt	7
3	compound **17**, DBU, THF, 50 °C	20
4	compound **18**, *p*-toluenesulfonic acid, CH_2_Cl_2_, rt	14
5	compound **18**, acidic alumina, DCE, 80 °C	72

**Table 2:** Optimization of Reaction Conditions for Compounds **19** and **20**.

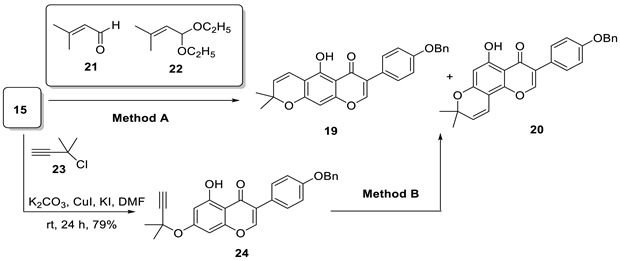				
Entry	Method	Conditions	19, Yield ^1^ (%)	20, Yield ^1^ (%)
1	Method A	**21**, Ca(OH)_2_, CH_3_OH, 0 °C to rt	27	22
2	Method A	**22**, 3-picoline, toluene, 110 °C	25	27
3	Method B	DMF, 130 °C	9	77
4	Method B	xylene, 130 °C	55	33
5	Method B	xylene, KOH, 130 °C	93	-
6	Method B	diethylaniline, 250 °C	41	40

^1^ Isolated yield.

**Table 3:** MICs of compounds **1**–**3**.

Compound	MIC (μg/mL)			
	*S. aureus* ATCC29213	*E. faecalis* ATCC29212	MRSA ATCC33591	*E. coli* ATCC25922
**1**	16	16	4	>128
**2**	2	8	2	>128
**3**	8	16	4	>128
Ampicillin	32	1	>128	4
Vancomycin	2	2	1	128

## Data Availability

Data are contained within the article and Supplementary Materials.

## Supplementary Materials

The following supporting information can be downloaded at: https://www.mdpi.com/article/10.3390/molecules29112574/s1. ^1^H NMR and ^13^C NMR spectra for the synthesized compounds and HRMS spectra for new compounds. Key crystal data for compound **1**, and LCMS reports for compounds **1**–**3** (PDF).
