# Supplementary material for: Total Syntheses and Antibacterial Studies of Natural Isoflavones: Scandenone, Osajin, and 6,8-Diprenylgenistein
Source: Molecules. 2024 May 30;29(11):2574. doi: 10.3390/molecules29112574 (PMC11173660; doi:10.3390/molecules29112574)
Supplement: Supplementary file 1 [file molecules-29-02574-s001.zip › molecules-3029922-supplementary.pdf]

Supporting Information

Total Syntheses and Antibacterial Studies of Natural Isoflavones: Scandenone, Osajin, and 6,8-Diprenylgenistein

Hongbo Dong 1,†, Yufei Che 1,†, Xingtong Zhu 2, Yi Zhong 1, Jiafu Lin 1, Jian Wang 1, Weihong Du 1,* and Tao Song 1,*

[1] Anti-infective Agent Creation Engineering Research Centre of Sichuan Province, School of Pharmacy, Chengdu University, Chengdu 610106, China. donghongbo@cdu.edu.cn (H.D.); cheyufei2023@163.com (Y.C.); zhongyi@stu.cdu.edu.cn (Y.Z.); linjiafu@cdu.edu.cn (J.L.); wangjian@cdu.edu.cn (J.W.)

[2] School of Food Science and Biological engineering, Zhejiang Gongshang University, Hangzhou 310018, China.; zxtttt2000@163.com

* Correspondence: duweihong@cdu.edu.cn (W.D.); songtao@cdu.edu.cn (T.S.)

† These authors contributed equally to this work.

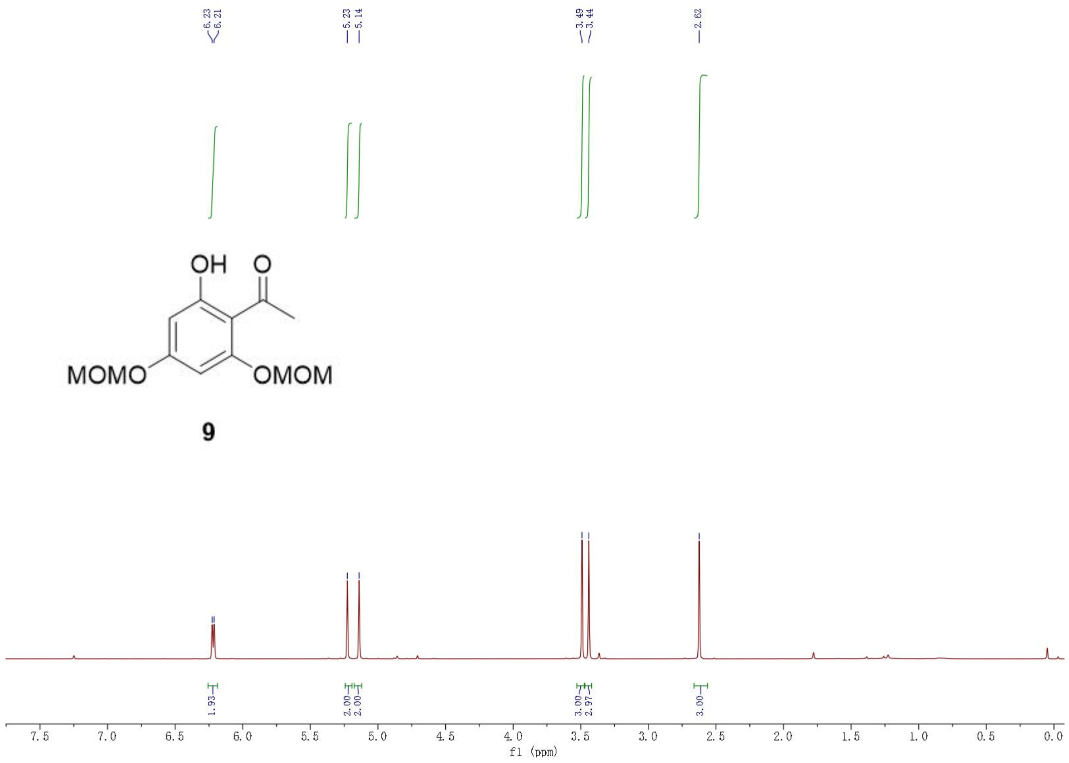

Figure S1 ^{1}H NMR (600 MHz, $CDCl_3$) of compound **9**.

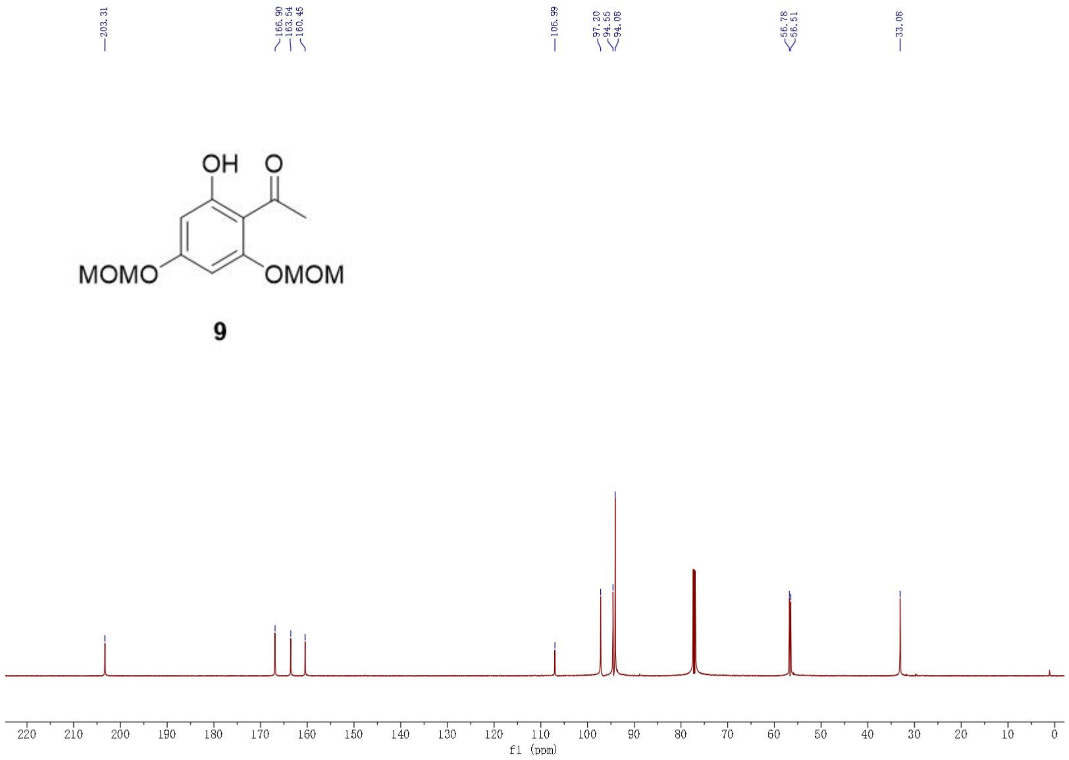

Figure S2 ^{13}C NMR (150 MHz, $CDCl_3$) of compound **9**.

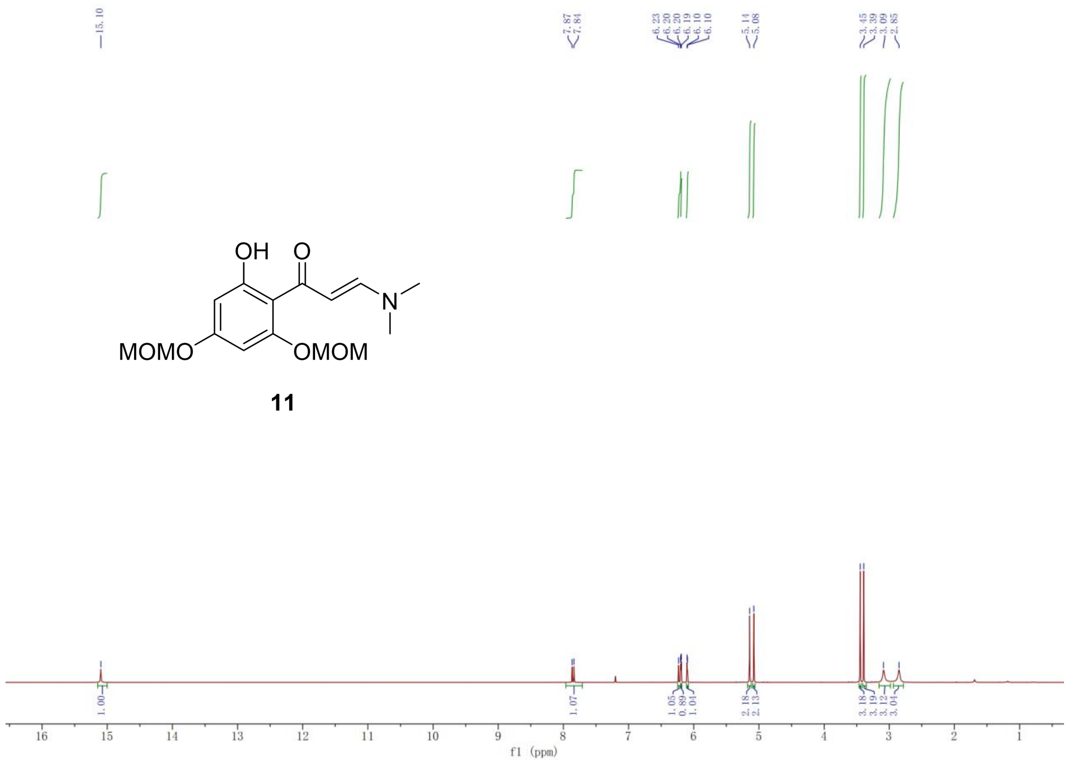

Figure S3 ^{1}H NMR (400 MHz, $CDCl_3$) of compound **11**.

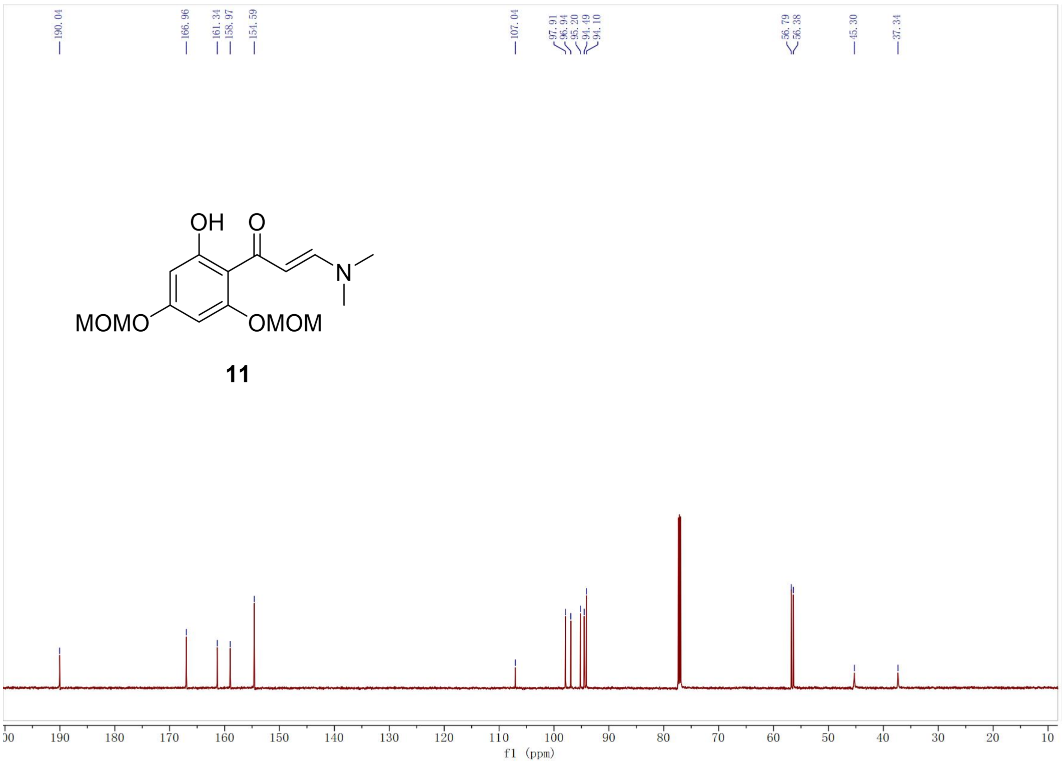

Figure S4 ^{13}C NMR (100 MHz, $CDCl_3$) of compound **11**.

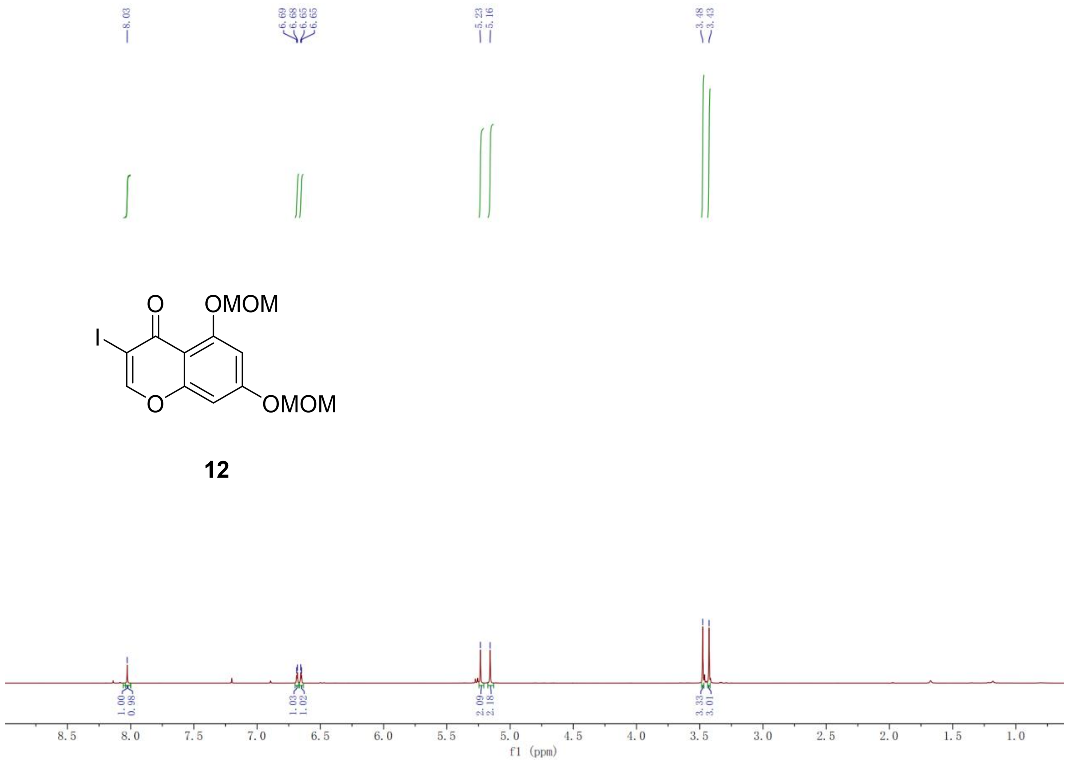

Figure S5 ^{1}H NMR (400 MHz, $CDCl_3$) of compound **12**.

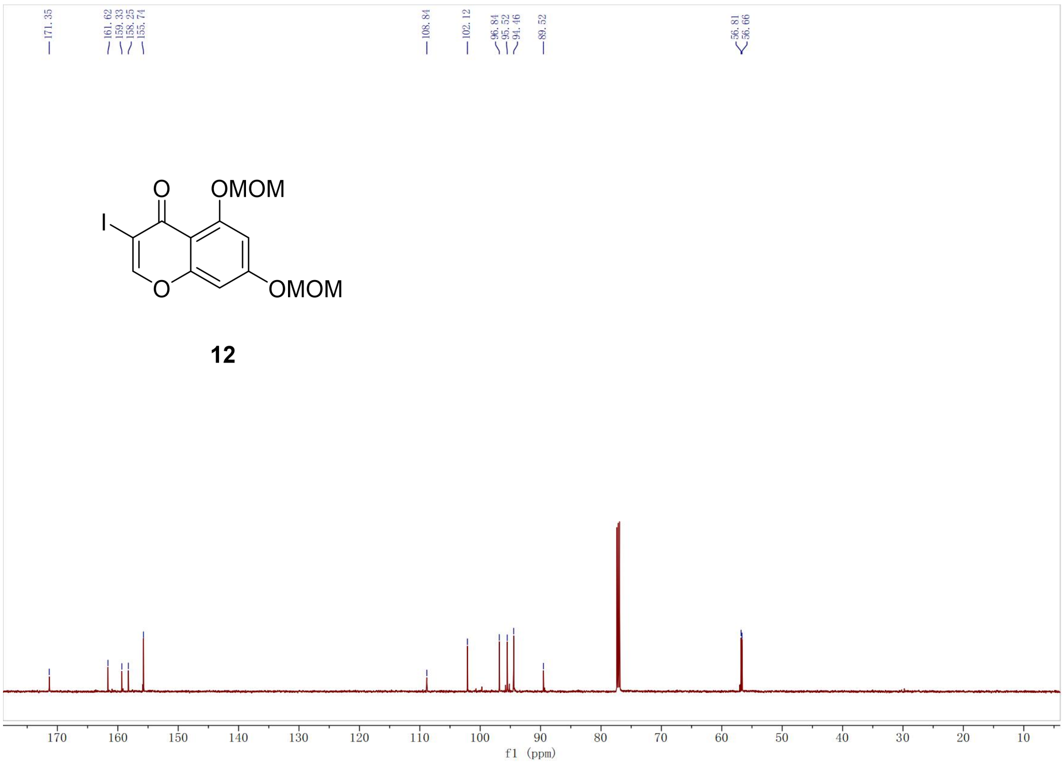

Figure S6 ^{13}C NMR(100 MHz, $CDCl_3$)of compound **12**.

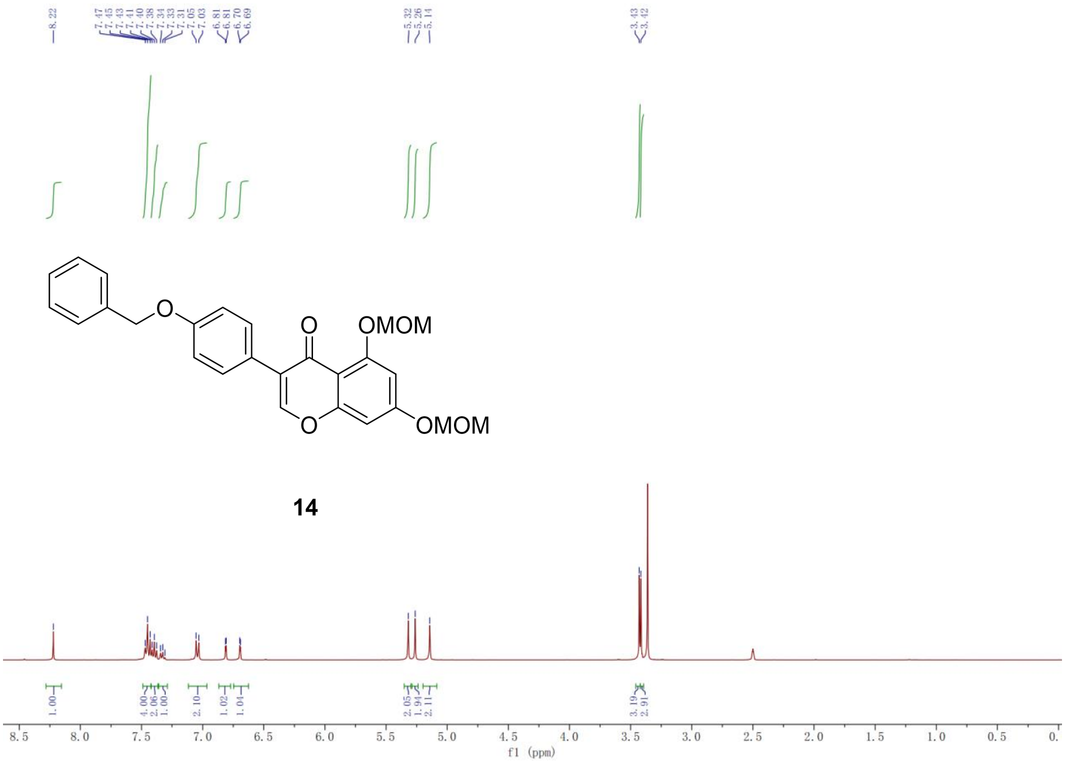

Figure S7 ^{1}H NMR (400 MHz, DMSO-d_6) of compound **14**.

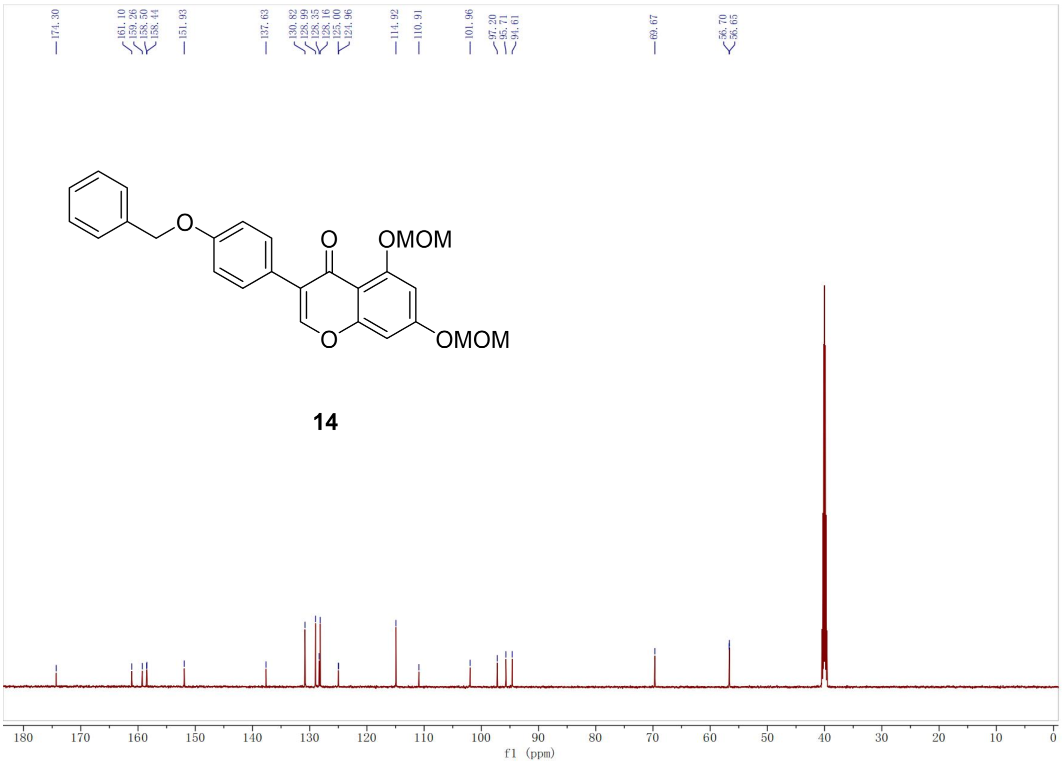

Figure S8 ^{13}C NMR(100 MHz, DMSO-d_6)of compound **14**.

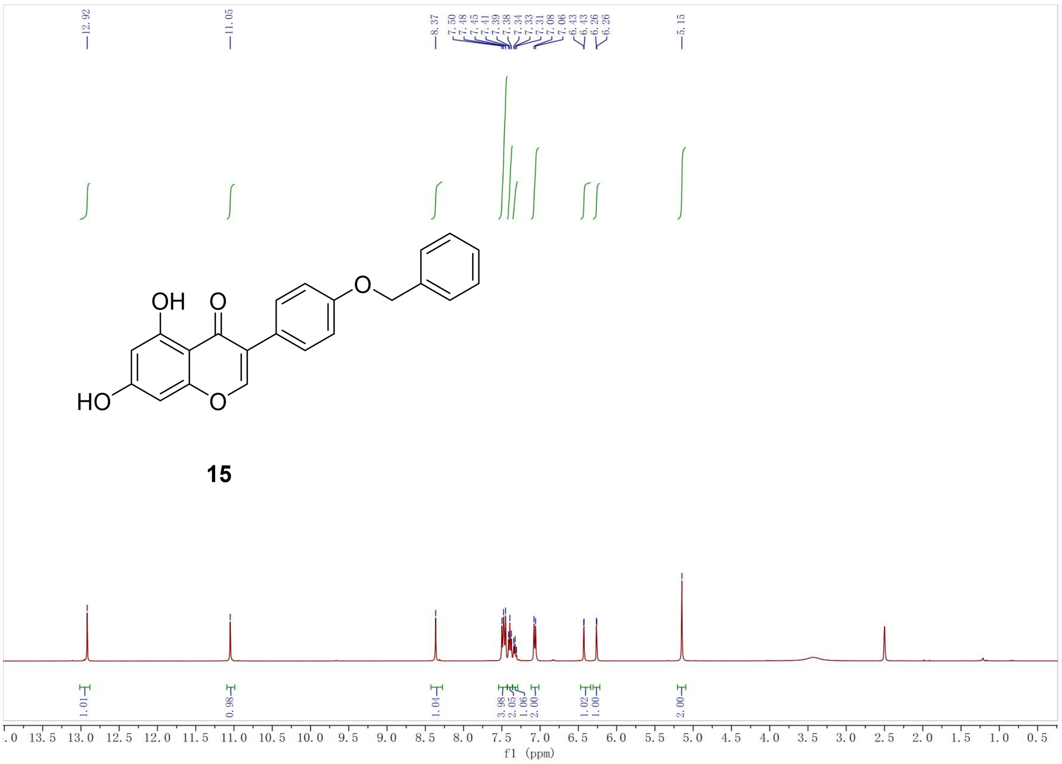

Figure S9 ^{1}H NMR (400 MHz, DMSO-d_6) of compound **15**.

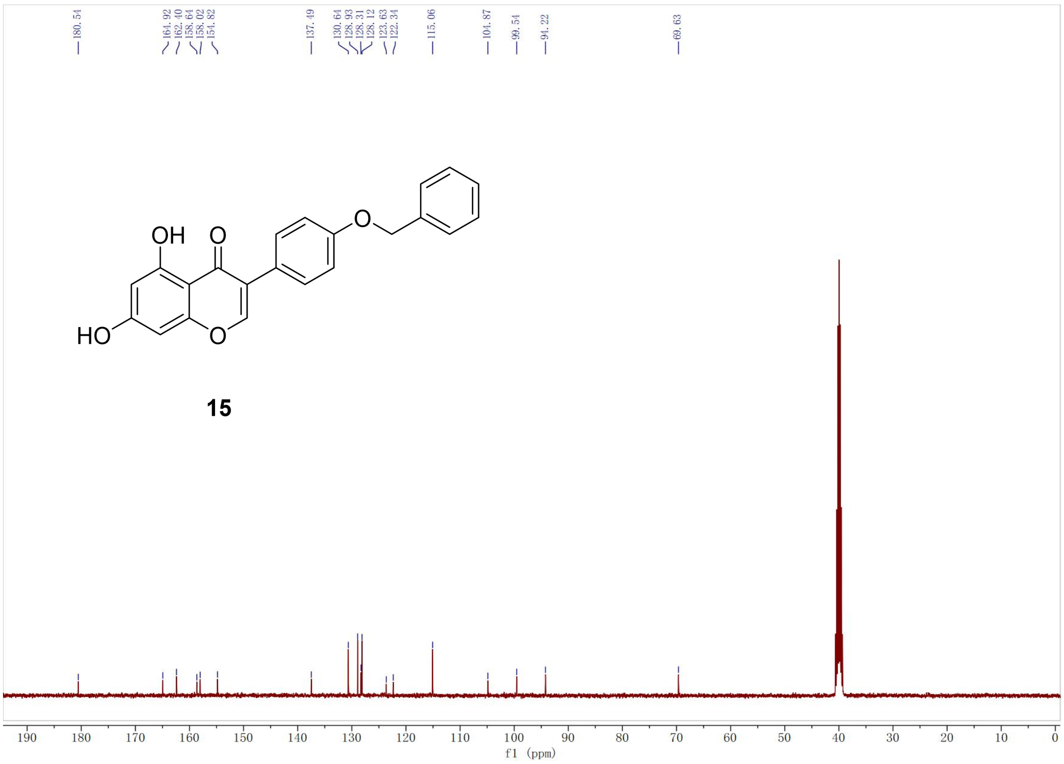

Figure S10 ^{13}C NMR(100 MHz, DMSO-d_6)of compound **15**.

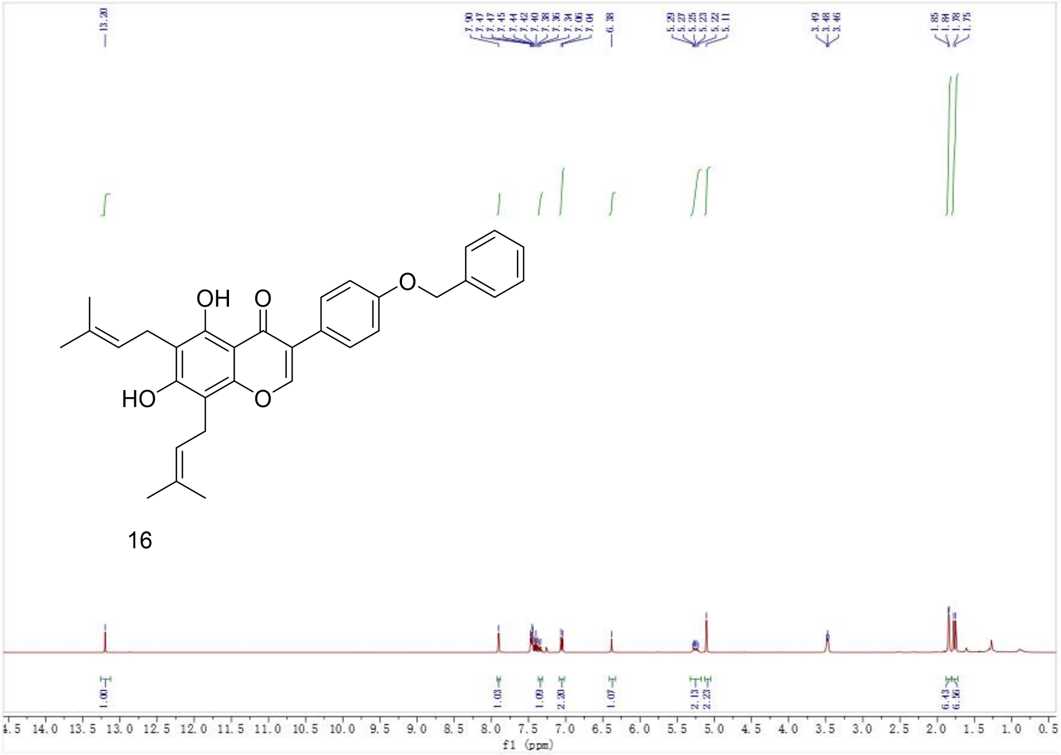

Figure S11 ^{1}H NMR (400 MHz, CDCl3) of compound **16**.

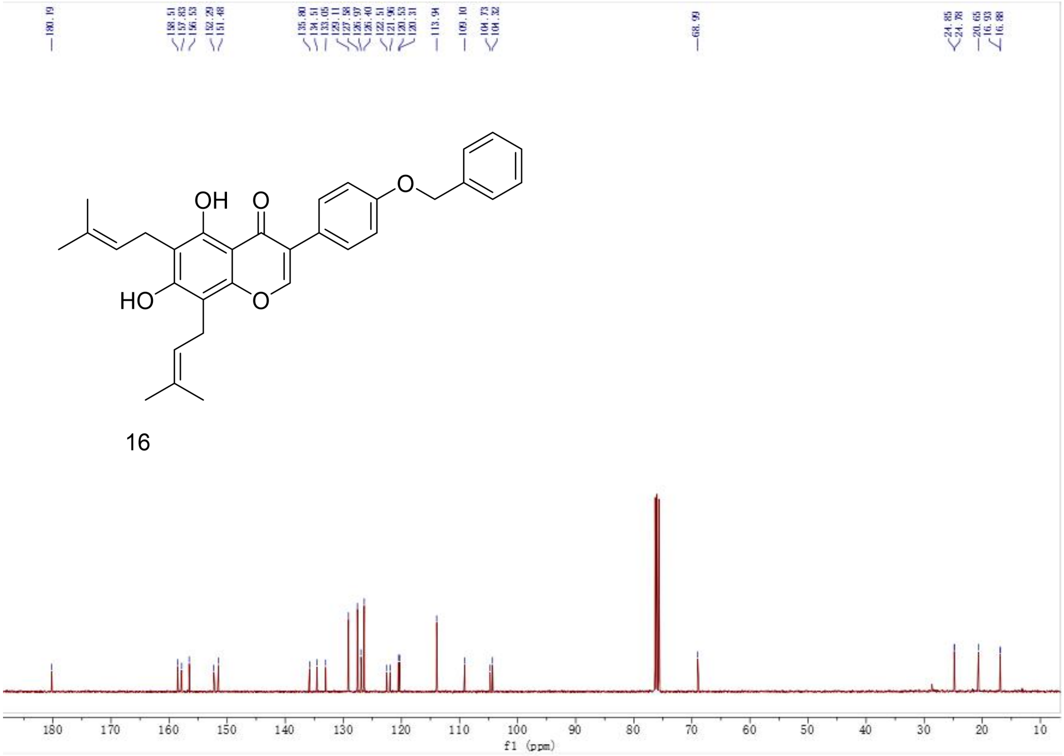

Figure S12 ^{13}C NMR(100 MHz, CDCl3) of compound **16**.

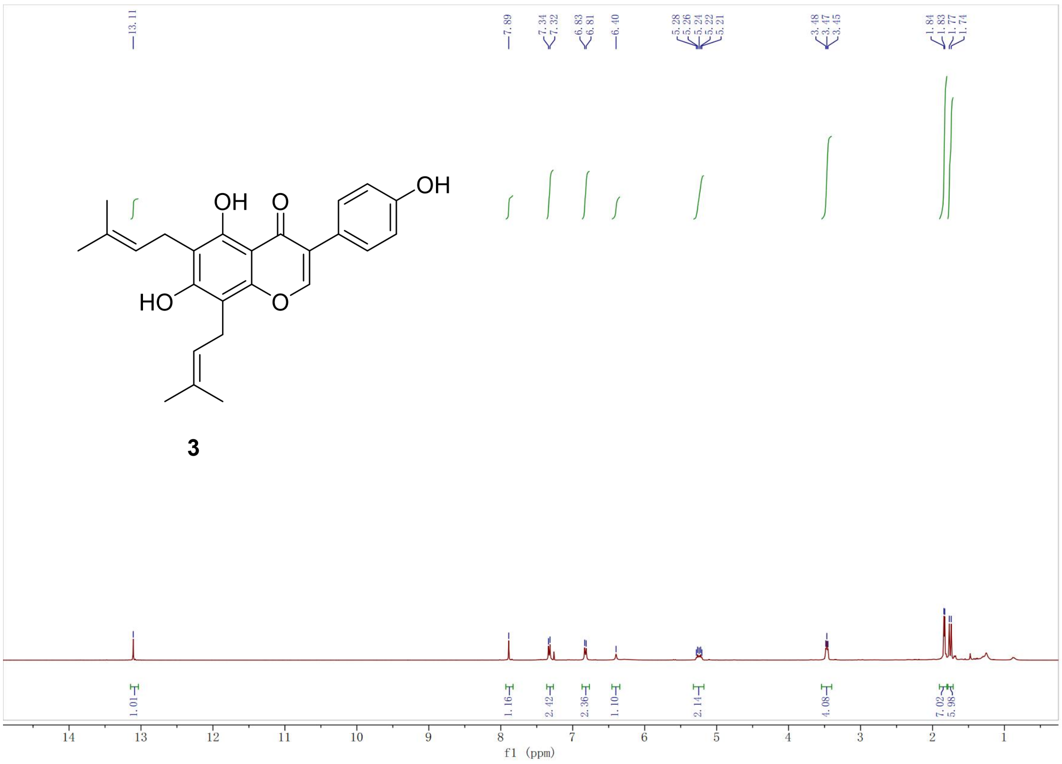

Figure S13 ^{1}H NMR (400 MHz, $CDCl_3$) of compound **3**.

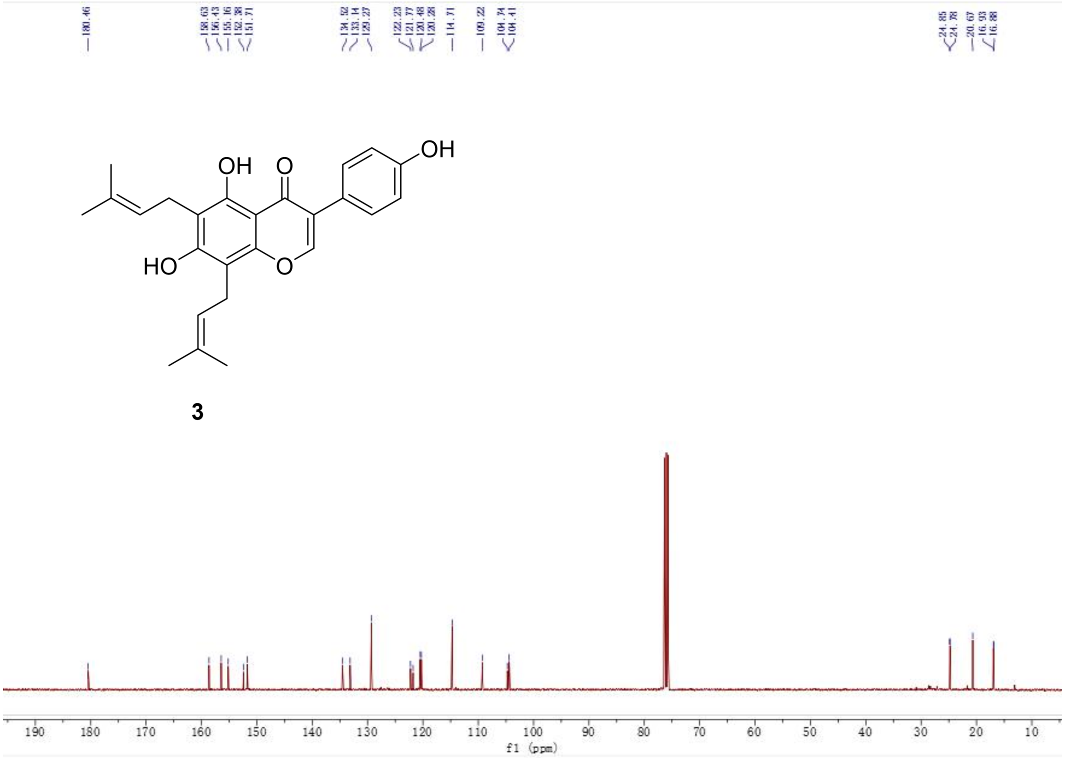

Figure S14 ^{13}C NMR(100 MHz, $CDCl_3$)of compound **3**.

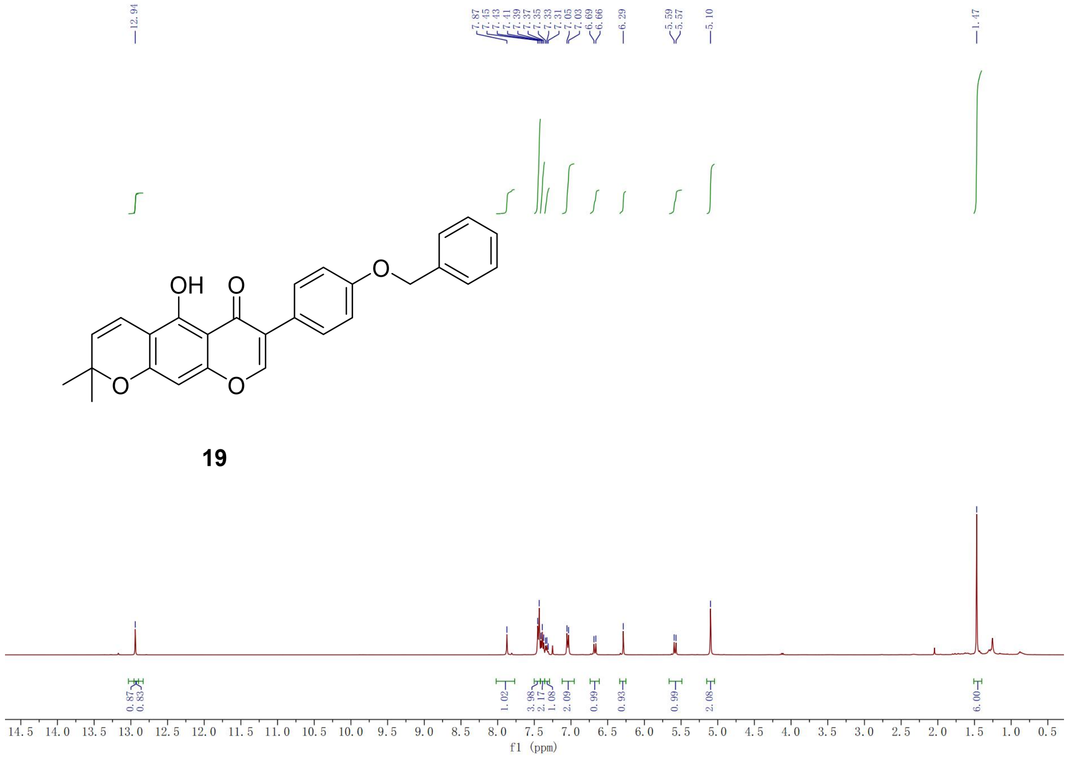

Figure S15 ^{1}H NMR (400 MHz, $CDCl_3$) of compound **19**.

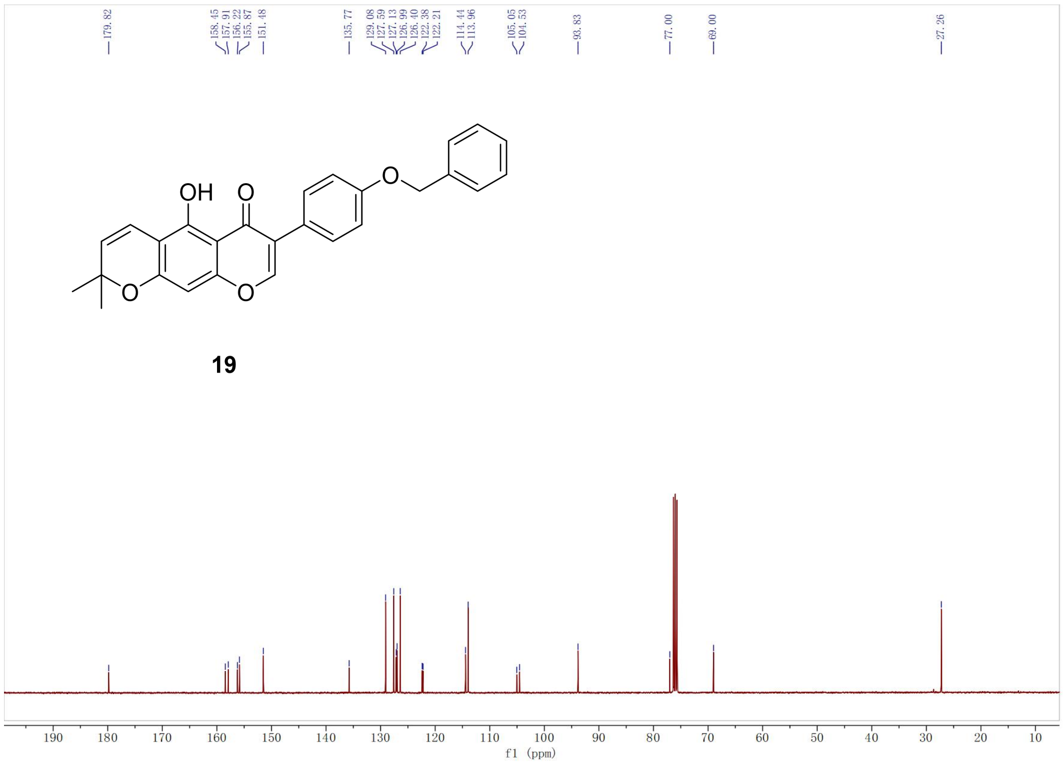

Figure S16 ^{13}C NMR(100 MHz, $CDCl_3$) of compound **19**.

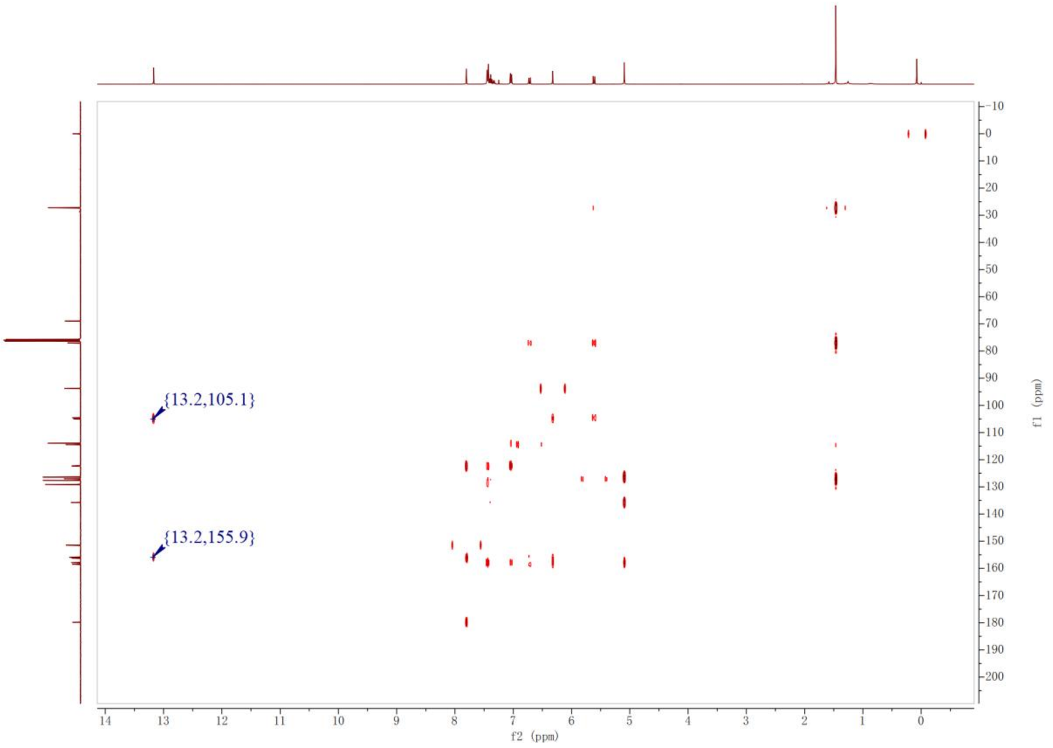

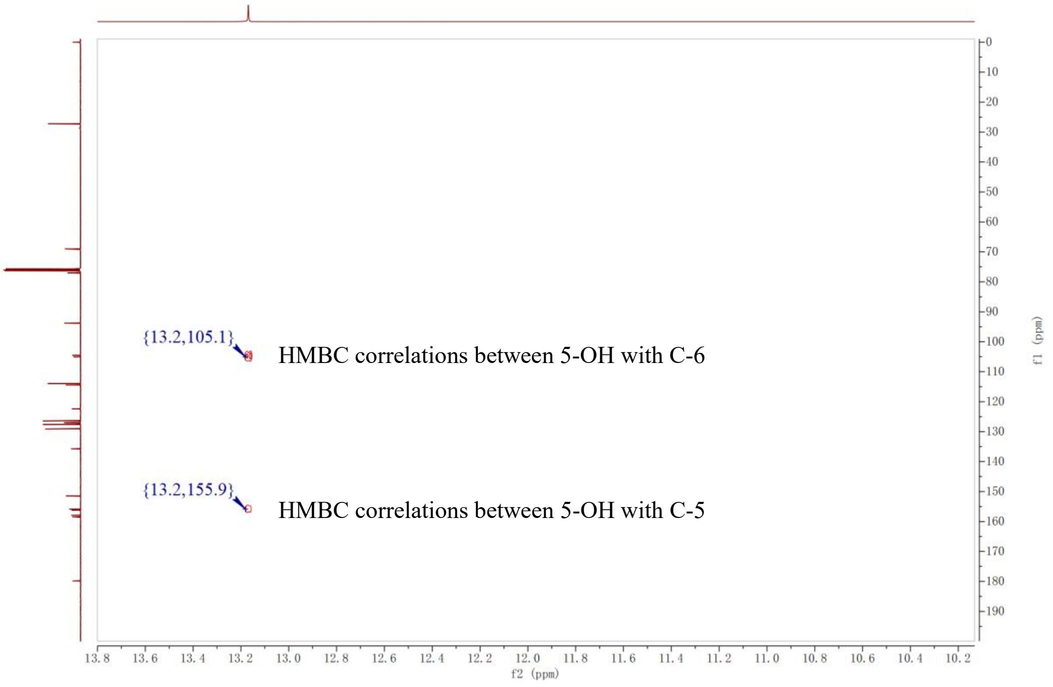

Figure S17 HMBC of compound **19**.

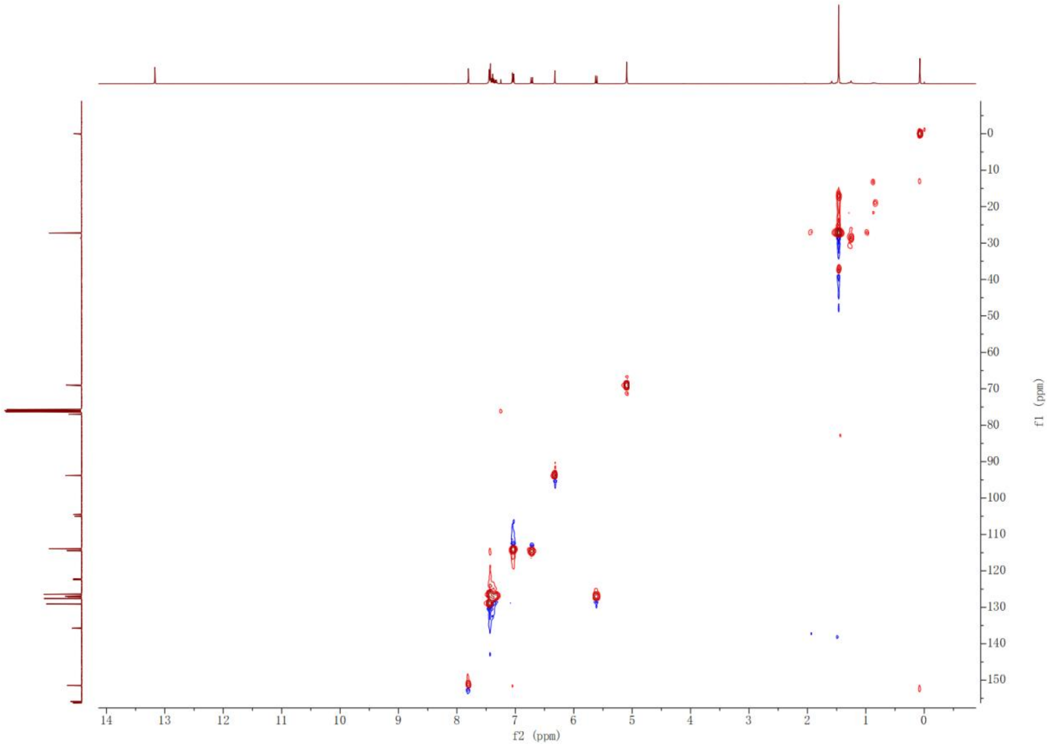

Figure S18 HSQC of compound **19**.

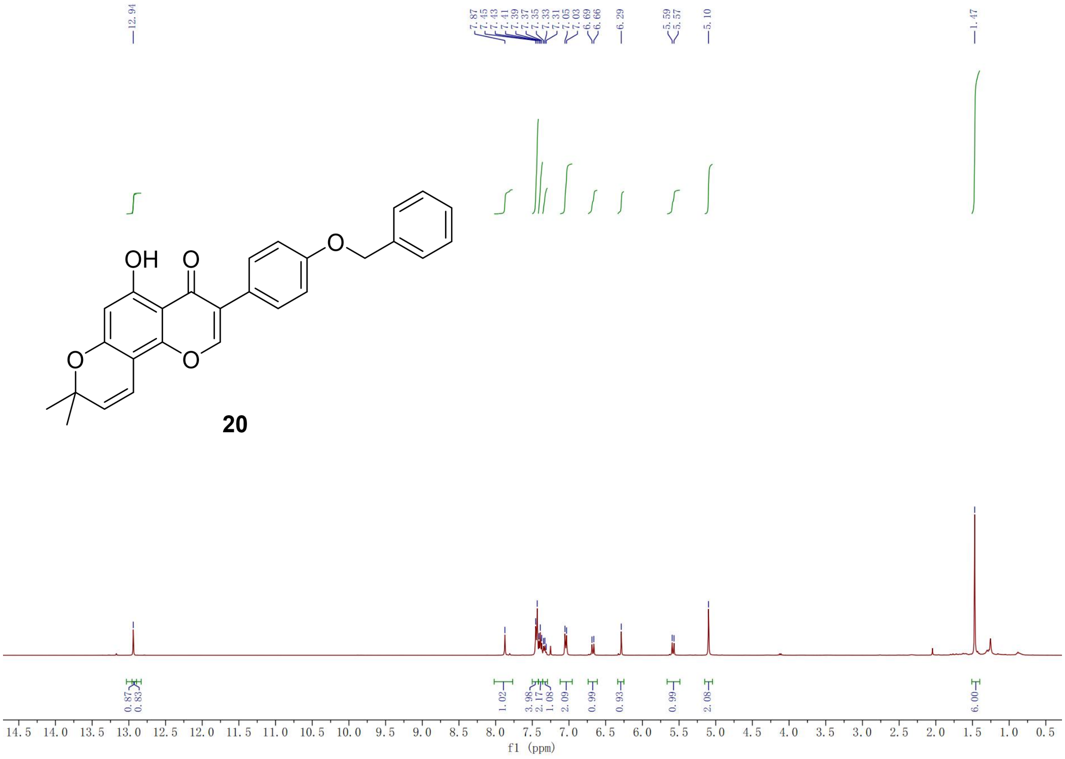

Figure S19 ^{1}H NMR (400 MHz, $CDCl_3$) of compound **20**.

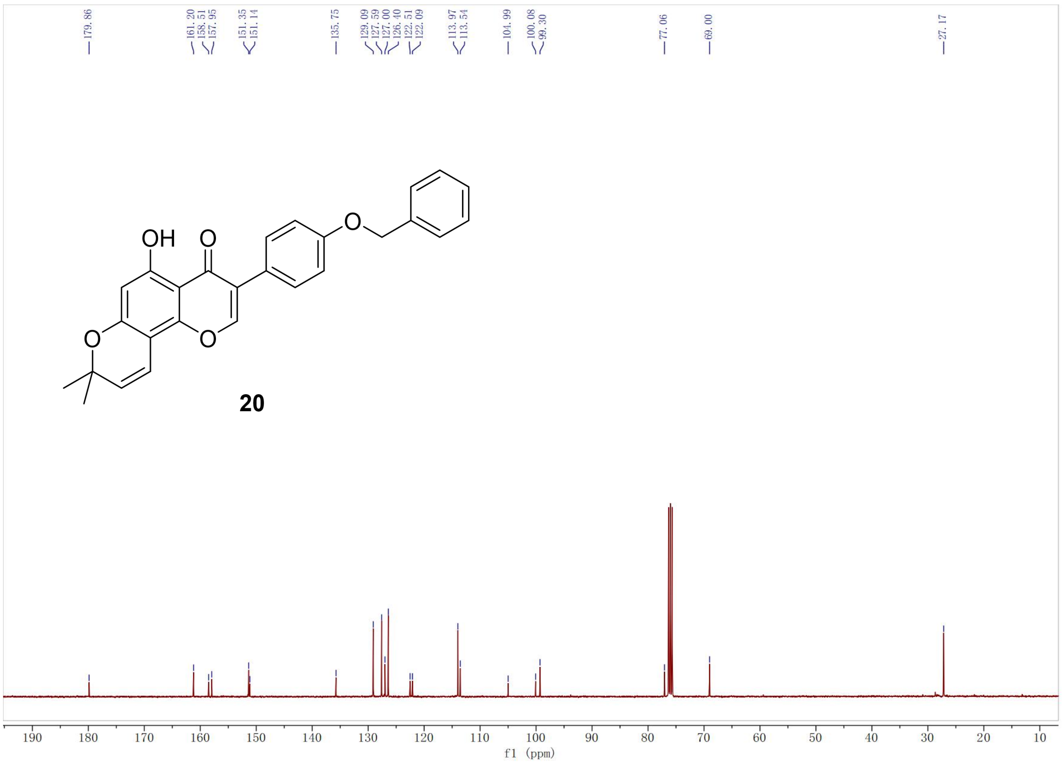

Figure S20 ^{13}C NMR(100 MHz, $CDCl_3$) of compound **20**.

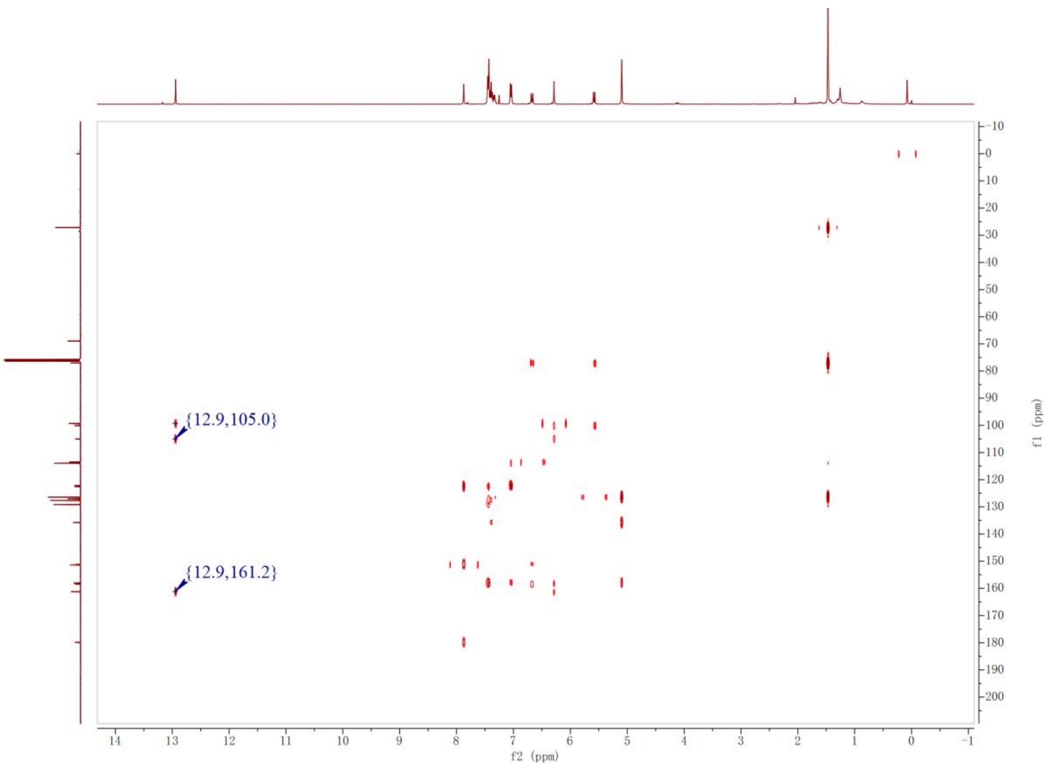

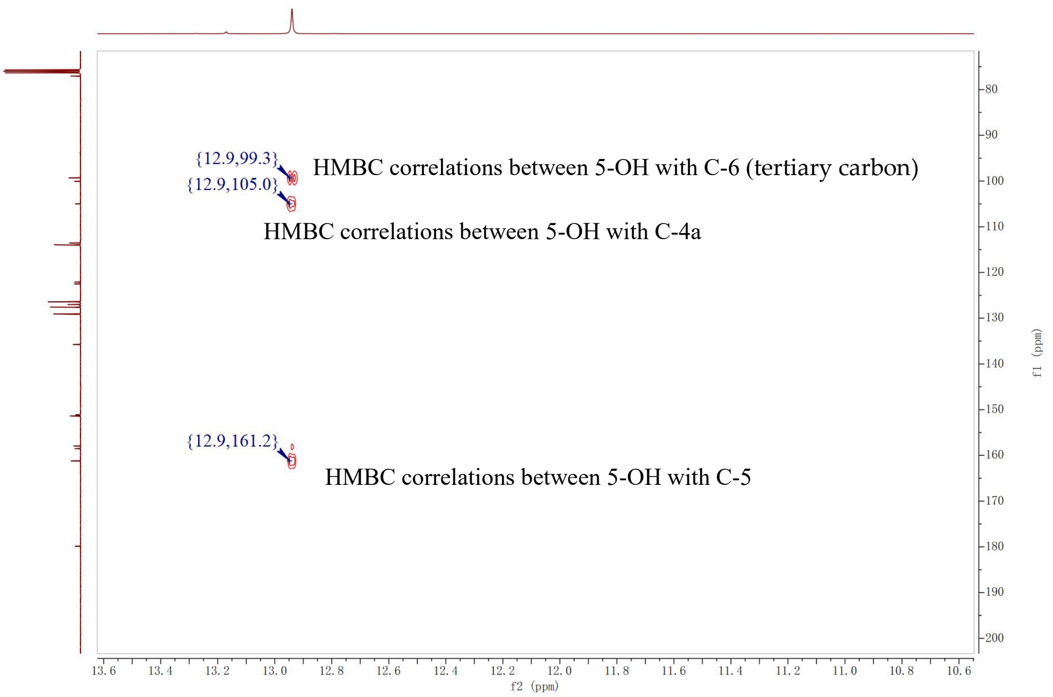

Figure S21 HMBC of compound **20**.

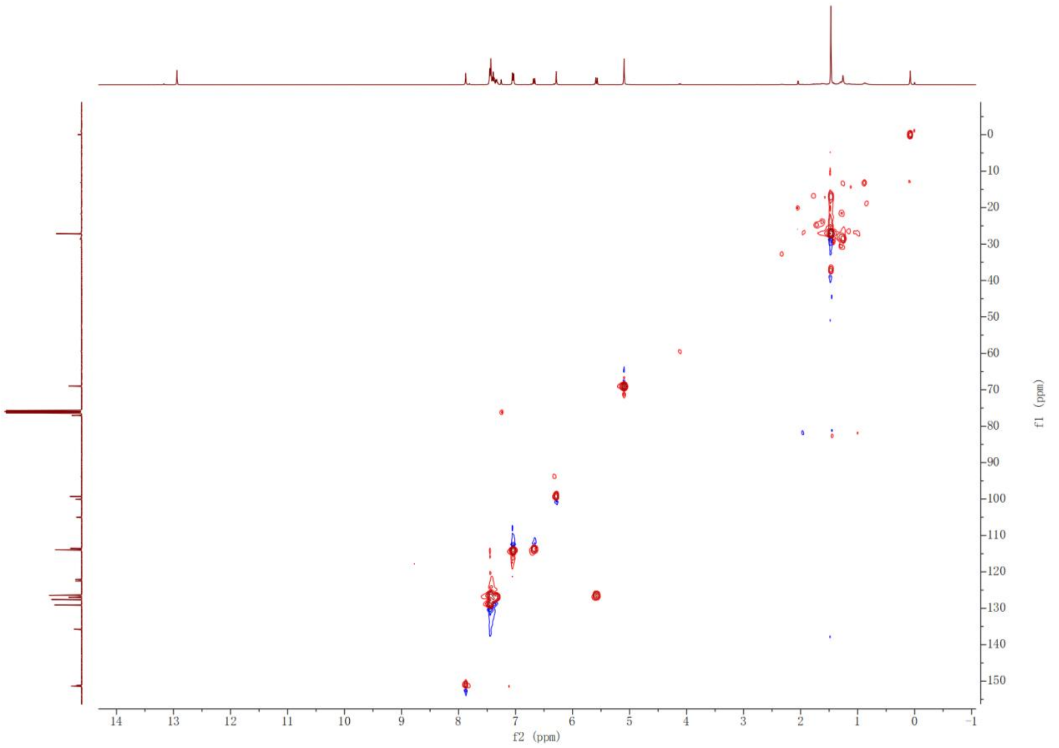

Figure S22 HSQC of compound **20**.

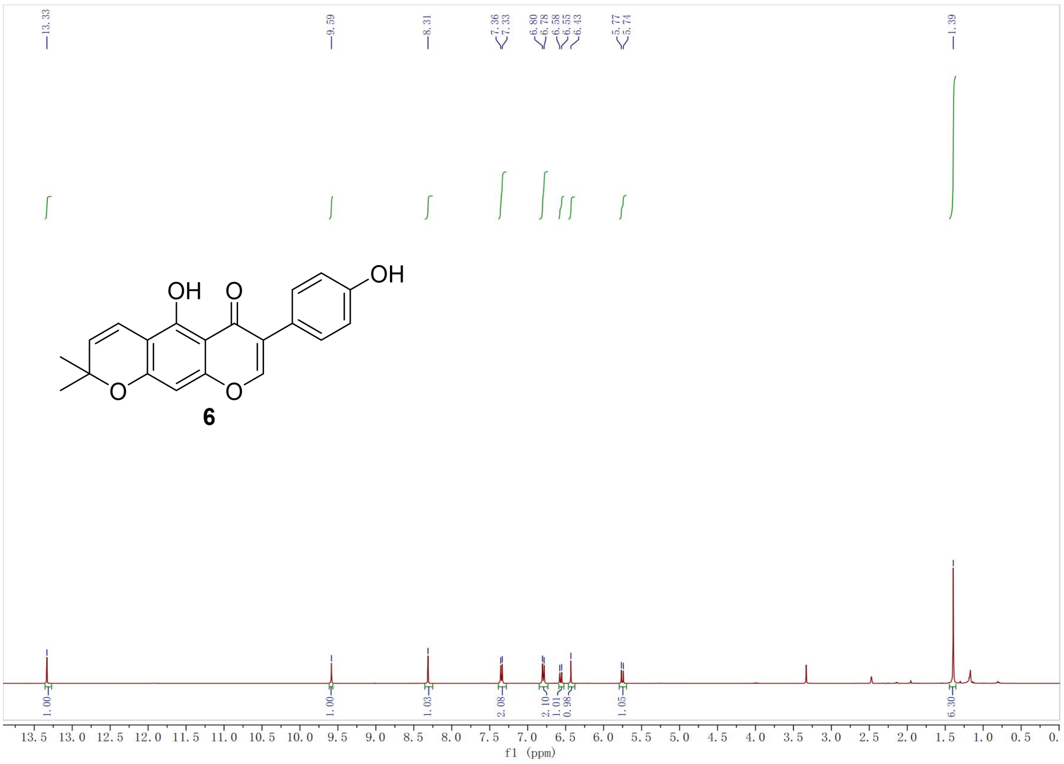

Figure S23 ^{1}H NMR (400 MHz, DMSO-d_6) of compound **6**.

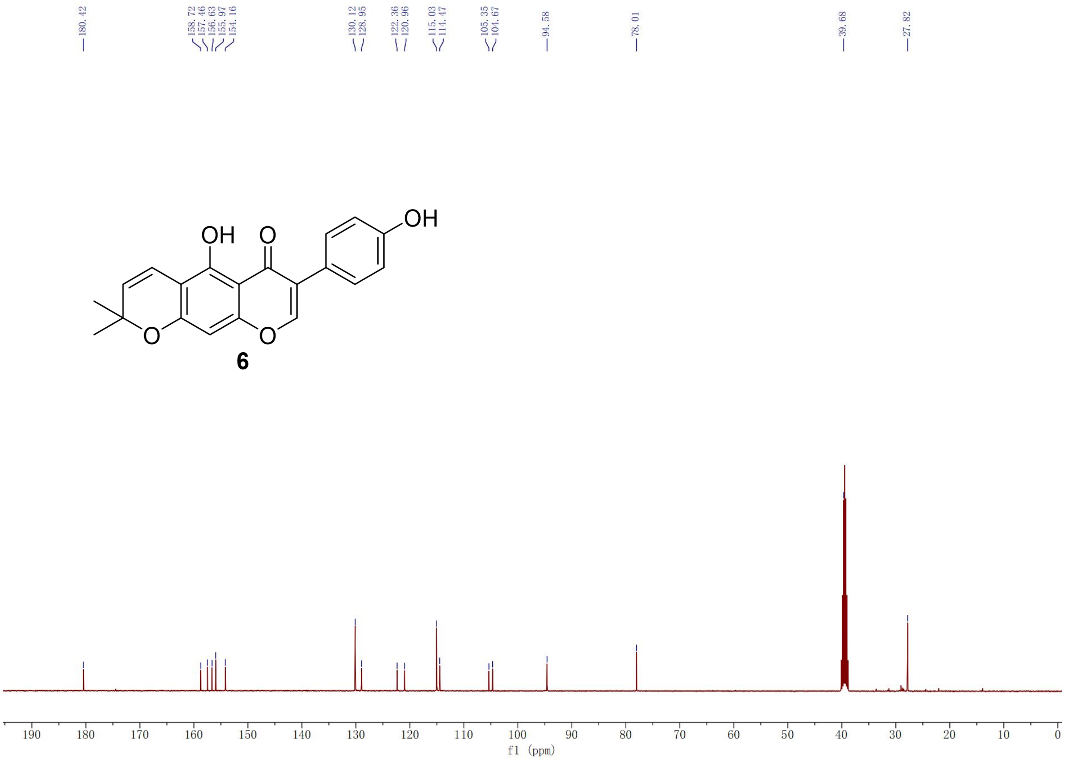

Figure S24 ^{13}C NMR (150 MHz, DMSO-d_6) of compound **6**.

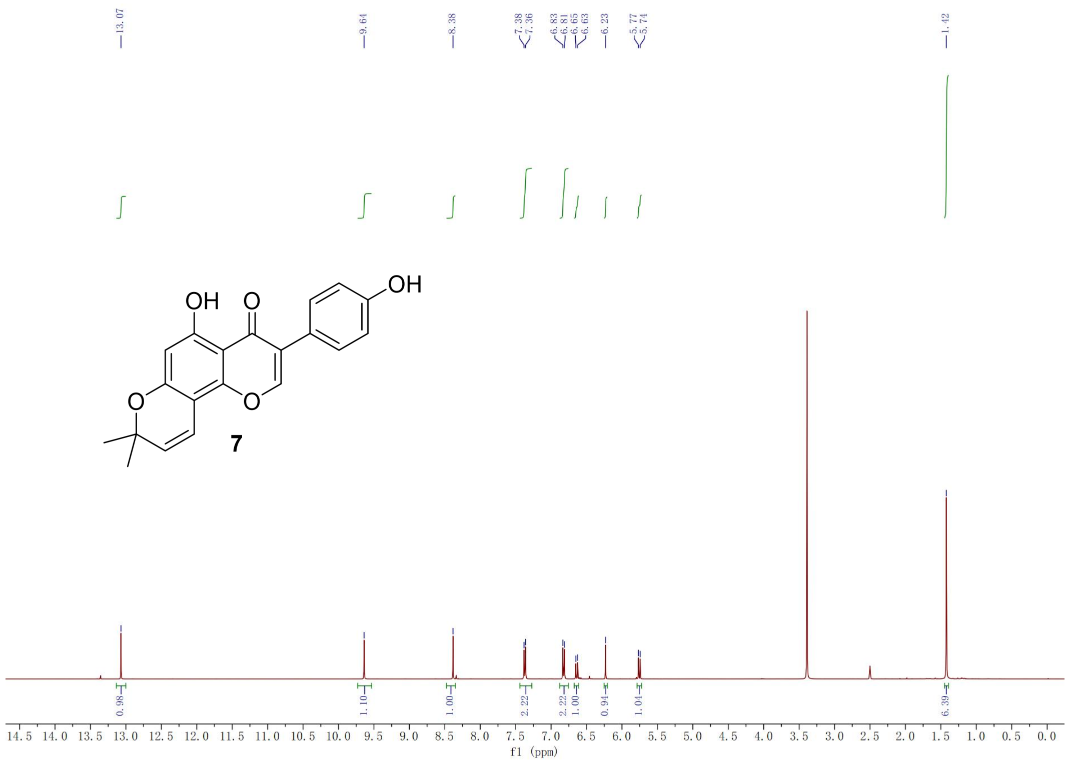

Figure S25 ^{1}H NMR (400 MHz, $CDCl_3$) of compound **7**.

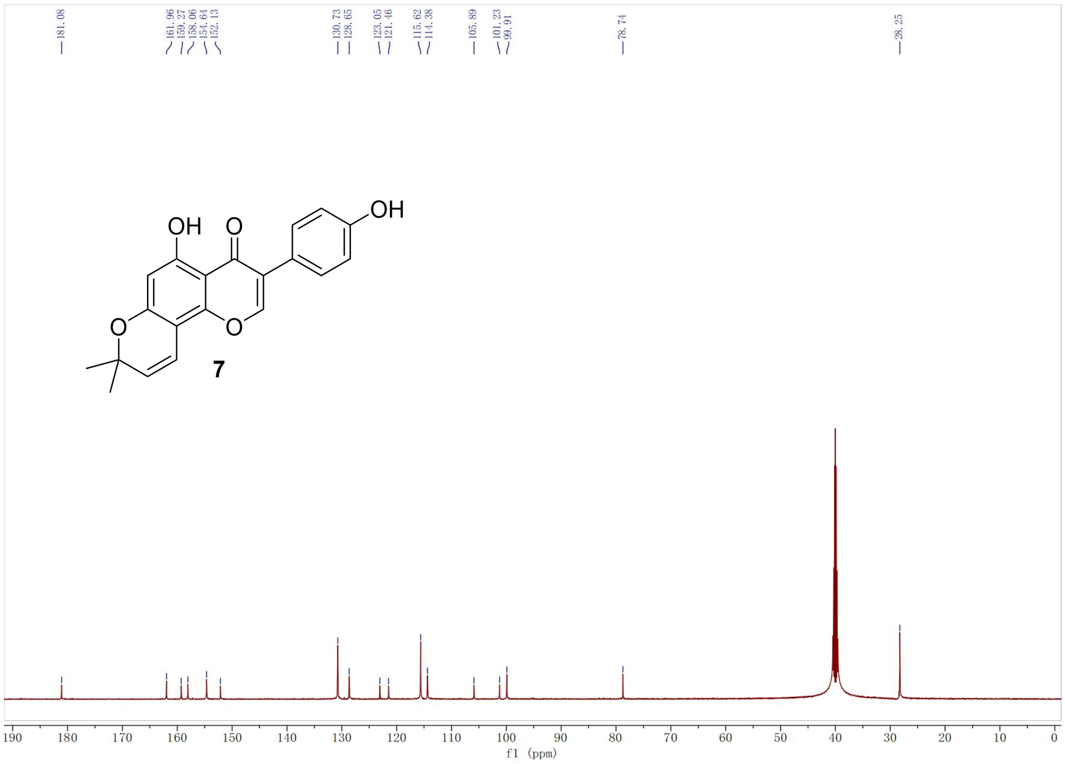

Figure S26 ^{13}C NMR (150 MHz, $CDCl_3$) of compound **7**.

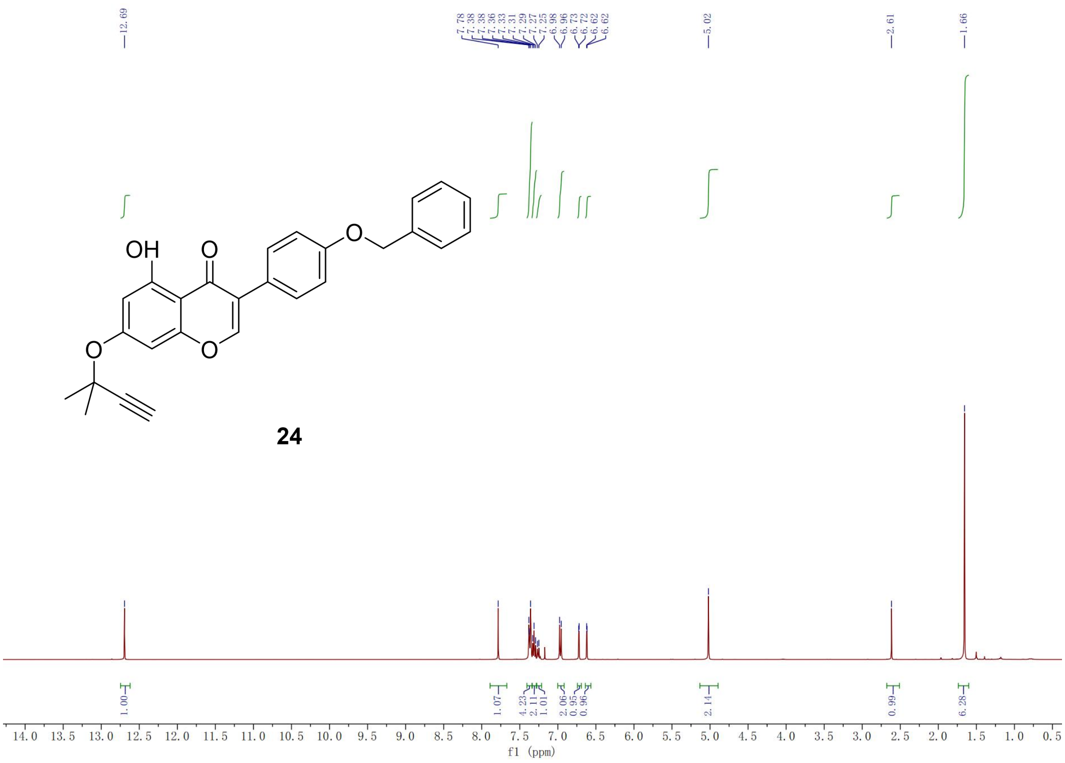

Figure S27 ^{1}H NMR (400 MHz, $CDCl_3$) of compound **24**.

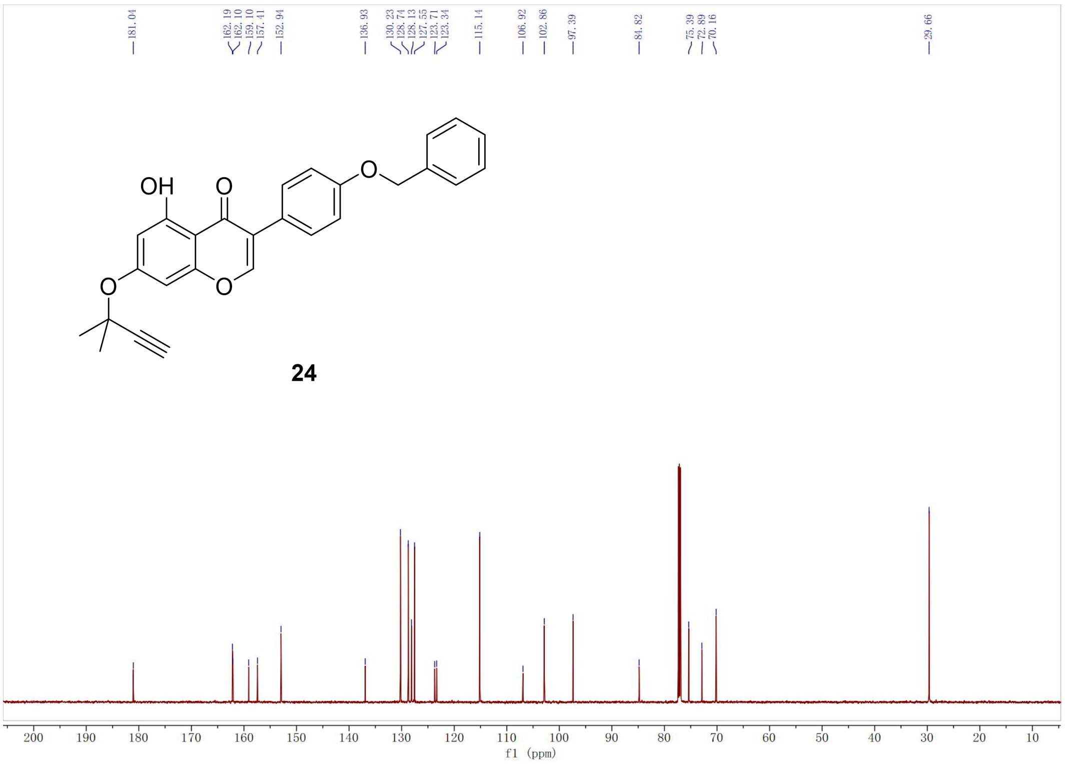

Figure S28 ^{13}C NMR(100 MHz, $CDCl_3$) of compound **24**.

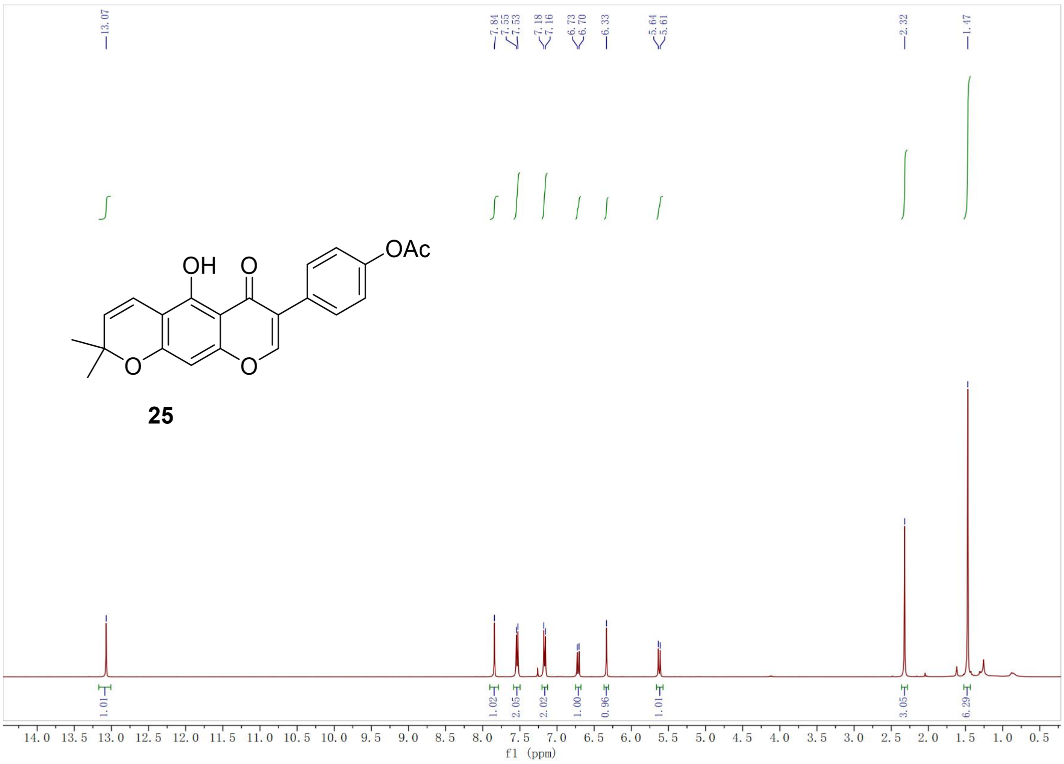

Figure S29 ^{1}H NMR (400 MHz, $CDCl_3$) of compound **25**.

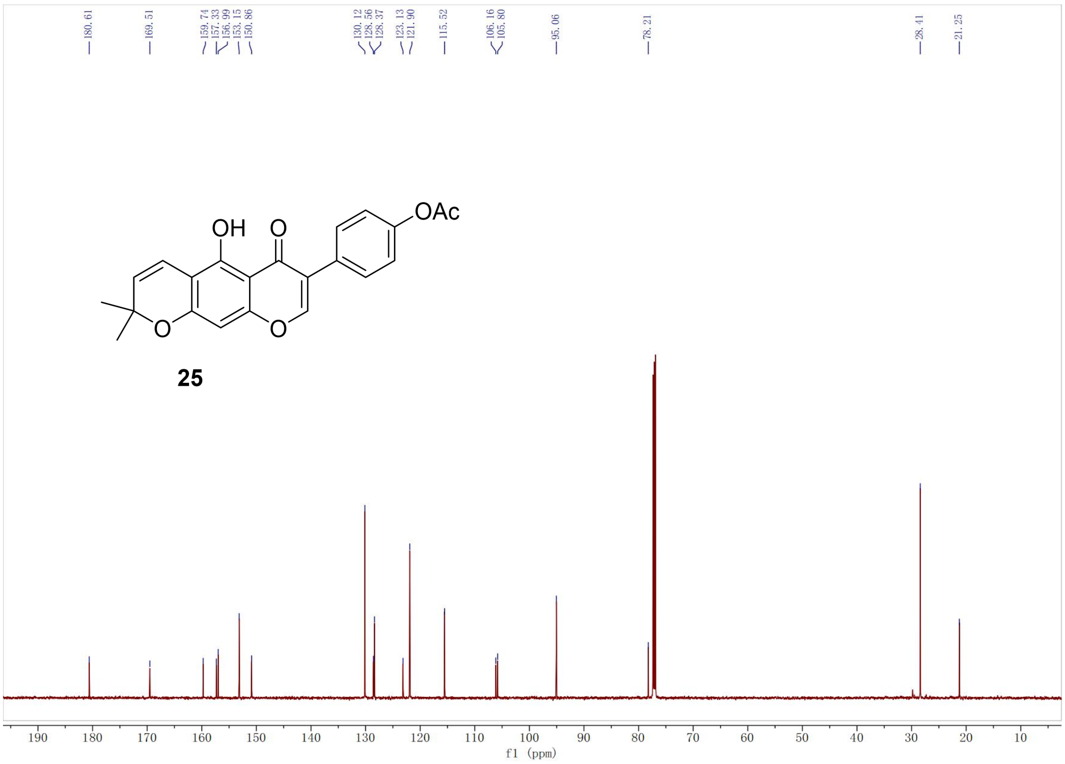

Figure S30 ^{13}C NMR (150 MHz, $CDCl_3$) of compound **25**.

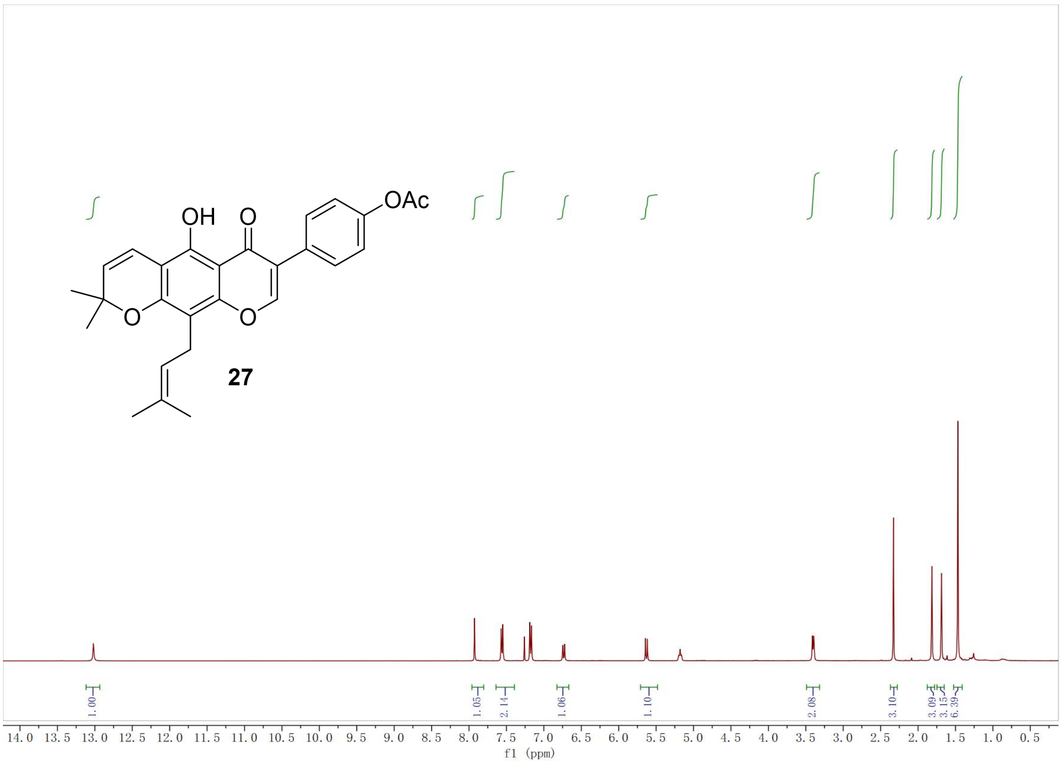

Figure S31 ^{1}H NMR (400 MHz, $CDCl_3$) of compound **27**.

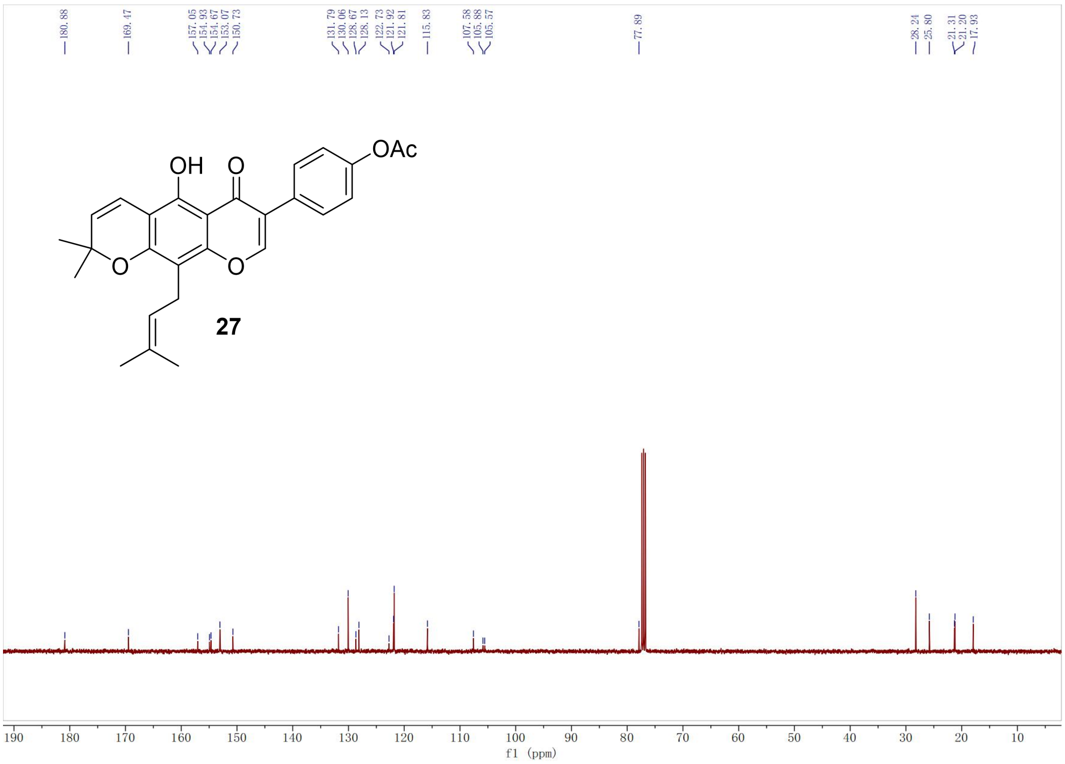

Figure S32 ^{13}C NMR (100 MHz, $CDCl_3$) of compound **27**.

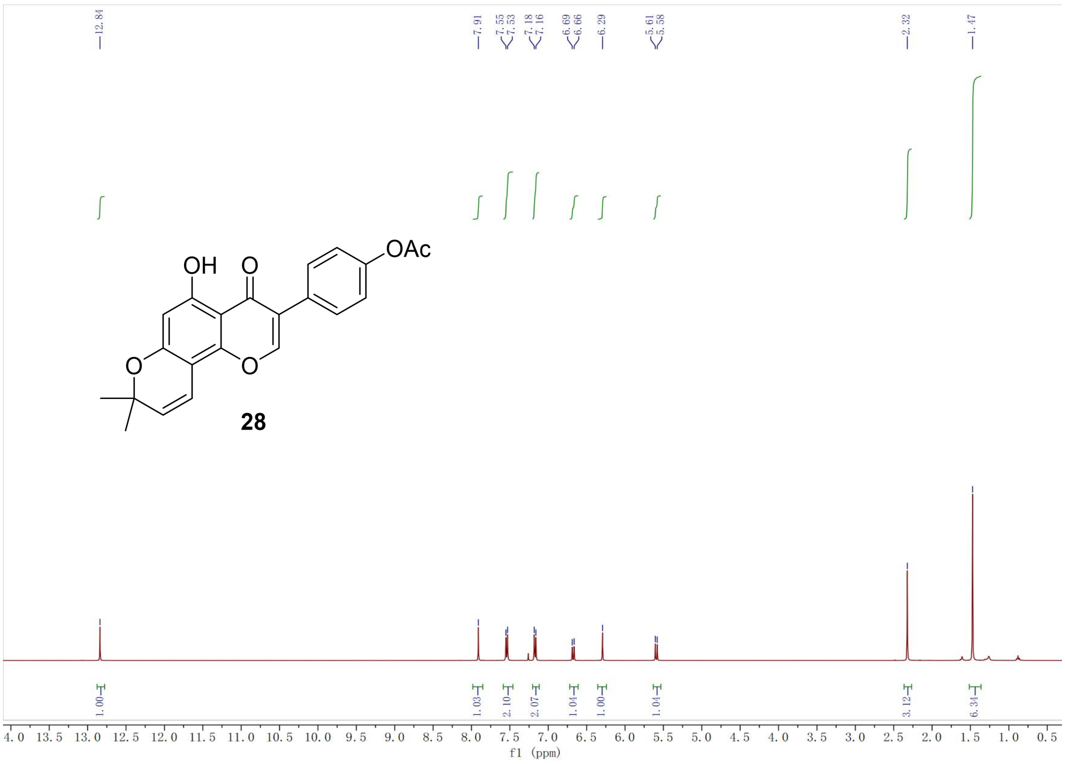

Figure S33 ¹H NMR (400 MHz, $CDCl_3$) of compound **28**.

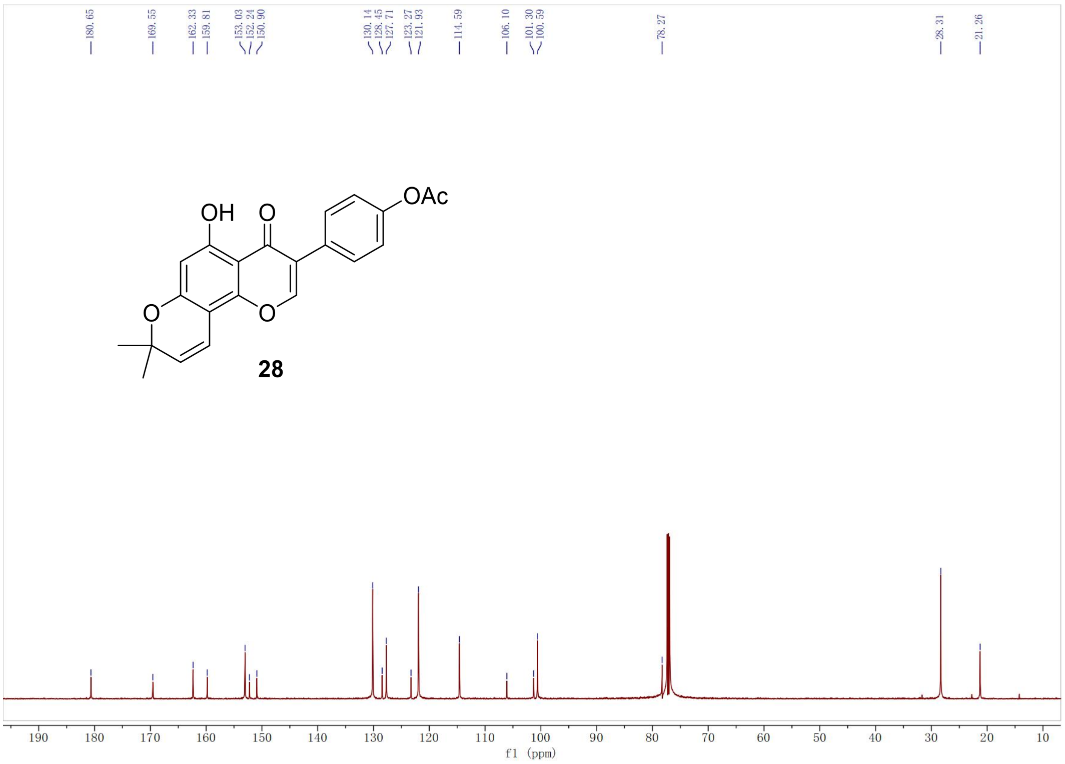

Figure S34 ¹³C NMR (150 MHz, $CDCl_3$) of compound **28**.

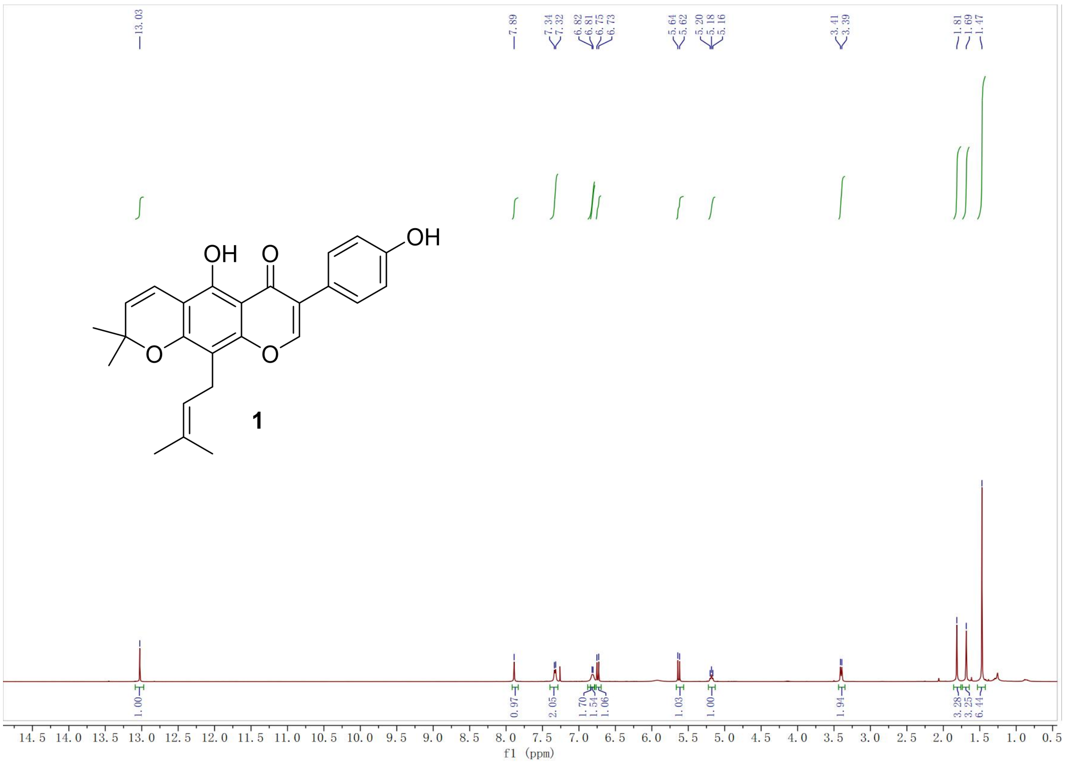

Figure S35 ^{1}H NMR (400 MHz, $CDCl_3$) of compound **1**.

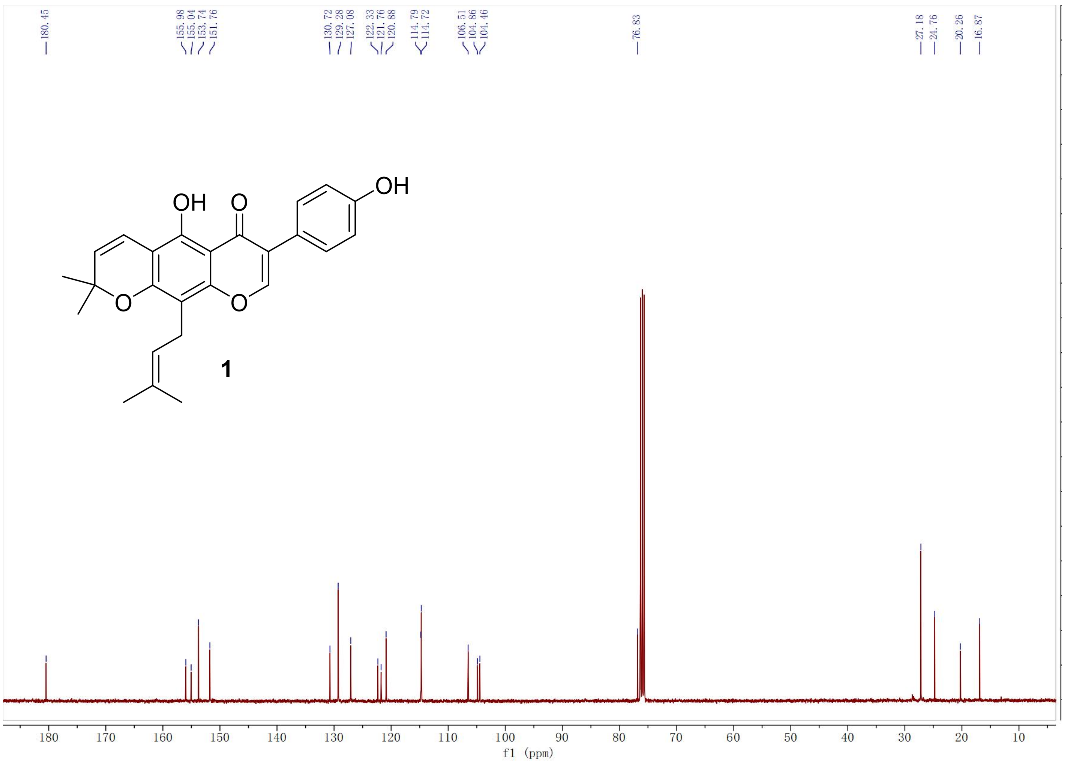

Figure S36 ^{13}C NMR (100 MHz, $CDCl_3$) of compound **1**.

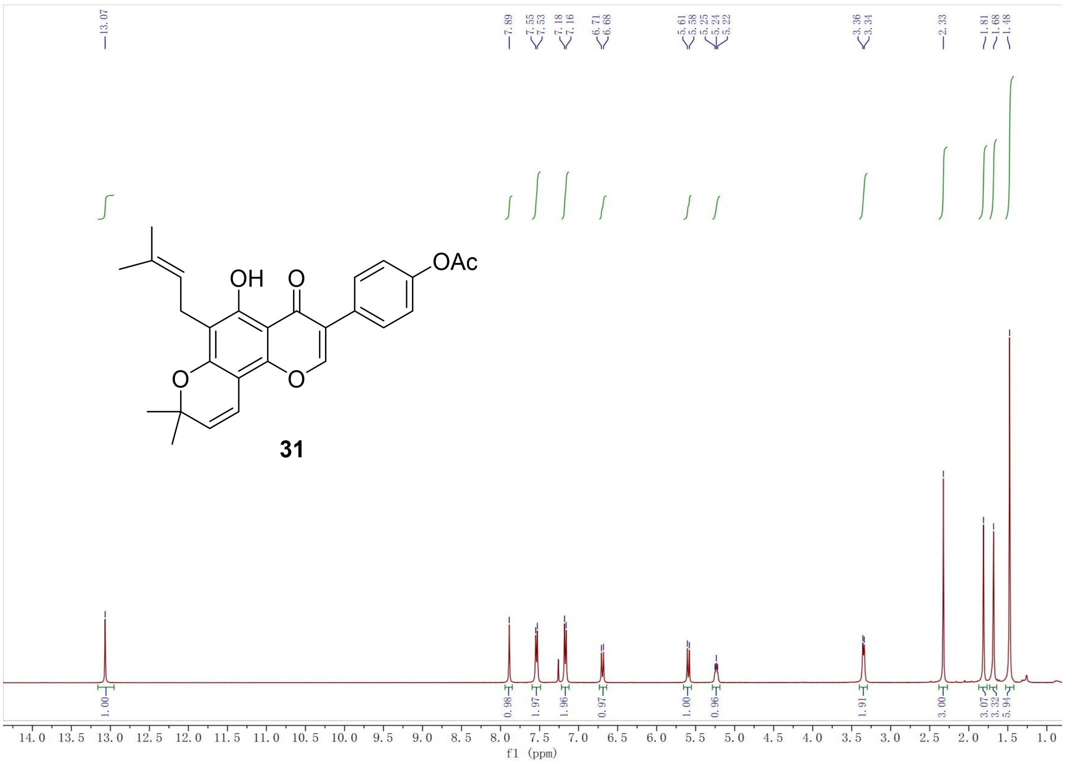

Figure S37 ^{1}H NMR (400 MHz, $CDCl_3$) of compound **31**.

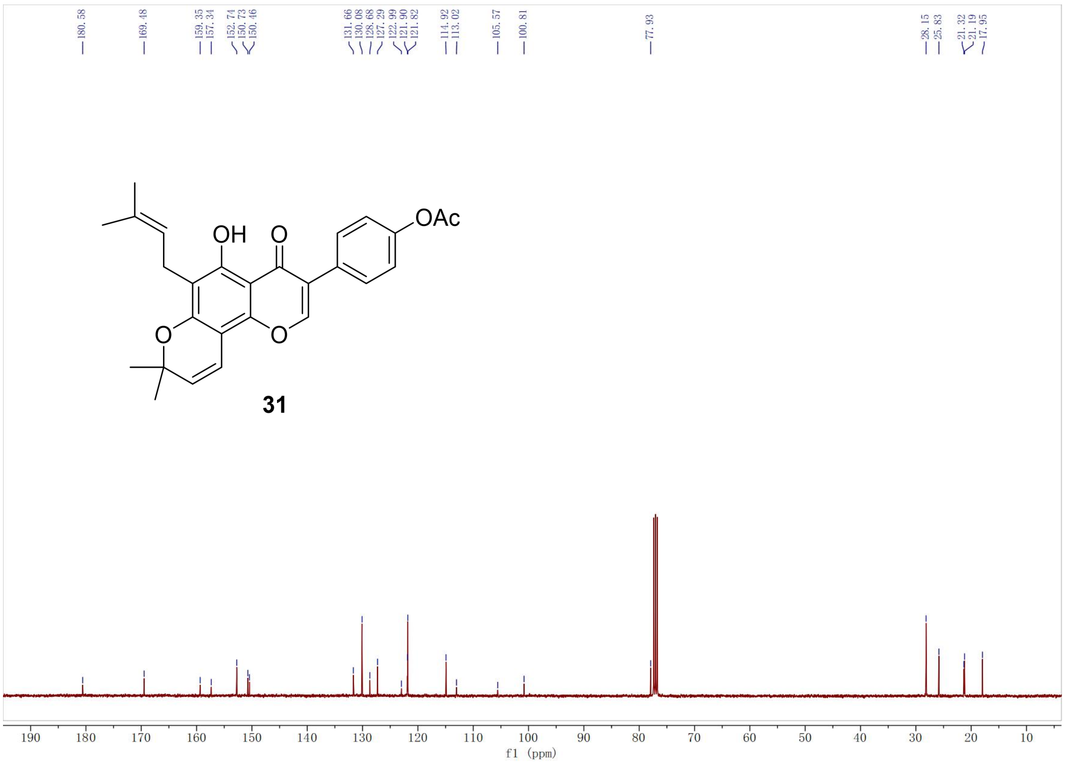

Figure S38 ^{13}C NMR (100 MHz, $CDCl_3$) of compound **31**.

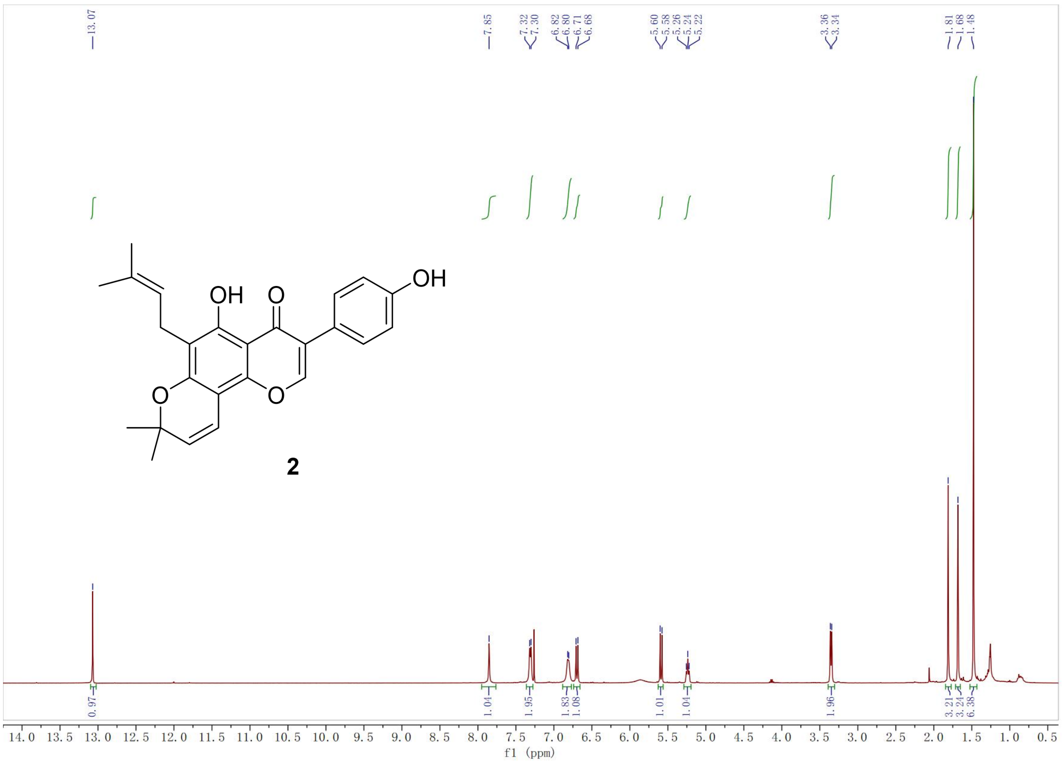

Figure S39 ^{1}H NMR (400 MHz, $CDCl_3$) of compound **2**.

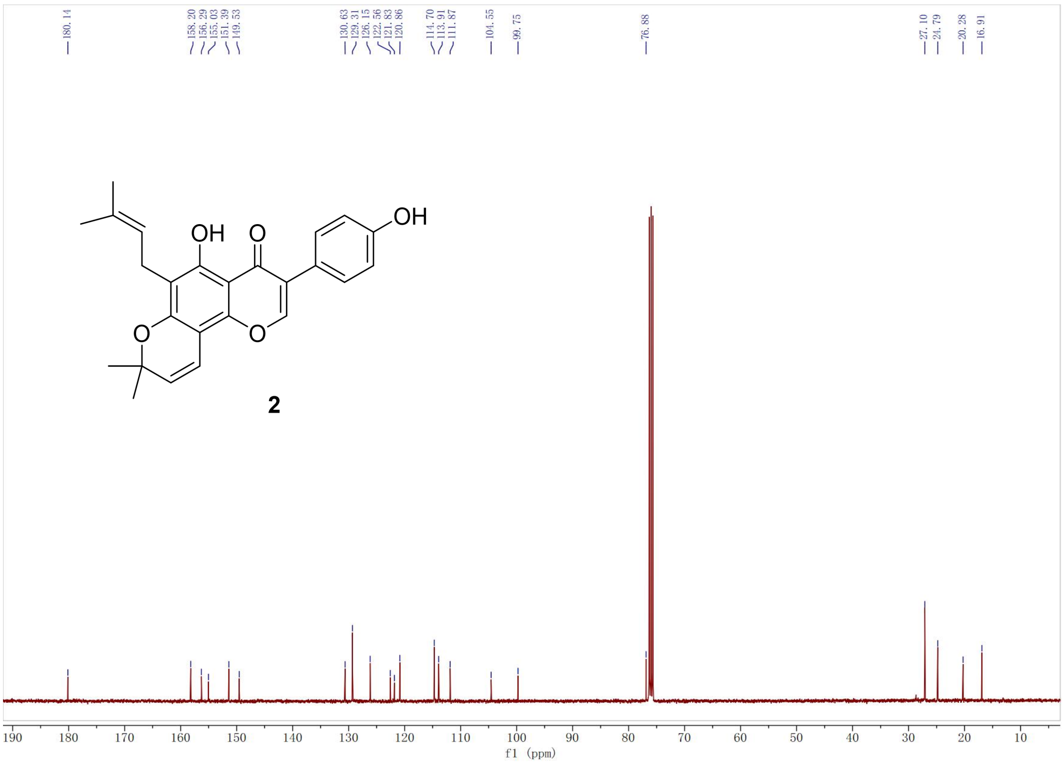

Figure S40 ^{13}C NMR (100 MHz, $CDCl_3$) of compound **2**.

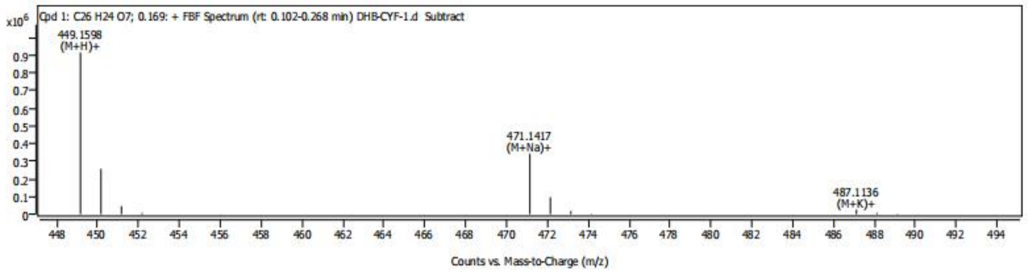

Spectrum Peaks

m/z	m/z (Calc)	Diff (ppm)	Abund	Height %	Height % (Calc)	Ion Species	Z
449.1598	449.1595	0.65	916881	100.00	100.00	(M+H)+	1
450.1633	450.1629	0.99	256754	28.00	28.68	(M+H)+	1
451.1658	451.1656	0.47	45460	4.96	5.40	(M+H)+	1
452.1700	452.1683	3.79	7707	0.84	0.76	(M+H)+	1
471.1417	471.1414	0.57	343422	100.00	100.00	(M+Na)+	1
472.1449	472.1448	0.08	96799	28.19	28.66	(M+Na)+	1
473.1475	473.1475	-0.07	17528	5.10	5.39	(M+Na)+	1
474.1507	474.1502	0.99	2718	0.79	0.76	(M+Na)+	1
487.1136	487.1154	-3.67	25391	100.00	100.00	(M+K)+	1
488.1163	488.1188	-4.98	7614	29.99	28.68	(M+K)+	1
489.1167	489.1169	-0.36	2945	11.60	12.61	(M+K)+	1
490.1201	490.1188	2.55	560	2.21	2.83	(M+K)+	1

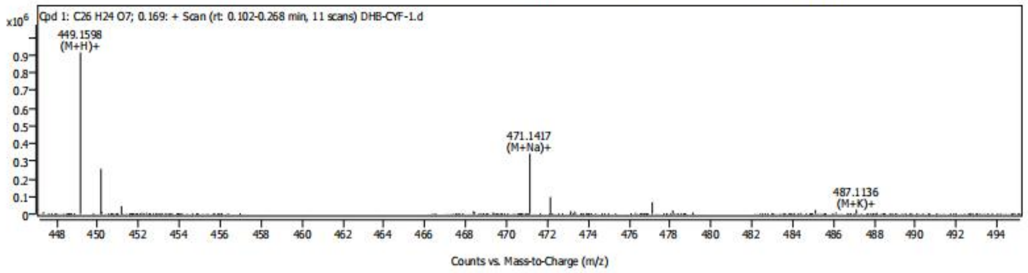

Spectrum Peaks

m/z	m/z (Calc)	Diff (ppm)	Abund	Height %	Height % (Calc)	Ion Species	Z
449.1598	449.1595	0.65	916881	100.00	100.00	(M+H)+	1
450.1633	450.1629	0.99	256754	28.00	28.68	(M+H)+	1
451.1658	451.1656	0.47	45460	4.96	5.40	(M+H)+	1
452.1700	452.1683	3.79	7707	0.84	0.76	(M+H)+	1
471.1417	471.1414	0.57	343422	100.00	100.00	(M+Na)+	1
472.1449	472.1448	0.08	96799	28.19	28.66	(M+Na)+	1
473.1475	473.1475	-0.07	17528	5.10	5.39	(M+Na)+	1
474.1507	474.1502	0.99	2718	0.79	0.76	(M+Na)+	1
487.1136	487.1154	-3.67	25391	100.00	100.00	(M+K)+	1
488.1163	488.1188	-4.98	7614	29.99	28.68	(M+K)+	1
489.1167	489.1169	-0.36	2945	11.60	12.61	(M+K)+	1
490.1201	490.1188	2.55	560	2.21	2.83	(M+K)+	1

Header
Data

Figure S41 HRMS (ESI) of compound **14**.

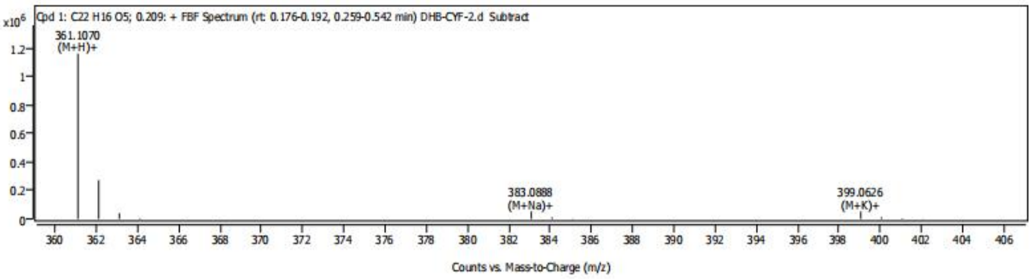

Spectrum Peaks

m/z	m/z (Calc)	Diff (ppm)	Abund	Height %	Height % (Calc)	Ion Species	Z
361.1070	361.1071	-0.11	1159666	100.00	100.00	(M+H)+	1
362.1104	362.1104	-0.23	272353	23.49	24.18	(M+H)+	1
363.1131	363.1131	-0.14	40971	3.53	3.82	(M+H)+	1
364.1159	364.1158	0.08	5266	0.45	0.45	(M+H)+	1
383.0888	383.0890	-0.56	53457	100.00	100.00	(M+Na)+	1
384.0921	384.0924	-0.85	11800	22.07	24.17	(M+Na)+	1
385.0957	385.0951	1.47	2275	4.26	3.82	(M+Na)+	1
399.0626	399.0629	-0.76	53137	100.00	100.00	(M+K)+	1
400.0661	400.0663	-0.65	12249	23.05	24.18	(M+K)+	1
401.0640	401.0638	0.58	4839	9.11	11.04	(M+K)+	1
402.0666	402.0659	1.60	1243	2.34	2.20	(M+K)+	1

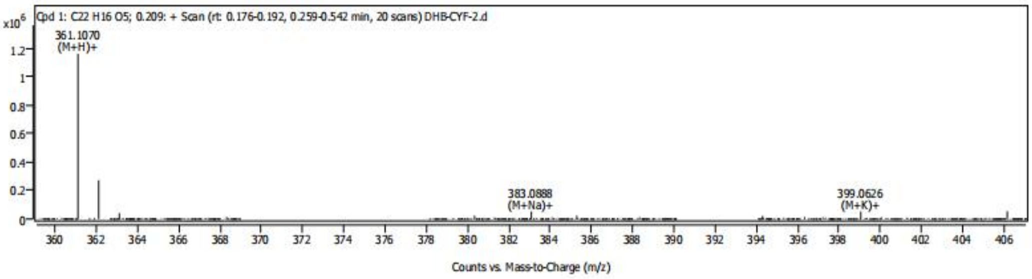

Spectrum Peaks

m/z	m/z (Calc)	Diff (ppm)	Abund	Height %	Height % (Calc)	Ion Species	Z
361.1070	361.1071	-0.11	1159666	100.00	100.00	(M+H)+	1
362.1104	362.1104	-0.23	272353	23.49	24.18	(M+H)+	1
363.1131	363.1131	-0.14	40971	3.53	3.82	(M+H)+	1
364.1159	364.1158	0.08	5266	0.45	0.45	(M+H)+	1
383.0888	383.0890	-0.56	53457	100.00	100.00	(M+Na)+	1
384.0921	384.0924	-0.85	11800	22.07	24.17	(M+Na)+	1
385.0957	385.0951	1.47	2275	4.26	3.82	(M+Na)+	1
399.0626	399.0629	-0.76	53137	100.00	100.00	(M+K)+	1
400.0661	400.0663	-0.65	12249	23.05	24.18	(M+K)+	1
401.0640	401.0638	0.58	4839	9.11	11.04	(M+K)+	1
402.0666	402.0659	1.60	1243	2.34	2.20	(M+K)+	1

Header
Data

Figure S42 HRMS (ESI) of compound **15**.

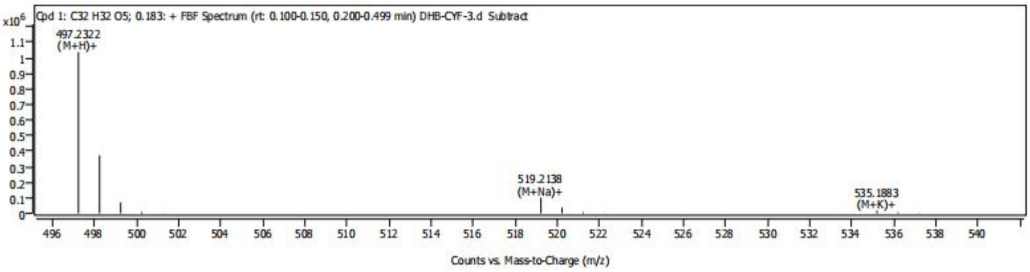

Spectrum Peaks

m/z	m/z (Calc)	Diff (ppm)	Abund	Height %	Height % (Calc)	Ion Species	Z
497.2322	497.2323	-0.02	1038439	100.00	100.00	(M+H)+	1
498.2357	498.2356	0.21	372964	35.92	35.18	(M+H)+	1
499.2384	499.2387	-0.59	70962	6.83	7.03	(M+H)+	1
500.2415	500.2415	-0.11	10055	0.97	1.02	(M+H)+	1
501.2446	501.2443	0.56	1315	0.13	0.12	(M+H)+	1
519.2138	519.2142	-0.75	101464	100.00	100.00	(M+Na)+	1
520.2172	520.2176	-0.78	37134	36.60	35.17	(M+Na)+	1
521.2206	521.2206	-0.06	7705	7.59	7.02	(M+Na)+	1
522.2244	522.2235	1.69	1328	1.31	1.02	(M+Na)+	1
535.1883	535.1881	0.28	17350	100.00	100.00	(M+K)+	1
536.1875	536.1915	-7.53	7062	40.70	35.18	(M+K)+	1
537.1852	537.1903	-9.62	2338	13.47	14.25	(M+K)+	1

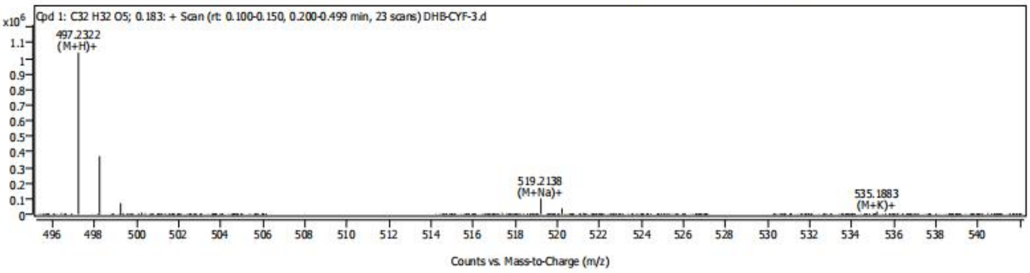

Spectrum Peaks

m/z	m/z (Calc)	Diff (ppm)	Abund	Height %	Height % (Calc)	Ion Species	Z
497.2322	497.2323	-0.02	1038439	100.00	100.00	(M+H)+	1
498.2357	498.2356	0.21	372964	35.92	35.18	(M+H)+	1
499.2384	499.2387	-0.59	70962	6.83	7.03	(M+H)+	1
500.2415	500.2415	-0.11	10055	0.97	1.02	(M+H)+	1
501.2446	501.2443	0.56	1315	0.13	0.12	(M+H)+	1
519.2138	519.2142	-0.75	101464	100.00	100.00	(M+Na)+	1
520.2172	520.2176	-0.78	37134	36.60	35.17	(M+Na)+	1
521.2206	521.2206	-0.06	7705	7.59	7.02	(M+Na)+	1
522.2244	522.2235	1.69	1328	1.31	1.02	(M+Na)+	1
535.1883	535.1881	0.28	17350	100.00	100.00	(M+K)+	1
536.1875	536.1915	-7.53	7062	40.70	35.18	(M+K)+	1
537.1852	537.1903	-9.62	2338	13.47	14.25	(M+K)+	1

Header
Data

Figure S43 HRMS (ESI) of compound **16**.

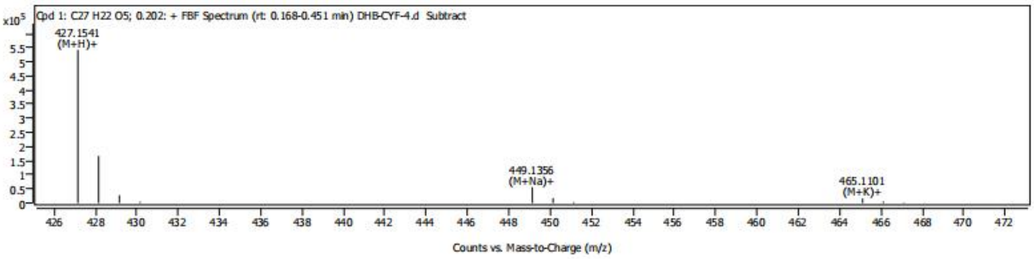

Spectrum Peaks

m/z	m/z (Calc)	Diff (ppm)	Abund	Height %	Height % (Calc)	Ion Species	Z
427.1541	427.1540	0.15	542570	100.00	100.00	(M+H)+	1
428.1572	428.1574	-0.49	166793	30.74	29.66	(M+H)+	1
429.1602	429.1603	-0.08	27954	5.15	5.27	(M+H)+	1
430.1649	430.1631	4.23	4572	0.84	0.69	(M+H)+	1
449.1356	449.1359	-0.71	56160	100.00	100.00	(M+Na)+	1
450.1391	450.1393	-0.60	17602	31.34	29.65	(M+Na)+	1
451.1425	451.1422	0.56	3283	5.85	5.26	(M+Na)+	1
465.1101	465.1099	0.44	16270	100.00	100.00	(M+K)+	1
466.1140	466.1133	1.63	5111	31.41	29.66	(M+K)+	1
467.1111	467.1114	-0.77	2197	13.51	12.48	(M+K)+	1
468.1179	468.1132	9.99	660	4.05	2.83	(M+K)+	1

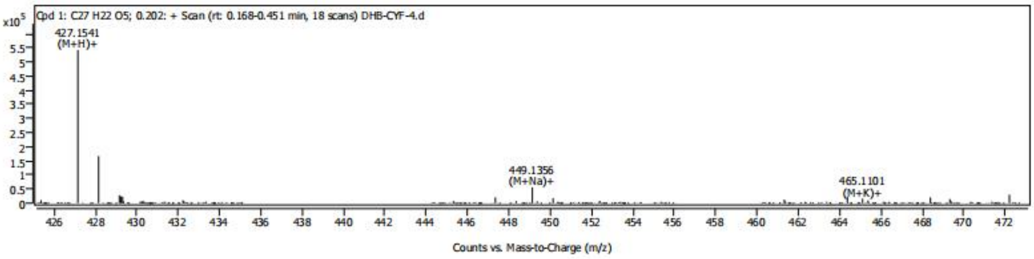

Spectrum Peaks

m/z	m/z (Calc)	Diff (ppm)	Abund	Height %	Height % (Calc)	Ion Species	Z
427.1541	427.1540	0.15	542570	100.00	100.00	(M+H)+	1
428.1572	428.1574	-0.49	166793	30.74	29.66	(M+H)+	1
429.1602	429.1603	-0.08	27954	5.15	5.27	(M+H)+	1
430.1649	430.1631	4.23	4572	0.84	0.69	(M+H)+	1
449.1356	449.1359	-0.71	56160	100.00	100.00	(M+Na)+	1
450.1391	450.1393	-0.60	17602	31.34	29.65	(M+Na)+	1
451.1425	451.1422	0.56	3283	5.85	5.26	(M+Na)+	1
465.1101	465.1099	0.44	16270	100.00	100.00	(M+K)+	1
466.1140	466.1133	1.63	5111	31.41	29.66	(M+K)+	1
467.1111	467.1114	-0.77	2197	13.51	12.48	(M+K)+	1
468.1179	468.1132	9.99	660	4.05	2.83	(M+K)+	1

Header
Data

Figure S44 HRMS (ESI) of compound **19**.

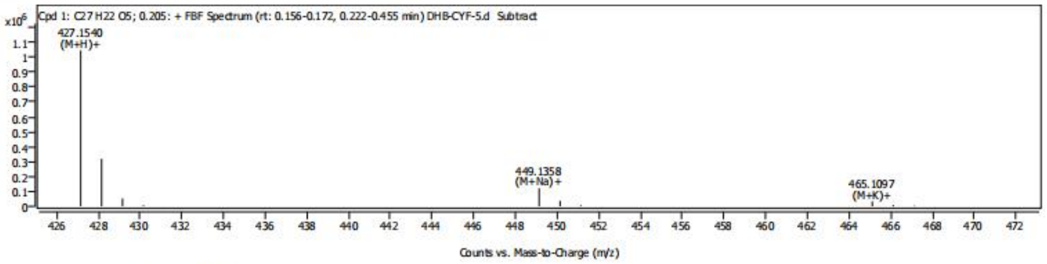

Spectrum Peaks

m/z	m/z (Calc)	Diff (ppm)	Abund	Height %	Height % (Calc)	Ion Species	Z
427.1540	427.1540	0.05	1045528	100.00	100.00	(M+H)+	1
428.1574	428.1574	0.00	318529	30.47	29.66	(M+H)+	1
429.1601	429.1603	-0.31	53216	5.09	5.27	(M+H)+	1
430.1636	430.1631	1.34	7420	0.71	0.69	(M+H)+	1
431.1670	431.1658	2.76	1036	0.10	0.07	(M+H)+	1
449.1358	449.1359	-0.42	121372	100.00	100.00	(M+Na)+	1
450.1392	450.1393	-0.30	36903	30.41	29.65	(M+Na)+	1
451.1424	451.1422	0.36	6758	5.57	5.26	(M+Na)+	1
452.1491	452.1450	9.11	1092	0.90	0.69	(M+Na)+	1
465.1097	465.1099	-0.33	33638	100.00	100.00	(M+K)+	1
466.1133	466.1133	0.10	10076	29.96	29.66	(M+K)+	1
467.1121	467.1114	1.40	3807	11.32	12.48	(M+K)+	1
468.1136	468.1132	0.69	1134	3.37	2.83	(M+K)+	1

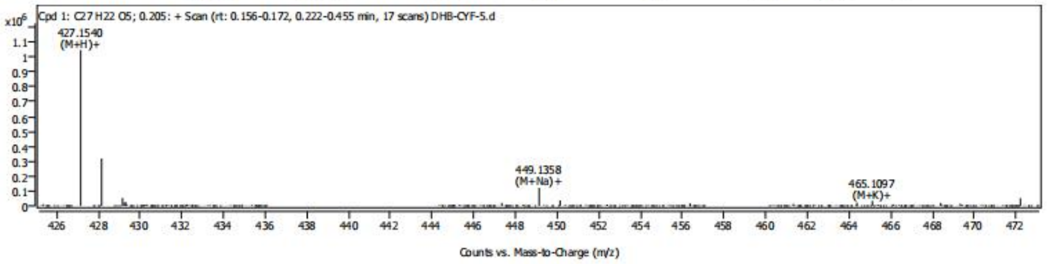

Spectrum Peaks

m/z	m/z (Calc)	Diff (ppm)	Abund	Height %	Height % (Calc)	Ion Species	Z
427.1540	427.1540	0.05	1045528	100.00	100.00	(M+H)+	1
428.1574	428.1574	0.00	318529	30.47	29.66	(M+H)+	1
429.1601	429.1603	-0.31	53216	5.09	5.27	(M+H)+	1
430.1636	430.1631	1.34	7420	0.71	0.69	(M+H)+	1
431.1670	431.1658	2.76	1036	0.10	0.07	(M+H)+	1
449.1358	449.1359	-0.42	121372	100.00	100.00	(M+Na)+	1
450.1392	450.1393	-0.30	36903	30.41	29.65	(M+Na)+	1
451.1424	451.1422	0.36	6758	5.57	5.26	(M+Na)+	1
452.1491	452.1450	9.11	1092	0.90	0.69	(M+Na)+	1
465.1097	465.1099	-0.33	33638	100.00	100.00	(M+K)+	1
466.1133	466.1133	0.10	10076	29.96	29.66	(M+K)+	1
467.1121	467.1114	1.40	3807	11.32	12.48	(M+K)+	1
468.1136	468.1132	0.69	1134	3.37	2.83	(M+K)+	1

Header
Data

Figure S45 HRMS (ESI) of compound **24**.

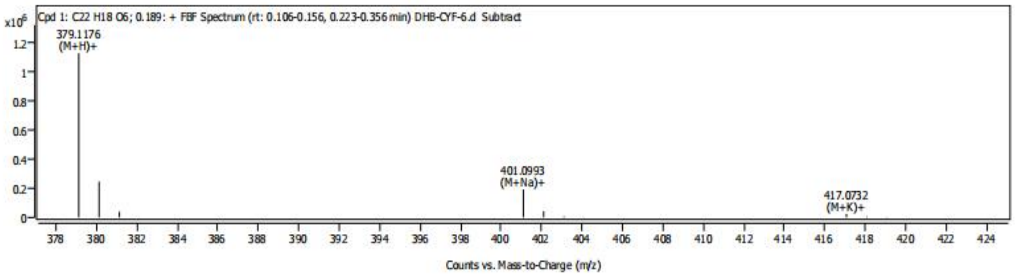

Spectrum Peaks

m/z	m/z (Calc)	Diff (ppm)	Abund	Height %	Height % (Calc)	Ion Species	Z
379.1176	379.1176	0.05	1129342	100.00	100.00	(M+H)+	1
380.1209	380.1210	-0.33	246657	21.84	24.24	(M+H)+	1
381.1234	381.1236	-0.64	39760	3.52	4.04	(M+H)+	1
401.0993	401.0996	-0.60	192528	100.00	100.00	(M+Na)+	1
402.1026	402.1029	-0.76	42935	22.30	24.23	(M+Na)+	1
403.1056	403.1056	0.07	7246	3.76	4.04	(M+Na)+	1
404.1105	404.1082	5.71	870	0.45	0.51	(M+Na)+	1
417.0732	417.0735	-0.66	24236	100.00	100.00	(M+K)+	1
418.0766	418.0769	-0.64	6514	26.88	24.24	(M+K)+	1
419.0756	419.0744	2.68	2579	10.64	11.26	(M+K)+	1
420.0780	420.0766	3.31	604	2.49	2.25	(M+K)+	1

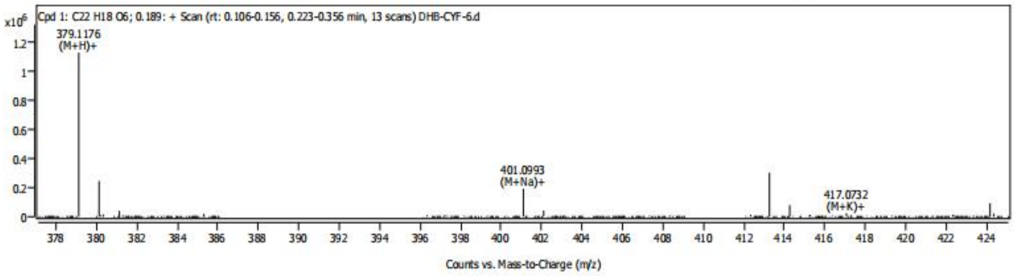

Spectrum Peaks

m/z	m/z (Calc)	Diff (ppm)	Abund	Height %	Height % (Calc)	Ion Species	Z
379.1176	379.1176	0.05	1129342	100.00	100.00	(M+H)+	1
380.1209	380.1210	-0.33	246657	21.84	24.24	(M+H)+	1
381.1234	381.1236	-0.64	39760	3.52	4.04	(M+H)+	1
401.0993	401.0996	-0.60	192528	100.00	100.00	(M+Na)+	1
402.1026	402.1029	-0.76	42935	22.30	24.23	(M+Na)+	1
403.1056	403.1056	0.07	7246	3.76	4.04	(M+Na)+	1
404.1105	404.1082	5.71	870	0.45	0.51	(M+Na)+	1
417.0732	417.0735	-0.66	24236	100.00	100.00	(M+K)+	1
418.0766	418.0769	-0.64	6514	26.88	24.24	(M+K)+	1
419.0756	419.0744	2.68	2579	10.64	11.26	(M+K)+	1
420.0780	420.0766	3.31	604	2.49	2.25	(M+K)+	1

Header
Data

Figure S46 HRMS (ESI) of compound **28**.

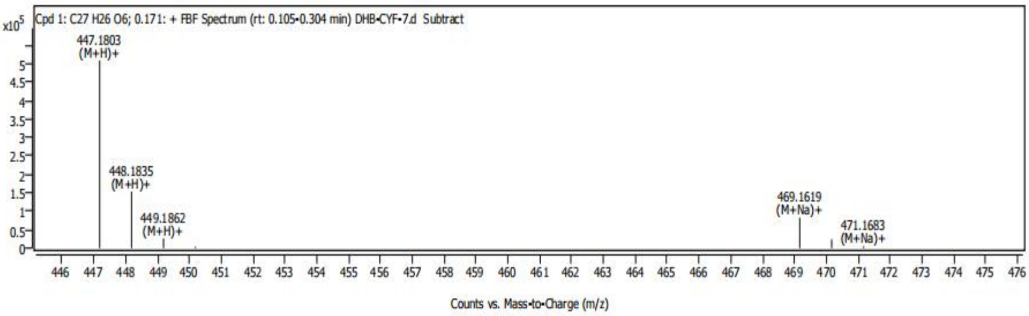

Spectrum Peaks

m/z	m/z (Calc)	Diff (ppm)	Abund	Height %	Height % (Calc)	Ion Species	Z
447.1803	447.1802	0.25	508650	100.00	100.00	(M+H)+	1
448.1835	448.1836	-0.31	153533	30.18	29.74	(M+H)+	1
449.1862	449.1864	-0.60	26464	5.20	5.50	(M+H)+	1
450.1892	450.1892	0.06	4040	0.79	0.76	(M+H)+	1
469.1619	469.1622	-0.51	82536	100.00	100.00	(M+Na)+	1
470.1652	470.1656	-0.70	23832	28.87	29.73	(M+Na)+	1
471.1683	471.1684	-0.07	5326	6.45	5.49	(M+Na)+	1

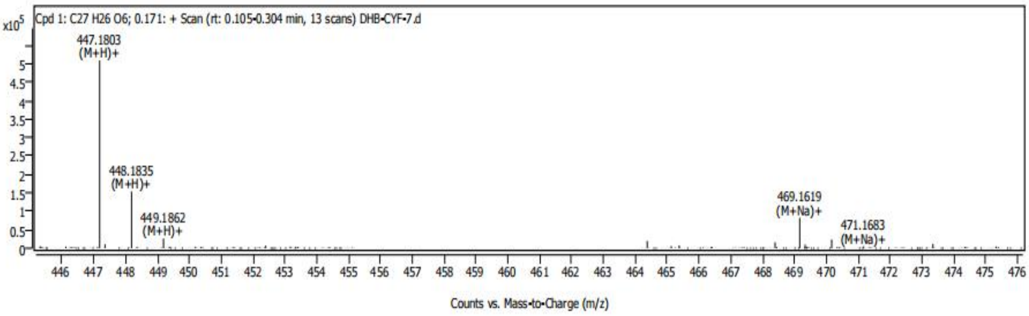

Spectrum Peaks

m/z	m/z (Calc)	Diff (ppm)	Abund	Height %	Height % (Calc)	Ion Species	Z
447.1803	447.1802	0.25	508650	100.00	100.00	(M+H)+	1
448.1835	448.1836	-0.31	153533	30.18	29.74	(M+H)+	1
449.1862	449.1864	-0.60	26464	5.20	5.50	(M+H)+	1
450.1892	450.1892	0.06	4040	0.79	0.76	(M+H)+	1
469.1619	469.1622	-0.51	82536	100.00	100.00	(M+Na)+	1
470.1652	470.1656	-0.70	23832	28.87	29.73	(M+Na)+	1
471.1683	471.1684	-0.07	5326	6.45	5.49	(M+Na)+	1

Header
Data

Figure S47 HRMS (ESI) of compound **31**.

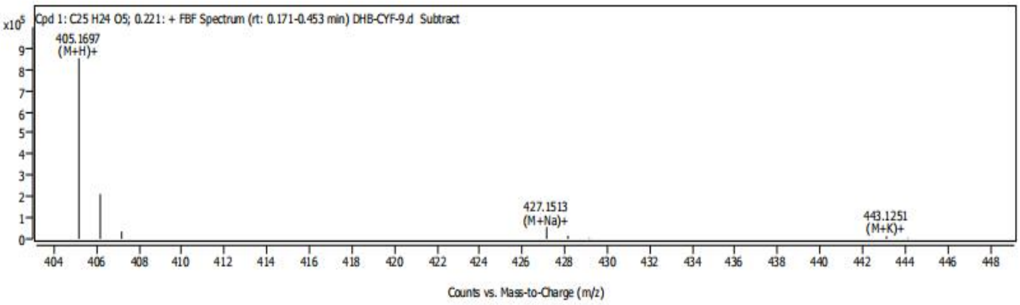

Spectrum Peaks

m/z	m/z (Calc)	Diff (ppm)	Abund	Height %	Height % (Calc)	Ion Species	Z
405.1697	405.1697	0.21	853070	100.00	100.00	(M+H)+	1
406.1728	406.1730	-0.51	211971	24.85	27.52	(M+H)+	1
407.1758	407.1759	-0.29	35385	4.15	4.67	(M+H)+	1
427.1513	427.1516	-0.58	56095	100.00	100.00	(M+Na)+	1
428.1549	428.1550	-0.25	15158	27.02	27.51	(M+Na)+	1
429.1579	429.1578	0.24	3075	5.48	4.66	(M+Na)+	1
443.1251	443.1255	-1.03	12945	100.00	100.00	(M+K)+	1
444.1289	444.1289	-0.04	3331	25.73	27.52	(M+K)+	1

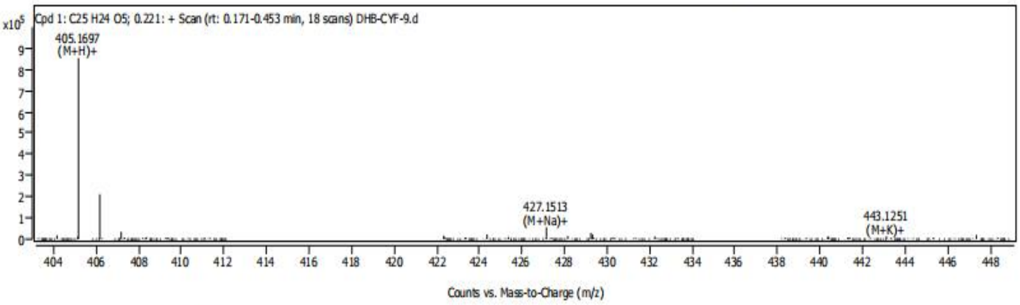

Spectrum Peaks

m/z	m/z (Calc)	Diff (ppm)	Abund	Height %	Height % (Calc)	Ion Species	Z
405.1697	405.1697	0.21	853070	100.00	100.00	(M+H)+	1
406.1728	406.1730	-0.51	211971	24.85	27.52	(M+H)+	1
407.1758	407.1759	-0.29	35385	4.15	4.67	(M+H)+	1
427.1513	427.1516	-0.58	56095	100.00	100.00	(M+Na)+	1
428.1549	428.1550	-0.25	15158	27.02	27.51	(M+Na)+	1
429.1579	429.1578	0.24	3075	5.48	4.66	(M+Na)+	1
443.1251	443.1255	-1.03	12945	100.00	100.00	(M+K)+	1
444.1289	444.1289	-0.04	3331	25.73	27.52	(M+K)+	1

Header
Data

Figure S48 HRMS (ESI) of compound **1**.

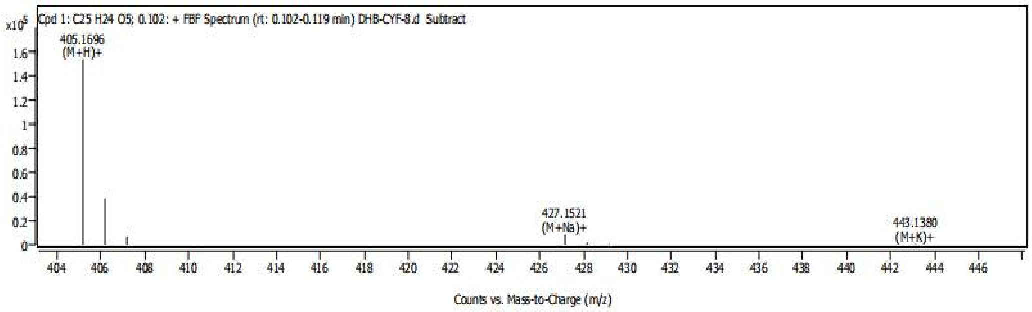

Spectrum Peaks

m/z	m/z (Calc)	Diff (ppm)	Abund	Height %	Height % (Calc)	Ion Species	Z
405.1696	405.1697	-0.02	153539	100.00	100.00	(M+H)+	1
406.1728	406.1730	-0.55	38249	24.91	27.52	(M+H)+	1
407.1765	407.1759	1.56	6741	4.39	4.67	(M+H)+	1
427.1521	427.1516	1.20	8354	100.00	100.00	(M+Na)+	1
428.1568	428.1550	4.20	2217	26.54	27.51	(M+Na)+	1
429.1611	429.1578	7.74	664	7.95	4.66	(M+Na)+	1
443.1380	443.1255	28.12	656	100.00	100.00	(M+K)+	1

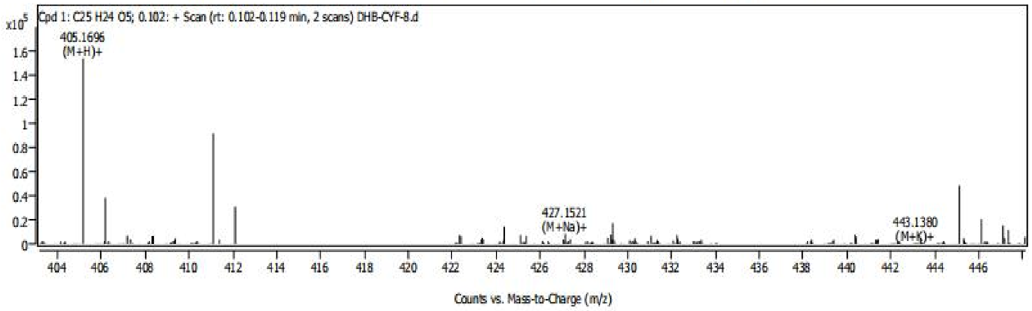

Spectrum Peaks

m/z	m/z (Calc)	Diff (ppm)	Abund	Height %	Height % (Calc)	Ion Species	Z
405.1696	405.1697	-0.02	153539	100.00	100.00	(M+H)+	1
406.1728	406.1730	-0.55	38249	24.91	27.52	(M+H)+	1
407.1765	407.1759	1.56	6741	4.39	4.67	(M+H)+	1
427.1521	427.1516	1.20	8354	100.00	100.00	(M+Na)+	1
428.1568	428.1550	4.20	2217	26.54	27.51	(M+Na)+	1
429.1611	429.1578	7.74	664	7.95	4.66	(M+Na)+	1
443.1380	443.1255	28.12	656	100.00	100.00	(M+K)+	1

Header
Data

Figure S49 HRMS (ESI) of compound **2**.

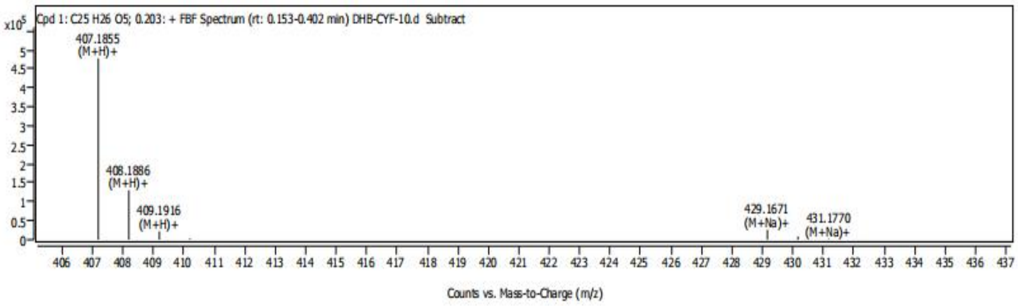

Spectrum Peaks

m/z	m/z (Calc)	Diff (ppm)	Abund	Height %	Height % (Calc)	Ion Species	Z
407.1855	407.1853	0.49	478163	100.00	100.00	(M+H)+	1
408.1886	408.1887	-0.29	130402	27.27	27.54	(M+H)+	1
409.1916	409.1915	0.10	21954	4.59	4.67	(M+H)+	1
410.1951	410.1943	1.90	2817	0.59	0.59	(M+H)+	1
429.1671	429.1672	-0.36	26146	100.00	100.00	(M+Na)+	1
430.1709	430.1706	0.72	8634	33.02	27.53	(M+Na)+	1
431.1770	431.1735	8.17	1970	7.53	4.67	(M+Na)+	1
432.1736	432.1762	-6.04	115	0.44	0.59	(M+Na)+	1

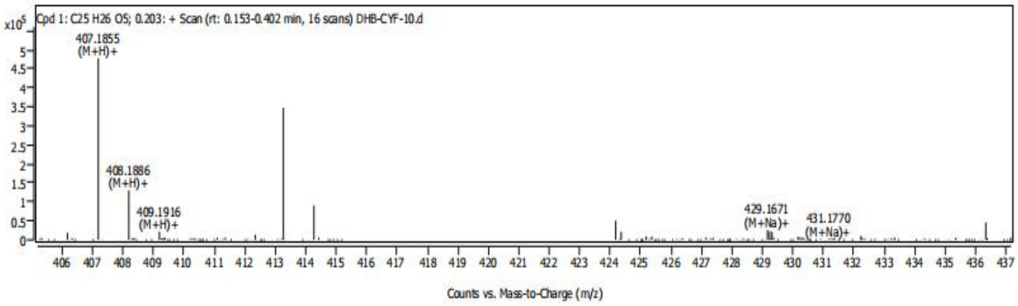

Spectrum Peaks

m/z	m/z (Calc)	Diff (ppm)	Abund	Height %	Height % (Calc)	Ion Species	Z
407.1855	407.1853	0.49	478163	100.00	100.00	(M+H)+	1
408.1886	408.1887	-0.29	130402	27.27	27.54	(M+H)+	1
409.1916	409.1915	0.10	21954	4.59	4.67	(M+H)+	1
410.1951	410.1943	1.90	2817	0.59	0.59	(M+H)+	1
429.1671	429.1672	-0.36	26146	100.00	100.00	(M+Na)+	1
430.1709	430.1706	0.72	8634	33.02	27.53	(M+Na)+	1
431.1770	431.1735	8.17	1970	7.53	4.67	(M+Na)+	1
432.1736	432.1762	-6.04	115	0.44	0.59	(M+Na)+	1

Header
Data

Figure S50 HRMS (ESI) of compound **3**.

Table S1. Crystal data and structure refinement for 1

Empirical formula	$C_{25}H_{24}O_5$
Formula weight	404.44
Temperature/K	210.0
Crystal system	triclinic
Space group (number)	$P\bar{1}$ (2)
a/Å	6.1838(5)
b/Å	8.1570(6)
c/Å	21.1051(18)
α/°	97.443(3)
β/°	96.496(3)
γ/°	101.646(3)
Volume/Å^3	1023.18(14)
Z	2
ρ_{calc} g/cm^{-3}	1.313
μ/mm^{-1}	0.091
F(000)	428.0
Crystal size/mm^3	0.47×0.2×0.07
Radiation	MoK_α (λ=0.71073)
2θ range for data collection/°	5.162 to 55.032
Index ranges	$-8 \le h \le 8, -10 \le k \le 10, -27 \le l \le 27$

Reflections collected	17159
Independent reflections	4651 [R_{int} = 0.1146, R_{sigma} = 0.0987]
Data/Restraints/Parameters	4651/0/277
Goodness-of-fit on F^2	1.030
Final R indexes [$I \geq 2\sigma(I)$]	R_1 = 0.0573, wR_2 = 0.1310
Final R indexes [all data]	R_1 = 0.1228, wR_2 = 0.1587
Largest peak/hole / e Å^{-3}	0.22/−0.20

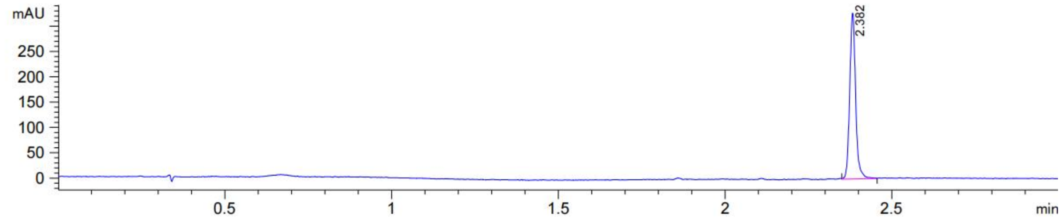

RetTim	Width	Area	Height	Area%
2.38	0.02	522.00	426.79	100.00

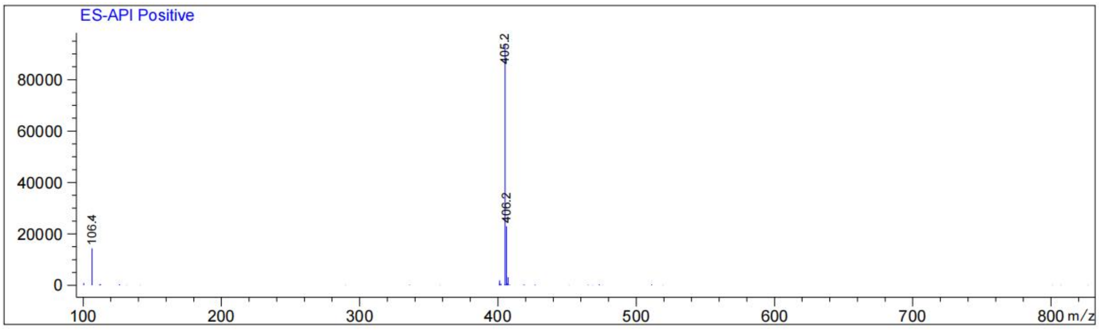

Figure S51 LCMS report of compound **1**

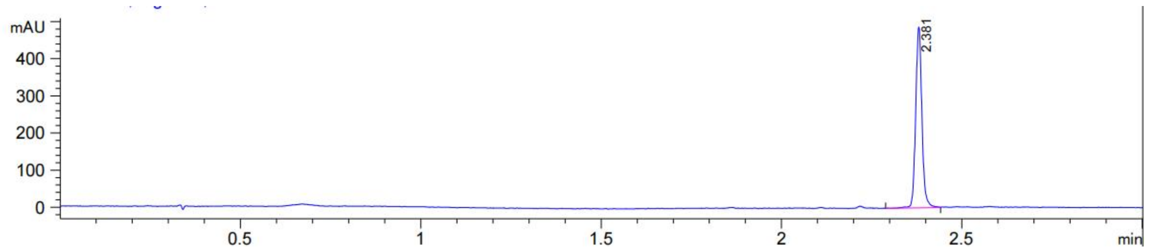

RetTim	Width	Area	Height	Area%
2.38	0.02	587.18	486.97	100.00

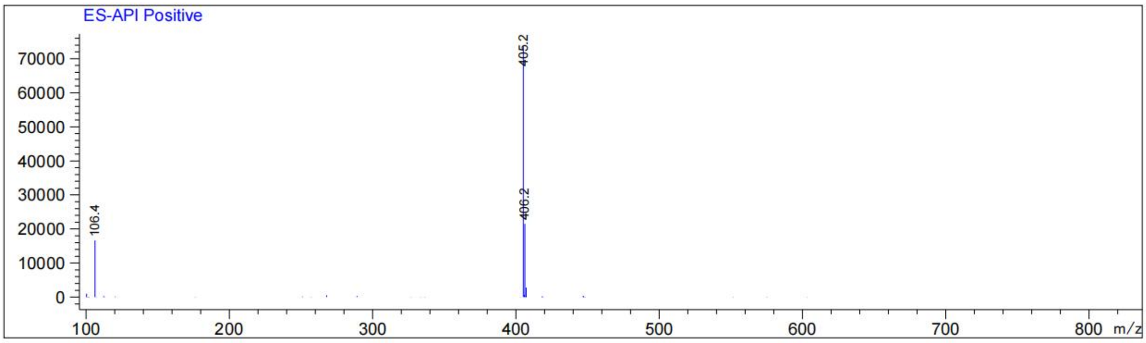

Figure S52 LCMS report of compound **2**

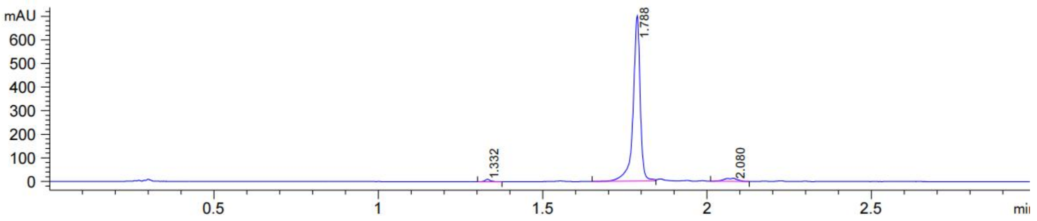

RetTim	Width	Area	Height	Area%
1.33	0.02	11.56	10.19	1.04
1.79	0.02	1059.05	702.22	95.63
2.08	0.04	36.83	13.32	3.33

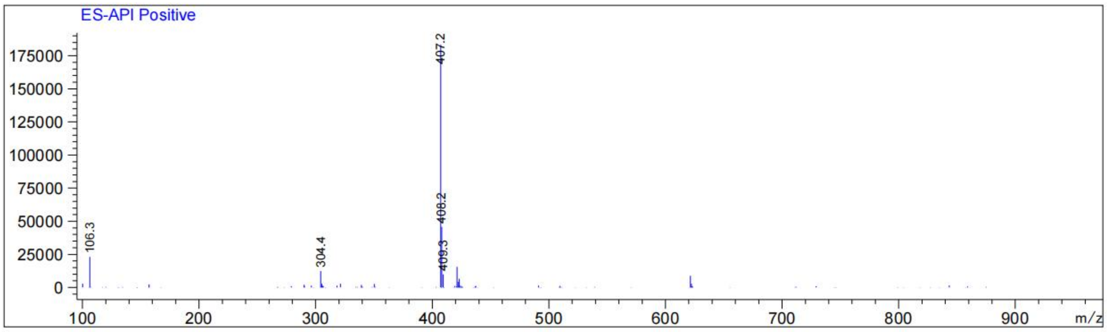

Figure S53 LCMS report of compound **3**
